# Supplementary material for: Factors influencing the implementation of labour companionship: formative qualitative research in Thailand
Source: BMJ Open. 2022 May 26;12(5):e054946. doi: 10.1136/bmjopen-2021-054946 (PMC9327797; doi:10.1136/bmjopen-2021-054946)
Supplement: Supplementary data [file bmjopen-2021-054946supp001.pdf]

Appendix 1. In-depth interview guides

This file contains the in-depth interview guides for all participant groups in this study:

1. Pregnant women (page 2)
2. Postpartum women (page 6)
3. Potential companions before birth (page 11)
4. Potential companions after birth (page 15)
5. Health workers (page 19)

In-depth interview: pregnant women

Step 1: Introduce yourself to the participant. Describe the purpose of the interview and how information will be used. Obtain oral/written consent.
Step 2: Ask the participant to identify herself. Interviewer: fill out the information below prior to beginning the interview.
Step 3: Conduct the interview. Please remember to audio record the interview.

Interview place: ________________________________

Interview date: _________________________________

Start time: _____________________________________

End time: ______________________________________

Interviewer name: _________________________________

Interview identification: ___________________________________

Participant information

Duration of current pregnancy (weeks/months, please label):________________________________

Age (years): ____

Marital status (Single, married/cohabitating, divorced, widowed): _____________________

Occupation (write in): ____

Part 1. Values and needs surrounding the childbirth period

1. What are some things you are doing to prepare for your birth?
    a. Probe: who is helping you to prepare? How are they helping?

2. Thinking about your pregnancy, what are some of the things you value most? Why?
    a. Probe: to keep yourself healthy?
    b. Probe: to keep your baby healthy?

3. Thinking to the future about your birth, what are some of the things that are most important to you?
    a. Probe: What will you need from your doctors and midwives to make sure this happens?
    b. Probe: what will you need from your family to make sure this happens?
    c. Where are you planning to give birth? Why?
        i. Did anyone help you decide where to give birth? Who? How did they help?

Part 2. Prenatal education

4. Thinking about when you go to your antenatal care visits, what are some of the things that are most important to you to learn about?
    a. Probe: What are some of the most important things you have learned during antenatal care? Why are they important?
    b. Probe: How do you think antenatal care could be improved?
        i. What do you think is missing from your antenatal care visits?
        ii. Are there any things that you would remove or change during your antenatal care visits?

Part 3. Preferences and decision-making processes regarding mode of childbirth

5. Could you tell me about the different ways that women can give birth?
    a. Probe: How did you learn about these options?

6. What do you think about vaginal birth and caesarean section?

7. What do you think are some of the positive things about vaginal birth?
    a. Probe: Why are these positive things?
    b. Probe: How did you learn about these positive things?

8. What are some of the negative things about vaginal birth?
    a. Probe: Why are these negative things?
    b. Probe: How did you learn about these negative things?

9. What do you think are some of the positive things about caesarean section?
    a. Probe: Why are these positive things?
    b. Probe: How did you learn about these positive things?

10. What are some of the negative things about caesarean section?
    a. Probe: Why are these negative things?
    b. Probe: How did you learn about these negative things?

11. How did you learn about vaginal birth and caesarean section?

12. How would you prefer to give birth? For example, caesarean or vaginal birth?
    a. Why do you prefer to give birth this way?
    b. How important is it for you to give birth this way? Why?
    c. Was anyone involved in helping you make a decision about how you prefer to give birth?
        i. Probe: Will your (husband/partner) influence this decision? How?
        ii. Probe: Will your family influence this decision? How?
        iii. Probe: Will your friends influence this decision? How?
        iv. Probe: Will your doctor or midwife influence this decision? How?
        v. Probe: Will the media influence this decision? How?

13. Do you feel like you have enough information to understand the options that you have for how to give birth? Why or why not?
    a. Probe: What other type of information about different modes of childbirth would you be interested to learn about?
    b. At what point during your pregnancy would you like to receive this information?

14. Did you discuss your preference with your doctor or midwife? If so, what was the discussion like?

15. *A decision-analysis tool could help to educate women about their options for mode of birth and how to discuss their preferences with a doctor. Interviewer: show the woman the Vietnam decision analysis tool.*
    a. Would this type of tool be helpful to you? Why or why not?
    b. What type of information would you like to have included?
    c. *This type of decision tool can be paper based like this example, or could be an application for a phone*. Which of these options do you prefer and why?

16. Pregnancy and childbirth are exciting times but can also be scary for some women. Is there anything that you are afraid or nervous of during your pregnancy? Why or why not?
    Note to interviewer: if they bring up fear of pain, then probe about what pain management technique they have learned about.

    a. What about during your birth, is there anything that you are afraid of? Why or why not?
        i. Have you spoken to your doctor or midwife about these fears? Why or why not? What did they tell you?
        ii. Have you spoken to anyone else about these fears?
            1. *If yes*:
                a. Who did you speak to? Why did you choose to speak to this person?
                b. What type of advice did they give you?
            2. *If no*: Do you plan to speak to anyone about these fears? Why or why not?
        iii. What do you think could be done to help reduce this fear for you?

Part 4. Labour companionship

17. What do you need in order to have a positive experience when you go to the hospital for childbirth?
    d. What type of support do you think you need during labour and childbirth?

Interviewer read: A labour companion is a person of the woman's choice, who can help to provide emotional support to the woman during labour and childbirth. Typically, this person would be with the woman continuously throughout labour and childbirth. This person may be the woman's husband/partner, her mother, or a friend.

18. Do you think you will receive this type of support? Why or why not?

19. <u>Have you ever heard of someone providing this type of support?</u>

20. <u>What do you think of this type of support?</u>

21. <u>Do you know if labour companionship is allowed in the hospital you plan to give birth in?</u>
    e. *If labour companionship is not allowed*: What do you think are the reasons for not allowing a labour companion?
    f. Would you be allowed a labour companion if you requested it? Why or why not?
    g. In your opinion, what changes do you think the hospital could make in order to make it more comfortable for women to have a labour companion?

22. <u>Do you think you would want to have a labour companion for your upcoming birth? Why or why not?</u>
    h. What type of information or education would YOU need before deciding if you wanted to have a labour companion to support you?

23. <u>If you were to have a labour companion with you:</u>
    i. What would you expect from this person?
    j. When would you want this person to be with you in the hospital (probe: the whole time, only during labour but not during the birth, something else?)
    k. Who would you prefer this person to be? Why?
    l. When would you like to start talking to your labour companion about their role during your labour and childbirth?
  iii. Probe: at what month during your pregnancy?

24. <u>What type of information or education do you think a labour companion would need to be able to support you?</u>

Thank you so much for your time. Is there anything else that you would like to share with me today about anything we talked about?

In-depth interview: postpartum women

Step 1: Introduce yourself to the participant. Describe the purpose of the interview and how information will be used. Obtain oral/written consent.
Step 2: Ask the participant to identify herself. Interviewer: fill out the information below prior to beginning the interview.
Step 3: Conduct the interview. Please remember to audio record the interview.

Interview place: ________________________________

Interview date: ________________________________

Start time: ____________________________________

End time: _____________________________________

Interviewer name: _________________________________

Interview identification: ____________________________________

Participant information

Duration of current pregnancy (weeks/months, please label):________________________________

Age (years): ____

Marital status (Single, married/cohabitating, divorced, widowed): _____________________

Occupation (write in): ____

Rungreangkulkij S, *et al. BMJ Open* 2022; 12:e054946. doi: 10.1136/bmjopen-2021-054946

Part 1. Values and needs surrounding the childbirth period

25. Can you tell me about your recent birth?
    a. Where did you give birth?
    b. Were you planning to give birth there? Why or why not?
    c. How did you make the decision about where to give birth?
        i. Who was involved in the decision about where to give birth?
        ii. Probe: Did you influence this decision? How?
        iii. Probe: Did your family or her family influence this decision? How?
        iv. Probe: Did your friends or her friends influence this decision? How?
        v. Probe: Did the doctor or midwife influence this decision? How?
        vi. Probe: Did the media influence this decision? How?

26. <u>How did you feel about your overall experience of giving birth?</u>
    a. How would you describe your birth experience? Why?

27. <u>Satisfaction</u>
    a. How satisfied are you with the type of care you received during labour and childbirth?
    b. Can you give me an example of something you are very satisfied with?
    c. Can you give me an example of something you are NOT satisfied with?

28. <u>How well do you feel that your healthcare provider respected your opinions about care during labour and childbirth?</u>
    a. Can you give me an example of a time when your healthcare provider respected your opinions about your care during labour and childbirth?
        i. How did this make you feel?
    b. Can you give me an example of a time when your healthcare provider did NOT respect your opinions about your care during labour and childbirth?
        i. Probe: Or, an example of a time during labour and childbirth when someone else was making decision without talking with you?
            1. How did this make you feel?

Part 2. Decision-making processes regarding mode of childbirth

29. <u>Could you tell me about the different ways that women can give birth?</u>
    a. Probe: How did you learn about these options?

30. <u>What about for your most recent birth – how did you give birth? For example, caesarean or vaginal birth?</u>
    a. Is this the way that you preferred to give birth? Why or why not?
    b. Probe: How did you come to give birth in this way?
        i. Probe: Did your (husband/partner) influence this decision? How?
        ii. Probe: Did your family influence this decision? How?
        iii. Probe: Did your friends influence this decision? How?
        iv. Probe: Did your doctor or midwife influence this decision? How?
            1. Did you discuss this decision with your doctor or midwife? If so, what was the discussion like?
        v. Probe: Did the media influence this decision? How?

31. <u>What do you think about vaginal birth and caesarean section?</u>
    a. What do you think are some of the positive things about vaginal birth?

Rungreangkulkij S, *et al. BMJ Open* 2022; 12:e054946. doi: 10.1136/bmjopen-2021-054946

            i. Probe: Why are these positive things?
            ii. Probe: How did you learn about these positive things?
    b. What are some of the negative things about vaginal birth?
            i. Probe: Why are these positive things?
            ii. Probe: How did you learn about these positive things?
    c. What do you think are some of the positive things about caesarean section?
            i. Probe: Why are these positive things?
            ii. Probe: How did you learn about these positive things?
    d. What are some of the negative things about caesarean section?
            i. Probe: Why are these positive things?
            ii. Probe: How did you learn about these positive things?

32. <u>How did you learn about vaginal birth and caesarean section?</u>

33. <u>Did you feel like you had enough information to understand the options that you had for how to give birth? Why or why not?</u>
    a. Probe: What other type of information about different modes of childbirth would you have been interested to learn about?
    b. At what point during your pregnancy would you like to receive this information?

Part 3. Prenatal education

34. <u>Thinking back to your antenatal care visits, what were some of the things that were most important to you to learn about?</u>
    a. Probe: What are some of the most important things you have learned during antenatal care? Why are they important?
    b. Probe: How do you think antenatal care could be improved?
        i. What do you think was missing from your antenatal care visits?
        ii. Are there any things that you would remove or change during your antenatal care visits?

Part 4. Decision-aids

35. <u>Where did you get most of the information to educate you about what to expect while giving birth?</u>
    a. What type of topics did you learn about?
    b. How well do you feel these educational materials prepared you to give birth? Why?
        i. Probe: can you give me an example of something that you felt very well prepared for?
        ii. Probe: can you give me an example of something that you did NOT feel well prepared for?
    c. Overall, how well prepared did you feel to give birth? Why?

Rungreangkulkij S, *et al. BMJ Open* 2022; 12:e054946. doi: 10.1136/bmjopen-2021-054946

36. Did you feel like you had sufficient time to talk to your doctor or midwife about any concerns that you had about labour and childbirth? Why or why not?
    a. Can you give me an example of a time when you felt that you were able to discuss your questions or concerns with your doctor or midwife?
    b. Can you give me an example of a time when you felt that you were NOT able to discuss your questions or concerns with your doctor or midwife?

37. *A decision-analysis tool could help to educate women about their options for mode of birth and how to discuss their preferences with a doctor.* Interviewer: show the woman the Vietnam decision analysis tool.
    a. Would this type of tool be helpful to you? Why or why not?
    b. What type of information would you like to have included?
    c. This type of decision tool can be paper based like this example, or could be an application for a phone. Which of these options do you prefer and why?

Part 5. Labour companionship

38. Who was with you while you were in labour?
    a. Probe: was your husband/partner with you?
        i. *If yes*:
            1. What was he doing while you were in labour and giving birth?
            2. Was he in the room with you? Why or why not?

    b. Probe: were any of your family members or friends with you?
        i. *If yes*:
            1. Who was with you?
            2. What were they doing while you were in labour and giving birth?
            3. Were they in the room with you? Why or why not?

39. What type of support do you think that you need during labour and childbirth while in the facility?
    a. Did you feel that you were supported during labour and childbirth? Why or why not?
    b. Can you give me an example of when you did feel supported?
    c. Can you give me an example of when you did not feel supported?
    d. What could have been done to improve your experience of support during labour and childbirth?
        i. Probe: Why do you think this is important?

Interviewer to read: Some women have a person with them during labour and childbirth, and we call this person a "labour companion". A labour companion is typically a woman's husband, boyfriend, sister, mother, or friend, who stays with the woman throughout labour and childbirth. They help the woman by providing emotional support, praising her and reassuring her.

40. What do you think about this type of support?

41. Would you have wanted someone to support you in this way during your labour and childbirth? Why or why not?

42. Who would you want this person to be?
    a. Probe: your husband/partner? Why?
    b. Probe: a sister or friend? Why?
    c. Probe: a mother or mother-in-law? Why?

43. When would you want to have this person with you?
    a. Probe: all of the time during labour and childbirth?
    b. Probe: only some of the time (e.g. only during labour, but not the birth)

44. How do you think having a labour companion might be helpful?

45. What are some challenges to having a labour companion?

46. Do you know if labour companionship is allowed in the hospital you gave birth in?
    a. *If labour companionship is not allowed:* What do you think are the reasons for not allowing a labour companion in this hospital?

47. What changes do you think the hospital could make to make it more comfortable for women to have a labour companion?

48. Do you have any other comments or feedback about labour companionship?

Thank you so much for your time. Is there anything else that you would like to share with me today about anything we talked about?

In-depth interview: partner / potential companion (before birth)

Step 1: Introduce yourself to the participant. Describe the purpose of the interview and how information will be used. Obtain oral/written consent.
Step 2: Ask the participant to identify herself. Interviewer: fill out the information below prior to beginning the interview.
Step 3: Conduct the interview. Please remember to audio record the interview.

Interview place: ______________________________

Interview date: ______________________________

Start time: ______________________________

End time: ______________________________

Interviewer name: ______________________________

Interview identification: ______________________________

<u>Participant information</u>

Relationship with pregnant woman: ______________________________

Age (years): ______________________________

Marital status (Single, married/cohabitating, divorced, widowed): ____________________

Occupation (write in): ______________________________

<u>Information on pregnant women</u>

N° of women's interview: ______________________________

Duration of current pregnancy:______________________________

Rungreangkulkij S, *et al. BMJ Open* 2022; 12:e054946. doi: 10.1136/bmjopen-2021-054946

Part 1. Values and needs surrounding the childbirth period

49. What are some things you are doing to prepare for your (wife/partner/daughter/sister…) birth?
    a. Probe: who is helping you to prepare? How are they helping?

50. Thinking about your wife/partner/sister pregnancy, what are some of the things you value most? Why?
    a. Probe: to keep her healthy?
    b. Probe: to keep the baby healthy?
    c. Probe: for yourself personally?

51. Thinking to the future about your (wife/partner/daughter/sister…) birth, what are some of the things that are most important to you?
    a. Probe: What will you need from your doctors and midwives to make sure this happens?
    b. Probe: what will you need from your family to make sure this happens?

52. Place of birth
    a. Where is your (wife/partner/daughter/sister…) planning to give birth? Why?
    b. How did she make the decision about where to give birth?
        i. Did anyone help her make the decision about where to give birth? Who? How?

Part 2. Decision-making processes regarding mode of childbirth

53. Could you tell me about the different ways that women can give birth?
    a. Probe: How did you learn about these options?

54. What do you think about vaginal birth and caesarean section?
    a. What do you think are some of the positive things about vaginal birth?
    b. What are some of the negative things about vaginal birth?
    c. What do you think are some of the positive things about caesarean section?
    d. What are some of the negative things about caesarean section?

55. How would you prefer that your (wife/partner/daughter/sister…) gives birth? For example, caesarean or vaginal birth?
    a. Probe: Why do you prefer this way?
    b. Does your (wife/partner/daughter/sister…) also prefer to give birth this way? Why or why not?

56. Did your (wife/partner/daughter/sister…) decide about how she will give birth? For example, caesarean or vaginal birth?
    a. Probe: Who makes this decision?
       (note: the decision about mode of birth has not yet been made, or if he/she responds that they don't know to question 7, please instead ask: "How do you think she would plan to make this decision?")
        i. Probe: Did you influence this decision? How?
        ii. Probe: Did your family or her family influence this decision? How?
        iii. Probe: Did your friends or her friends influence this decision? How?
        iv. Probe: Did the doctor or midwife influence this decision? How?
        v. Probe: Did the media influence this decision? How?

Part 3. Prenatal education

57. Have you been to any antenatal care visits with your (wife/partner/daughter/sister…) ? Why or why not?
    a. *If yes:* Thinking about when you went to the antenatal care visits, what are some of the things that are most important to you?
        vi. Probe: What are some of the most important things you have learned during antenatal care? Why are they important?
        vii. Probe: How do you think antenatal care could be improved?
        viii. What do you think is missing from the antenatal care visits?
        ix. Are there any things that you would remove or change during the antenatal care visits? What are they and why would you change?

58. Thinking about antenatal care visits, what are some of the things that are most important to you to learn about?
    a. Probe: What are some of the most important things you have learned during antenatal care? Why are they important?
    b. Probe: How do you think antenatal care could be improved?

Part 4. Decision-aids

59. How did you learn about vaginal birth and caesarean section?

60. Do you feel like you have enough information to understand the options that women have for how to give birth? Why or why not?
        i. Probe: What other type of information about different modes of childbirth would you be interested to learn about?
        ii. At what point during your (wife/partner/sister…'s) pregnancy would you like to receive this information?

61. Pregnancy and childbirth are exciting times but can also be scary. Is there anything that you are afraid of or nervous about pregnancy or childbirth? Why or why not?
    a. What about during the birth, is there anything that you are afraid of? Why or why not?

    b. *If yes*:
        i. Have you spoken to anyone about these fears? Why or why not?
            1. *If yes*: What did they tell you?
            2. *If no*: Do you plan to speak to anyone about these fears? Why or why not?
        ii. What do you think could be done to help reduce this fear for you?

Part 5. Labour companionship

62. Do you plan to go to the hospital with your (wife/partner/daughter/sister…) when she gives birth? Why or why not?

63. If you do go to the hospital when your (wife/partner/ daughter/sister…) gives birth, what do you need in order to have a positive experience?

64. What type of support do you think your (wife/partner/ daughter/sister…) needs during labour and childbirth?
    a. Do you think she will receive this type of support? Why or why not?

Rungreangkulkij S, *et al. BMJ Open* 2022; 12:e054946. doi: 10.1136/bmjopen-2021-054946

Interviewer read: A labour companion is a person of the woman's choice, who can help to provide emotional support to the woman during labour and childbirth. Typically, this person would be with the woman continuously throughout labour and childbirth. This person may be the woman's husband/partner, her mother, or a friend.

65. Have you ever heard of someone providing this type of support?

66. What do you think of this type of support?

67. Do you know if labour companionship is allowed in the hospital your (wife/partner/ daughter/sister…) plan to give birth in?
    a. *If labour companionship is not allowed*: What do you think are the reasons for not allowing a labour companion?
    b. Would your (wife/partner/sister…) be allowed a labour companion if she requested it? Why or why not?

68. Have you ever provided this type of support before? (*If yes*: Could you tell me more about this?)

69. Do you think your (wife/partner/ daughter/sister…) would want to have a labour companion for her upcoming birth? Why or why not?

70. If your (wife/partner/ daughter/sister…) were to have a labour companion with her, who do you think she would prefer this person to be? Why?

71. *If your (wife/partner/ daughter/sister…) were to have a labour companion with her, what do you think she would expect from this person?*

72. *Would you be interested in being a labour companion to your (wife/partner/* daughter/*sister…)? Why or why not?*

73. What would you need in order to be a good labor companion?
    a. What do you need from the woman?
    b. What do you need from the nurses and doctors?
    c. What do you need from the hospital?

74. What type of information or education do you think a labour companion would need to be able to support her?
    a. When during pregnancy do you think a woman or a nurse should start talking to a potential labour companion about their role during labour and childbirth?

75. In your opinion, what changes do you think the hospital could make in order to make it more comfortable for women to have a labour companion?

Thank you so much for your time. Is there anything else that you would like to share with me today about anything we talked about?

In-depth interview: partner / potential companion (postpartum)

Step 1: Introduce yourself to the participant. Describe the purpose of the interview and how information will be used. Obtain oral/written consent.
Step 2: Ask the participant to identify herself. Interviewer: fill out the information below prior to beginning the interview.
Step 2: Conduct the interview. Please remember to audio record the interview.

Interview place: ________________________________

Interview date: _________________________________

Start time: ____________________________________

End time: _____________________________________

Interviewer name: _________________________________

Interview identification: ___________________________________

Participant information

Relationship with pregnant woman: ________________________________

Age (years): ____

Marital status (Single, married/cohabitating, divorced, widowed): _____________________

Occupation (write in): ____

Information on pregnant women

N° of women's interview: __________________________________

Woman's date of most recent birth:________________________________

Part 1. Values and needs surrounding the childbirth period

Note to interviewers: only ask questions 1-3 at Khon Kaen University Hospital or any hospital that allows companions.

76. How did you feel about your overall experience of your (wife/partner/daughter/sister…) giving birth?
    a. How would you describe your experience? Why?

77. Satisfaction
    a. How satisfied are you with the type of care your (wife/partner/daughter/sister…) received by your (wife/partner/daughter/sister…)during labour and childbirth?
    b. Can you give me an example of something you are very satisfied with?
    c. Can you give me an example of something you are NOT satisfied with?

78. How well do you feel that your (wife/partner/daughter/sister…) healthcare provider respected your opinions about care during labour and childbirth?
    a. Can you give me an example of a time when your (wife/partner/daughter/sister…) healthcare provider respected your opinions about care during labour and childbirth?
    b. Can you give me an example of a time when your (wife/partner/daughter/sister…) healthcare provider did NOT respect your opinions about your care during labour and childbirth?
        i. Probe: Or, an example of a time during labour and childbirth when someone else was making decision without talking with you or your (wife/partner/daughter/sister…)?

79. Place of birth
    a. Was she planning that she would give birth in this facility? Why or why not?
    b. How was the decision made about where she gave birth?
        i. Who was involved in the decision-making?

80. Thinking back to your wife/partner/sister birth, what are some of the things you value most? Why?
    a. Probe: to keep her healthy?
    b. Probe: to keep the baby healthy?
    c. Probe: for yourself personally?

Part 2. Decision-making processes regarding mode of childbirth

81. Could you tell me about the different ways that women can give birth?
    a. Probe: How did you learn about these options?

82. What about for your (wife/partner/daughter/sister…) most recent birth – how did she give birth? For example, caesarean or vaginal birth?
    a. Is this the way that you preferred your (wife/partner/daughter/sister…) would give birth? Why or why not?

    b. Probe: How did you decide that you preferred her to give birth in this way?
        i. Probe: Did your (wife/partner/daughter/sister…) influence your opinion? How?
        ii. Probe: Did your family influence your opinion? How?
        iii. Probe: Did your friends influence your opinion? How?

Rungreangkulkij S, *et al. BMJ Open* 2022; 12:e054946. doi: 10.1136/bmjopen-2021-054946

iv. Probe: Did your (wife/partner/daughter/sister…) doctor or midwife influence your opinion? How?
v. Probe: Did the media influence your opinion? How?

c. Did you discuss your opinion with your (wife/partner/daughter/sister…) doctor or midwife? If so, what was the discussion like?

83. Who decided about how she would give birth?
    a. Probe: who was involved in the decision-making?
    b. Probe: Did you influence this decision? How?
    c. Probe: Did your family or her family influence this decision? How?
    d. Probe: Did your friends or her friends influence this decision? How?
    e. Probe: Did the doctor or midwife influence this decision? How?
    f. Probe: Did the media influence this decision? How?

84. How did you learn about vaginal birth and caesarean section?

85. Did you feel like you had enough information to understand the options that your (wife/partner/daughter/sister) had for how to give birth? Why or why not?

86. Did you feel like you had sufficient time to talk to your (wife/partner/daughter/sister…) doctor or midwife about any concerns that you had about your (wife/partner/daughter/sister…) labour and childbirth? Why or why not?
    a. Can you give me an example of a time when you felt that you were able to discuss your questions or concerns with your (wife/partner/daughter/sister…) doctor or midwife?
    b. Can you give me an example of a time when you felt that you were NOT able to discuss your questions or concerns with your (wife/partner/daughter/sister…) doctor or midwife?

87. Pregnancy and childbirth are exciting times but can also be scary. Is there anything that you were afraid of or nervous about the childbirth?

88. *A decision-analysis tool could help to educate women about their options for mode of birth and how to discuss their preferences with a doctor.* Interviewer: show the woman the Vietnam decision analysis tool.
    a. Would this type of tool be helpful to you? Why or why not?
    b. What type of information would you like to have included?
    c. This type of decision tool can be paper based like this example, or could be an application for a phone. Which of these options do you prefer and why?

Part 3. Labour companionship

89. Were you at the hospital with your (wife/partner/daughter/sister…) when she gave birth? Why or why not?

90. If you have gone to the hospital when your (wife/partner/ daughter/sister…) gave birth, what did you need in order to have a positive experience?

91. What type of support do you think your (wife/partner/ daughter/sister…) needed during labour and childbirth?
    a. Do you think she will received this type of support? Why or why not?

Interviewer read: A labour companion is a person of the woman's choice, who can help to provide emotional support to the woman during labour and childbirth. Typically, this person would be with the woman continuously throughout labour and childbirth. This person may be the woman's husband/partner, her mother, or a friend.

92. Have you ever heard of someone providing this type of support?

93. What do you think of this type of support?

94. Do you know if labour companionship was allowed in the hospital your (wife/partner/ daughter/sister…) gave birth in?
    a. *If labour companionship was not allowed*: What do you think are the reasons for not allowing a labour companion?
    b. Had your (wife/partner/sister…) be allowed a labour companion if she had requested it? Why or why not?

95. Have you ever provided this type of support before?
    a. *If yes*: Could you tell me more about this?

96. Do you think your (wife/partner/ daughter/sister…) would have wanted to have a labour companion for her birth? Why or why not?

97. If your (wife/partner/ daughter/sister…) were to have a labour companion with her, who do you think she would prefer this person to be? Why?

98. If your (wife/partner/ daughter/sister…) were to have a labour companion with her, what do you think she would expect from this person?

99. Would you be interested in being a labour companion to your (wife/partner/ daughter/sister…)? Why or why not?

100. What would you need in order to be a good labor companion?
    a. What do you need from the woman?
    b. What do you need from the nurses and doctors?
    c. What do you need from the hospital?

101. What type of information or education do you think a labour companion would need to be able to support her?
    a. When do you think a woman should start talking to a potential labour companion about their role during labour and childbirth? (probe: at what month during the pregnancy?)

102. In your opinion, what changes do you think the hospital could make in order to make it more comfortable for women to have a labour companion?

Thank you so much for your time. Is there anything else that you would like to share with me today about anything we talked about?

In-depth interview: providers

Step 1: Introduce yourself to the participant. Describe the purpose of the interview and how information will be used. Obtain oral/written consent.
Step 2: Complete sociodemographic information about the participant.
Step 3: Conduct the interview. Please remember to audio record the interview.

Interview place: ______________________________

Interview date: ______________________________

Start time: ______________________________

End time: ______________________________

Interviewer name: ______________________________

Interview identification: ______________________________

Participant information

Name of health facility currently employed at (write in): ____________________

Cadre and position (write in): ______________________________

Number of years working at current health facility: ______________________________

Number of years as a (doctor/midwife/nurse) in total: ______________________________

Age (years): ______________________________

Marital status (Single, married/cohabitating, divorced, widowed): ____________________

Rungreangkulkij S, *et al. BMJ Open* 2022; 12:e054946. doi: 10.1136/bmjopen-2021-054946

Decision-making processes regarding mode of childbirth

1. Could you describe what you think quality care provided during childbirth is?
   a. Could you give me an example of a situation when this kind of care was provided by you?
      i. Why do you think you were able to provide quality care in this situation?
      ii. How did your colleagues support you to provide quality care?

   b. Could you give me an example of a situation when this kind of care was NOT provided by you or by a coworker?
      i. Why do you think you weren't able to provide quality care in this situation?
      ii. Did you feel like your colleagues supported you in this situation? Why or why not?

2. In your health facility, how are decisions made about whether a woman will give birth vaginally or by caesarean section?
   i. Who is involved in making the decision, and what roles do they play?
   ii. In your health facility, what are some of the clinical indications for caesarean section?
   iii. Other than clinical indications for caesarean section, what factors might influence if a woman has a caesarean section?

3. Is assisted vaginal delivery (e.g. by vacuum or forceps) used in your facility?
   i. Probe: why or why not?
   ii. Probe: were you trained on how to provide assisted vaginal delivery? Please explain.

4. In your facility, do you think that women prefer to give birth by caesarean section or vaginally? Please explain.

5. In your health facility, how do you manage women who request to have an elective caesarean section?
   i. Why do you think women may request to have a caesarean section without a medical indication (e.g. elective caesarean)?
   ii. Who do you think influences women's decisions to have a caesarean section without a medical indication (e.g. elective caesarean)?

6. As a clinician, do you prefer for women to give birth vaginally or by caesarean section? Why?
   i. What are some of the benefits/challenges of caesarean section/vaginal birth?
   ii. Which do you think is safer: vaginal birth or caesarean section? Why?

7. In your opinion, are high rates of caesarean section a problem in your health facility? Why or why not?
   i. *Probe if yes:*
      i. Why do you think there are high rates of caesarean section in your facility?
      ii. Do you think that the caesarean section rate in your facility can be reduced? Why or why not?
      iii. Do you think that the caesarean section rate in your facility should be reduced? Why or why not?
      iv. What are the barriers to reducing high rates of caesarean section in your facility?

Rungreangkulkij S, *et al. BMJ Open* 2022; 12:e054946. doi: 10.1136/bmjopen-2021-054946

v. What could be done to reduce high rates of caesarean section in your facility?

ii. *Probe if no:*

i. Do you think that the caesarean section rate in your facility could be reduced? Why or why not?

ii. Do you think that the caesarean section rate in your facility should be reduced? Why or why not?

iii. What are the barriers to reducing high rates of caesarean section in your facility?

8. From your perspective, is a caesarean section more or less work for a healthcare provider, compared to a vaginal birth? Please explain.

9. In your opinion, do you think it is financially more profitable for providers or health facilities to conduct a caesarean section, compared to a vaginal birth?
    a. *Probe:* Why or why not?

Prenatal education and decision-analysis tool

Interviewer: The next section of this interview is about the type of health education about mode of birth that you think women would like to receive during antenatal care. I would like to ask you some questions about what you think about different topics of health education to be discussed during antenatal care.

10. In your opinion, do you think that women have sufficient knowledge about their options and the risks and benefits for different mode of birth? Why or why not?

11. In your practice, how do pregnant women they access information about their options for mode of birth?
    a. What do you think about these information resources?

12. What type of information do you think that women need to inform their preferences and decisions about their mode of birth?
    a. *Probe:* Risks of different methods, benefits of different methods, personal preferences

13. Do you think that groups of women may have different needs for information about mode of birth?
    a. *Probe if yes*: what groups of women do you think may need different information?
    b. *Probe if yes*: What type of information do you think that these women might need?

14. During antenatal care, do you (or providers conducting antenatal care in your facility) discuss with women whether they have a preference for vaginal birth or caesarean section?
    a. *IF YES, probe:* What do you discuss with the women?
    b. *IF NO, probe:* Do you think that discussing their preferences for vaginal birth or caesarean section could be helpful? Why or why not?

15. What information do you think could be included in prenatal education about vaginal birth and caesarean section?

16. At what point during a woman's pregnancy do you think they should receive this information about mode of birth? Why?

17. How often do you think women should receive this information about vaginal birth and caesarean section?
    a. *Probe:* Once? More than once?

18. How would do you think women should receive this information?
    a. *Probe:* Should she receive this information verbally, from her healthcare provider? Why or why not?
    b. *Probe:* Should she receive this information in a pamphlet or brochure? Why or why not?
    c. *Probe:* Should she receive this information using a computer or a mobile phone application? Why or why not?

(Interviewer: use the Vietnam decision-analysis tool as an example)
The next section of this interview is about using decision-tools (such as a computer, tablet or a smart phone) to help pregnant women with previous caesarean sections to understand their choices about mode of birth. By this, we mean whether the woman will have planned vaginal birth, trial of labour, or a caesarean section. These decision-tools would provide them with information about potential benefits and harms of the different options. They would be in addition to any regular counselling or discussions with healthcare providers. I would like to ask you some questions about what you think about these decision-tools.

19. *A decision-tool could provide descriptions of the health outcomes associated with planned vaginal birth, planned caesarean section, and emergency caesarean section. They can also ask questions about a woman's values and preferences for possible outcomes. Once this information is provided, the decision-tool can produce a recommended "preferred option", based on a woman's preferences. The woman could then bring this to her healthcare provider to discuss in more detail.* What do you think about this type of decision-tool?
    Does this description of a decision-tool sound like something that might be useful to you? Why or why not?
    a. What do you think are some of the benefits of using a decision-tool to help decide about how a woman will give birth?
        i. *Probe:* to you as a provider?
        ii. *Probe:* to the woman
    b. What do you think are some of the challenges of using a decision-tool to help decide about how a woman will give birth?
        iii. *Probe:* to you as a provider?
        iv. *Probe:* to the woman
    c. At what point during a woman's pregnancy would it be most helpful for her to have access to this type of decision-tool? Why?
    d. How might you use the results of the decision tool, or the woman's "preferred option", to discuss her options for mode of birth?
    e. Do you think that you would recommend that women use this type of decision-tool? Why or why not?

20. *These types of decision-tools can come in different formats. For example, on paper, a computer, a tablet, or a smart phone application.* What format do you think would be most helpful? Why?

Audit and feedback

Note to interviewer: use graphic of audit and feedback/Robson classification to explain to providers who don't understand. Consider using an example of maternal morbidity and mortality conference as example of audit and feedback.

*Interviewer: The next part of the study is about **using audit and feedback as a tool for quality improvement**. The purpose of audit and feedback is to encourage individuals and teams to follow professional standards or targets and to monitor changes and outcomes when these are used. During an audit and feedback process, an individual's or department's professional practice and/or performance is measured and compared to targets or professional standards. The results of this comparison are fed back to the individual by either a colleague, supervisor or third party, in the form of verbal or written communication. I would like to ask you some questions about what you think about audit and feedback.*

21. <u>Could you tell me about a time where you have been involved in an audit and feedback project?</u>
    a. *If they have been involved in an audit and feedback project:*
        i. What did you find helpful about the audit and feedback process?
        ii. What did you find challenging about the audit and feedback process?
        iii. What were the main things you learned from the audit and feedback process?
        iv. Overall, what was your opinion regarding the audit and feedback process?

22. <u>What areas of health do you think would be most interesting and relevant for audit and feedback? For example, this might include reasons for caesarean section, severe morbidity. Why are these interesting?</u>

Audit and feedback to improve obstetric care may include activities like critical case incident reviews, indications for caesarean section, time from decision to operation for caesarean section, decision-making processes for caesarean section, and appropriate management of complications. This may be done by reviewing individual patient records, labour and delivery logs, and observations of clinical practice.

23. <u>How would you feel about the idea of a regular audit and feedback process in your health facility to address rising caesarean section rates?</u>

24. What might be some of the benefits of audit and feedback may be related to caesarean section?

25. What might be some of the challenges of audit and feedback may be related to caesarean section?

26. Do you think starting an audit and feedback process may change people's behaviour in your department? Why or why not?

27. Do you think starting an audit and feedback process may change health outcomes? Why or why not?

28. What could be done in your health facility to ensure that audit and feedback is conducted in a supportive way that emphasises learning rather than punishing providers for certain behaviours?

29. How can audit and feedback be presented to you to ensure that any information gathered is "actionable" so that an individual can work to improve their practice?

30. <u>What type of person would be the most appropriate person to:</u>
    a. <u>Review medical records?</u>
    b. <u>Analyse the data and prepare a summary report?</u>

c. To present or discuss the report with you?
d. Probe: Would you prefer that this person were a colleague, supervisor, or someone external? Why?

31. Approximately how often do you think that audit and feedback processes should occur in your health facility? Please explain.

(Interviewer: do not ask these questions to antenatal nurses)
Interviewer: The next part of the study is about the ***audit and feedback tools for classifying caesarean sections****. These tools may be useful for healthcare providers and administrators to monitor which women are receiving caesarean sections, and also to help to compare caesarean section rates over time or across different health facilities and countries. This may help to design and implement interventions to make sure that an optimal caesarean section rate can occur in a specific health facility. I would like to ask you some questions about what you think of such audit and feedback tools. In order to understand drivers of rising Caesarean section rates, we need to have tools to monitor and compare caesarean section rates in a setting over time. One way to do this is the Robson classification system, which prospectively classifies women admitted for childbirth into one of ten groups.*

32. Have you heard of the Robson classification system before?
    a. *If yes:* Can you tell me what you know about the Robson classification system?
    b. *If no,* Do you know of any other classification systems to classify women giving birth?

33. *Conducting audit and feedback for caesarean section requires reviewing patient medical records and/or facility logs.* Could you tell me about how records are prepared and kept in your health facility?
    a. What is your perception regarding the completeness of labour and delivery records in your health facility?
        1. *Probe:* Do you think that labour and delivery records are complete and accurate for all or most women in your health facility? Why or why not?
    b. *Probe:* Who is responsible for recording in the medical records?
    c. *Probe:* In addition to the individual patient's record, how else is data collected and recorded on the labour and delivery ward?
    b. *Probe:* is there a facility-level logbook? If so, who is responsible for this? What type of data is recorded?

In-service training and implementation of clinical practice guidelines

34. How well do you feel your training prepared you for your current position? Please explain.

Rungreangkulkij S, *et al. BMJ Open* 2022; 12:e054946. doi: 10.1136/bmjopen-2021-054946

Interviewer: The next part of the study is about continuous training and implementation of clinical practice guidelines. This refers to the processes by which guideline recommendations are used to help healthcare providers make informed decisions about how and when to provide care in order to achieve the best health outcomes. I would like to ask you some questions about what you think about guideline implementation.

35. Are you aware of any clinical practice guidelines (algorithms/flowcharts/clinical protocols) related to obstetrics?
    a. *If yes,* which clinical practice guidelines are you familiar with?
        i. In your opinion, how valuable are the obstetrics clinical practice guidelines to your practice?
        ii. In your opinion, how accessible are these clinical practice guidelines to healthcare providers?
        iii. *Probe:* what could be done to improve the accessibility of clinical practice guidelines to other healthcare providers?
        iv. Could you describe the process of how obstetrics clinical practice guidelines are prioritised in your health facility?
        v. How are the clinical practice guidelines communicated to other healthcare providers in your facility?
        vi. How do you use clinical practice guidelines in your practice?
    b. *If no, probe:*
        i. How do healthcare providers in your facility make decisions about how to manage patients?
        ii. In your health facility, are clinical practice guidelines currently used in obstetrics?
        iii. In your opinion, what could be done to improve the accessibility of clinical practice guidelines to other healthcare providers?

36. *Imagine that your health facility will start a process of updating and implementing obstetrics clinical* practice guidelines (algorithms/flowcharts/clinical protocols). Who would need to support this initiative in order for it to be successful?
    a. *Probe:* Why would this person/these people need to support the initiative?

    b. *Probe:* How would this person/these people best support the initiative?

    c. What type of training would be helpful to ensure that all staff understand the clinical practice guidelines?
        i. What type of topics would you like to have covered during the training?
            1. Would you be interested to learn about how clinical practice guidelines were developed? Why or why not?
            2. Would you be interested to learn about the evidence behind the recommendations in clinical practice guidelines, such as the systematic reviews or clinical trials?
        ii. How long should the training last for?
        iii. How often should the training be repeated?
        iv. Where should the training be held (e.g. within the facility, outside the facility)?

    d. What resources would be needed in order to successful implement obstetrics clinical protocols?

e. In your opinion, what are some barriers to successful implementation of obstetrics clinical practice guidelines?

f. In your opinion, what are some facilitators to successful implementation of obstetrics clinical practice guidelines?

g. *Usually when clinical practice guidelines are implemented in health facilities, there are activities to evaluate if the guidelines are being implemented correctly and consistently*. What type of evaluation activities would be helpful to assess if obstetrics clinical practice guidelines were being implemented correctly and consistently?
    i. What format would be appropriate to feedback the evaluations to healthcare providers?
    ii. If meetings were held to feedback on the progress of obstetric clinical practice guidelines implementation, what would you like to hear discussed?
        1. Who would attend these meetings and why?
        2. How often would these meetings be held?

37. In your opinion, how important is providing pain relief for women during labour (vaginal birth only)? Why?

    a. In your facility, what pain relief options are there for women during labour (vaginal birth only)? Probe: pharmacological and non-pharmacological methods

    b. In your opinion, how important is it for women to walk around during labour (vaginal birth only)? Why?

    c. In your opinion, how important is it for women to be able to sit upright during labour (vaginal birth only)? Why?

Opinion leader education

Interviewer: The next part of the study is about using opinion leaders in a specific health facility to act as champions for change. Opinion leaders are influential individuals who are nominated by their peers to change the culture and norms of healthcare provider peer groups. For example, these individuals may be responsible for adapting clinical guidelines to a specific health facility context, and identifying measures to ensure quality improvement. I would like to ask you some questions about what you think about the use of opinion leaders in your health facility.

38. <u>What do you think are the characteristics of a good opinion leader?</u>

39. <u>What do you think about the idea of using opinion leaders to adapt clinical guidelines to your health facility?</u>

40. <u>What type of healthcare provider would be most appropriate to act as an opinion leader for caesarean section? *(probe: nurse/midwife/doctor, what level of training)*</u>

41. <u>How do you think an opinion leader would be received by other healthcare providers in your health facility?</u>

Rungreangkulkij S, *et al. BMJ Open* 2022; 12:e054946. doi: 10.1136/bmjopen-2021-054946

42. What challenges do you think an opinion leader would face if they tried to adapt and implement clinical guidelines in your health facility?

43. What type of *training* would an opinion leader need to succeed?

44. What resources would an opinion leader need to succeed?

Organization and relationships in the facility

45. Could you please describe for me what the relationship that you have with your peers is like?
    a. Could you tell me about a time when your peers supported you?
    b. Could you tell me about a time when your peers did not support you?
    c. If you are struggling to meet the demands of your work, can you look to your peers for help? How so?
    d. In your opinion, are men and women treated equally in your work place? Why or why not?

46. In your opinion, how well do doctors and midwives work together in general?
    a. How well do you think doctors and midwives communicate?
    b. What are some of the challenges in having midwives and doctors work together?
    c. Can you tell me about a time when midwives and doctors did NOT work together?
        i. Why do you think this happened?
    d. Can you tell me about a time when midwives and doctors worked very well together?
        i. Why do you think this happened?

47. Overall, how supportive do you feel that your work environment is? Please explain.

48. How do you feel about the current environment around malpractice lawsuits and legal liability for doctors?
    a. Do you feel that the health system or your health facility would support you in a legal case?
    b. How do you think that the legal environment may influence your own, or your colleagues', medical practice?

49. Do you feel afraid of malpractice lawsuits in your current work? Please explain.

50. What strategies do you employ to minimise the risk of a malpractice lawsuit?
    a. Do you think these strategies are reasonable?

Labour companionship

Interviewer: The next part of the study is about the type of support that women could receive during childbirth in a health facility. In some settings, a "labour companion" can provide this type of support. A labour companion is a person of the woman's choice, for example her husband, her sister, her mother, her friend, or a doula, who stays with the woman throughout the duration of labour and childbirth. I would like to ask you some questions about what you think about support from a companion during childbirth.

51. What type of support do you think women need during labour and childbirth?
    a. Do you think that women in your hospital receive this kind of support you have described? Why or why not?

52. What do you know about labour companionship?
    a. What are the benefits of labour companionship?
        i. *Probe*: What are benefits for the woman?
        ii. *Probe*: What are benefits for the providers?
        iii. *Prove*: What are benefits for the companion?
    b. Are there any harms of labour companionship?
        i. *Probe*: What are harms for the woman?
        ii. *Probe*: What are harms for the providers?
        iii. *Probe*: What are harms for the companion?

53. Do you have any previous experience with working in a hospital that offered labour support?
    i. If yes, what was this experience like for you as a provider?

54. Do you know if labour companionship is allowed in this hospital?
    a. *If labour companionship is not allowed:* What do you think are the reasons for not allowing a labour companion in this hospital?

55. How could labour companionship be implemented in your hospital or other hospitals like this?
    a. What would be the main challenges to implementing labour companionship?
    b. Who do you think women would prefer as a labour companion? Why?
    c. As a provider, what are your expectations from a woman's labour companion?
    d. When would a labour companion be able to be with the woman in the hospital?
    e. What would the role of the labour companion be?
        i. How could the labour companion's roles be communicated to them?
    f. At what point during the care process should women and providers start talking about labour companionship and the role of the companion?
    g. What type of information or education do you think a labour companion would need to be able to support you?
    h. How could we ensure that the companion is a person of the woman's choice, and not someone selected for her by someone else?
    i. What changes do you think the hospital could make to make it more comfortable for women to have a labour companion?
    j. If labour companionship is to be implemented in this hospital, what would ensure successful implementation?
        a. What could be done to ensure that labour companionship was sustainable in the long-term?
