# Supplementary material for: Factors influencing the implementation of labour companionship: formative qualitative research in Thailand
Source: BMJ Open. 2022 May 26;12(5):e054946. doi: 10.1136/bmjopen-2021-054946 (PMC9327797; doi:10.1136/bmjopen-2021-054946)
Supplement: Supplementary data [file bmjopen-2021-054946supp002.pdf]

Appendix 2. Readiness assessment

1.	Name of hospital	
2.	Hospital code	

This activity is part of the readiness assessment, to explore factors to be assessed, considered and integrated into implementation plans. There are five components of this readiness assessment:

1. *Inventory of physical space and readiness;*
2. *Health workforce and model of care;*
3. *Protocols and guidelines for managing clinical care during labour and childbirth;*
4. *Continuous education and quality improvement*
5. *Assessment of data availability and access for audit and feedback; and*
6. *Understanding of labour companionship in practice.*

For each of the study health facilities, please have a member of the research team visit to conduct an observation of the labour ward and medical records. It may be most appropriate for this person to have some clinical knowledge. We expect that this activity will take approximately four to six hours to complete (depending on how busy the facility is). Prior to conducting the readiness assessment, please ensure that all members of the maternity care unit at each health facility are briefed on the purpose of the activity, what the readiness assessment will entail, and how they may be of assistance. This will help to ensure that the readiness assessment (and other research activities) will be welcomed by the unit.

Initial inventory

3.	# of Deliveries (1 October 2019 – 31 January 2020)	
4.	No of delivery handled exclusively by a certain obstetrician (private)	
5.	Women not handled exclusively by a certain obstetrician (non-private)	
6.	No. of Caesarean section (1 October 2019 – 31 January 2020)	
7.	Elective (pre-labour c-section)<br>*Note: if this information is not readily available, then please ignore for now.*	
8.	Emergency (intrapartum c-section)	
9.	No. of vaginal instrumental delivery (1 October 2019 – 31 January 2020)	

Rungreangkulkij S, *et al. BMJ Open* 2022; 12:e054946. doi: 10.1136/bmjopen-2021-054946

Part 1. Inventory of physical space and resources

Please observe the physical space of the labour, delivery and postnatal wards. If these are in separate areas (e.g.: women in latent labour in a labour ward, women in active labour in a separate room/delivery ward, separate postnatal ward), please assess both areas according to all points below. Please provide a narrative description of the wards, as well as a visual depiction.

Description of the physical space

Please detail:

# of beds in labour /admission room (1st stage of labour room):	
# of beds in labour / childbirth room (2nd stage of labour room):	
# Ultrasound machine in the labour room (indicate number working)	
# Electronic fetal heat rate monitor (CTG) (indicate number of devices working)	

Description of any curtains, dividers or other means of protecting a woman's privacy

Description of the potential for crowding. For example, how many beds are present? Are they currently or usually full? What happens if there is overcrowding?

Rungreangkulkij S, *et al. BMJ Open* 2022; 12:e054946. doi: 10.1136/bmjopen-2021-054946

Description of the visiting hours and allowable visitors (check if visually displayed and ask an administrator) (please take a photo if there is a sign)

Description of the accessibility to toilets or washrooms

Description of overnight accommodation for family members/friends of women (check if visually displayed and ask an administrator)

Description of the **operating theatres** (how is access to the theatre, is there one theatre reserved for obstetrics, is the theatre on the same floor? How are handwashing facilities)

Please detail how many theatres are exclusively to perform a CS (#)	

BMJ Publishing Group Limited (BMJ) disclaims all liability and responsibility arising from any reliance placed on this supplemental material which has been supplied by the author(s)

Please sketch the physical space

Rungreangkulkij S, *et al. BMJ Open* 2022; 12:e054946. doi: 10.1136/bmjopen-2021-054946

Part 2. Health workforce and model of care.

Completing this section may require both observation of the labour ward and a discussion with staff, e.g.: a matron-in-charge or head of obstetrics.

Please indicate below the **staffing available for the <u>delivery ward</u> alone. Please include all staff on the payroll**. Please do not count the staff only working on ANC or PNC. If staff works part-time in the maternity, try to summarise to have full-time-equivalents.

STAFFING – LABOUR AND DELIVERY WARD ONLY		
	**Staff category**	Number employed (Full-time equivalent)
1.	Obstetrician	
2.	Anaesthesiologists	
3.	Paediatrician	
4.	Medical doctors (graduated, no specialization)	
5.	Residents (medics in training)	
6.	Nurses / nurse-midwives	
7.	Distinct midwives (exclusively trained on midwifery)	
8.	Delivery assistants / auxiliary nursing staff	
9.	Cleaners / other auxiliary non-nursing staff	

STAFFING ON SHIFTS IN LABOUR AND DELIVERY			
	**Staff category**	**DAY SHIFT** Number employed (Full-time equivalent)	**NIGHT SHIFT** Number employed (Full-time equivalent)
1.	Obstetrician		
2.	Anesthesiologists		
3.	Pediatrician		
4.	Medical doctors (graduated, no specialization)		
5.	Residents (medics in training)		
6.	Nurses / nurse-midwives		
7.	Distinct midwives (exclusively trained in midwifery)		
8.	Delivery assistants / auxiliary nursing staff		
9.	Cleaners / other auxiliary non-nursing staff		

Rungreangkulkij S, *et al. BMJ Open* 2022; 12:e054946. doi: 10.1136/bmjopen-2021-054946

Please indicate below the **staffing available for the ANTENATAL CARE ward alone. Please include all staff on the payroll**. Please do not count the staff only working on ANC or PNC. If staff works part-time in the maternity, try to summarise to have Full-time-equivalents.

STAFFING – ANTENATAL CARE WARD ONLY		
	**Staff category**	Number employed (Full-time equivalent)
1.	Obstetrician	
2.	Anesthesiologists	
3.	Pediatrician	
4.	Medical doctors (graduated, no specialization)	
5.	Residents (medics in training)	
6.	Nurses / nurse-midwives	
7.	Distinct midwives (exclusively trained on midwifery)	
8.	Delivery assistants / auxiliary nursing staff	
9.	Cleaners / other auxiliary non-nursing staff	

STAFFING ON SHIFTS IN ANTENATAL CARE WARD			
	**Staff category**	**DAY SHIFT** Number employed (Full-time equivalent)	**NIGHT SHIFT** Number employed (Full-time equivalent)
1.	Obstetrician		
2.	Anesthesiologists		
3.	Pediatrician		
4.	Medical doctors (graduated, no specialization)		
5.	Residents (medics in training)		
6.	Nurses / nurse-midwives		
7.	Distinct midwives (exclusively trained in midwifery)		
8.	Delivery assistants / auxiliary nursing staff		
9.	Cleaners / other auxiliary non-nursing staff		

Rungreangkulkij S, *et al. BMJ Open* 2022; 12:e054946. doi: 10.1136/bmjopen-2021-054946

Please explain the on-call system: Is the doctor to perform a CS in the hospital even at night, or is s/he on-call at home? Are there other resource-persons one can call in if needed?

Part 3. Protocols and guidelines for managing clinical care during labour and childbirth.

Please list the protocols available and in use in this hospital. Please indicate if the head of maternity indicates the presence. If so ask about the type of guidelines (national standard guidelines e.g by MoH or professional organisation) and if the guideline is physically available, e.g in a folder or displayed at the wall.

**S. No**	**List of Protocols**	Present<br>1. Yes<br>2. No	Type<br>1. National Standards<br>2. Hospital Specified	Displayed<br>Yes<br>No
1.	Partograph use / fetal monitoring			
2.	Active Management of Third Stage of labor			
3.	Postpartum haemorrhage management			
4.	Blood transfusion			
5.	Pre-term labor			
6.	Induction / augmentation of labour			
7.	Antenatal steroids			
8.	Obstructed labour			
9.	Previous CS (trial of labour)			

Rungreangkulkij S, *et al. BMJ Open* 2022; 12:e054946. doi: 10.1136/bmjopen-2021-054946

Completing this section may require both observation of the labour ward (e.g.: posters or signs) and a discussion with staff, e.g.: a matron-in-charge or head of obstetrics. If possible, make a photo if there are guidelines on the wall.

Could you describe how any clinical protocols or guidelines for managing *routine or complicated labour and childbirth* care were developed or adapted, and updated? Is there a team in the hospital taking care of this? Can you explain how are these clinical protocols/guidelines used?

Continuous education and quality improvement

Specify for trainings provided in the last 1 year

**List of training provided**	Type<br>1. National Standard training<br>2. Hospital Specified	Was the training provided to<br>1. All providers<br>2. Only doctors<br>3. Only nurse-midwives
Partograph use / fetal monitoring		
Active Management of Third Stage of labour		
Postpartum haemorrhage management		
Blood transfusion		
Pre-term labor /		
Introduction / augmentation of labour		
Antenatal steroids		
Obstructed labour		
Previous CS (trial of labour)		

Could you please describe other quality improvement activities ongoing in this facility. How are nurses and doctors informed about new knowledge and guidelines? Do you need to go regularly to refresh knowledge to workshops or trainings?

Do you do on-the-job training / mentoring of younger colleagues? Is there a system of supervision? Is there a system to discuss difficult cases, e.g. during a morning report? Do you do audits of cases?

Please explain how informed consent is obtained for caesarean section (oral, written, included in medical record, etc).

Part 4. Assessment of facility medical records and data management systems

Please review individual medical records to assess the information currently collected related to key obstetric variables at an *individual level**. Please also review any facility-level register, log book or other records to assess information currently collected related to key obstetric variables at a* *facility level**. It may be helpful to discuss the medical and facility records with the staff, e.g.: a matron-in-charge or head of obstetrics. Collecting this information will help to inform the implementation of the Robson classification system, e.g.: to identify what data is already routinely collected, and what data may need to be added to routine data collection.*

Please explain which information is used. Description of other information routinely collected about caesarean section (e.g. provider, morbidity)

There are typically two different places for documentation a) facility-level register, log book, or other records collating key obstetric variables and b) case notes/patient records. Now first we like to have information on the first type:

Please describe the description of any facility-level register, log book, or other records collating key obstetric variables at the facility-level. Please include whether this register is paper-based or electronic, when it is updated, when and how information is summarised and how often it is reported. Please take a photo (covering patient names).

Who is responsible for completing the facility-level register?

Rungreangkulkij S, *et al. BMJ Open* 2022; 12:e054946. doi: 10.1136/bmjopen-2021-054946

How often is the facility-level register updated and summaries are prepared?

How is the information about facility-level key obstetric variables and outcomes currently integrated into audit and feedback?

Description of the consistency of reporting for these indicators (e.g.: consistently reported across all records reviewed, some data missing – be specific).

Who is present during audit and feedback sessions, and who leads the sessions?

Rungreangkulkij S, *et al. BMJ Open* 2022; 12:e054946. doi: 10.1136/bmjopen-2021-054946

Description of the health facility's "decision-to-incision" time to perform a caesarean section. If not available, please specify.

Now concerning the second type of records: case notes/medical records: Please describe the medical record structure (e.g. electronic or paper), and who keeps the records (e.g. woman or provider)? Is there a standard form? Is the WHO partograph used? Are the data used for audits and feedback?

Is there any regular feedback of these reports to the providers? If so, how often and in what format?

Review of medical records to assess if key obstetric variables needed for Robson classification are correctly and consistently reported <u>*at an individual level.*</u> *For each variable, please (1) ask the administrator how it is reported, and (2) observe a subset of records to assess how variable is actually reported (e.g. 5-10 medical records).*

**Parity**	
**Administrator response**	**Observation of records**

Previous caesarean section	
**Administrator response**	**Observation of records**

Onset of labour (spontaneous, induced, no labour/pre-labour caesarean section)	
**Administrator response**	**Observation of records**

Gestational age (preterm <37 weeks, term ≥ 37 weeks)	
**Administrator response**	**Observation of records**

Fetal presentation or lie (cephalic, breech, transverse)	
**Administrator response**	**Observation of records**

Number of fetuses (singleton, multiple)	
**Administrator response**	**Observation of records**

Who is responsible for completing the individual-level medical records? Does anyone else check for consistent and correct reporting?

Based on the observations and record assessment, what would you consider to be the most appropriate method of implementation of the Robson classification system (manually, using a spreadsheet or automatic calculator, or via electronic records)? Please explain.

[*Ask the administrator*] What type of person would be the most appropriate facility-level champion to implement the Robson classification system, and why? [e.g.: type of provider, what skills this person would have]

[*Ask the administrator*] Who is the best person to *record* data for Robson classification, and why?

[*Ask the administrator*] Who is the best person to *analyse* data for Robson classification, and why?

Rungreangkulkij S, *et al. BMJ Open* 2022; 12:e054946. doi: 10.1136/bmjopen-2021-054946

[*Ask the administrator*] Who is the best person to *report and present* data for Robson classification, and why?

Part 5. Understanding of labour companionship in practice

Completing this section may require both observation of the labour ward and a discussion with staff, e.g.: a matron-in-charge or head of obstetrics. If companionship is not currently allowed at the facility, please specify below.

Description of who is currently allowed to act as a companion for the woman

Description of for what periods of time companionship is offered (e.g.: from admission to discharge, during labour but not childbirth, only at childbirth)

Please detail at what stages / and time of the day companions are allowed:

Labour / first stage	Yes / no
Delivery / second stage	Yes / no
Immediate postpartum period (first hour or two)	Yes / no
Postnatal ward	Yes / no
Day-time	Yes / no
Night-time	Yes / no

Rungreangkulkij S, *et al. BMJ Open* 2022; 12:e054946. doi: 10.1136/bmjopen-2021-054946

Description of the roles that companions usually undertake (e.g.: emotional support, providing food/water/tea to the woman, supporting staff)

Description of how staff currently interact with companions

Existence and content of any orientation materials, protocols, or guidelines related to how staff should work with companions, or on the role of companions. If no materials exist, please state this.

Any other feedback, observations or reflections
