# Supplementary material for: Factors influencing the implementation of labour companionship: formative qualitative research in Thailand
Source: BMJ Open. 2022 May 26;12(5):e054946. doi: 10.1136/bmjopen-2021-054946 (PMC9327797; doi:10.1136/bmjopen-2021-054946)
Supplement: Abstract translation [file bmjopen-2021-054946supp003.pdf]

## Thai abstract

**บทนำ**

องค์การอนามัยโลกมีข้อเสนอแนะว่าผู้หญิงทุกคนควรได้รับโอกาสให้มีทางเลือกที่จะมีเพื่อนช่วยคลอดตลอดระยะเวลาเจ็บครรภ์คลอดจนถึงเด็กคลอดออกมา ทั้ง ๆ ที่มีข้อมูลเกี่ยวกับประโยชน์ของการมีเพื่อนช่วยคลอดอย่างชัดเจน เช่น ประสบการณ์การคลอดที่ดี ลดอัตราการผ่าตัดคลอด แต่พบว่าการมีเพื่อนช่วยคลอดกลับไม่ได้ถูกนำมาใช้อย่างแพร่หลาย สำหรับประเทศไทยการมีเพื่อนช่วยคลอดยังไม่ถูกนำมาใช้กำหนดเป็นนโยบายสำหรับโรงพยาบาลในสังกัดกระทรวงสาธารณสุข การวิจัยนี้มีวัตถุประสงค์ เพื่ออธิบายปัจจัยที่ส่งผลต่อการใช้เพื่อนช่วยคลอดในประเทศไทย

**วิธีการ**

การวิจัยเชิงคุณภาพระยะก่อรูปนี้เพื่อใช้ในการให้ข้อมูล สำหรับ “โครงการวิจัยการตัดสินใจของผู้หญิงและผู้ให้บริการสุขภาพอย่างมีคุณภาพต่อการใช้วิธีการผ่าตัดคลอด” (QUALI-DEC) study เพื่อนำมาใช้ออกแบบ การปรับวิธีการ และการลงปฏิบัติ เพื่อให้การผ่าตัดคลอดมีประโยชน์สูงสุด การศึกษานี้ใช้การเก็บข้อมูลโดยการสัมภาษณ์เชิงลึก การประเมินความพร้อม เพื่อทำความเข้าใจ การรับรู้ของผู้ให้บริการ ผู้หญิง และผู้ที่มีโอกาสเป็นเพื่อนช่วยคลอดเกี่ยวกับการมีเพื่อนช่วยคลอดจากโรงพยาบาลของรัฐ แปดโรงพยาบาล วิเคราะห์ข้อมูลโดยการวิเคราะห์ประเด็น และการพรรณนา วิเคราะห์ปัจจัยที่ส่งผลต่อการนำใช้เพื่อนช่วยคลอดตามกรอบ ความสามารถ โอกาส แรงจูงใจและ และการเปลี่ยนแปลงพฤติกรรม (COM-B)

**ข้อค้นพบ**

ข้อมูลที่ใช้ในการวิเคราะห์ครั้งนี้ได้มาจากการสัมภาษณ์จำนวน 127 คน และ จากแบบประเมินความพร้อมจากแปดโรงพยาบาล ข้อค้นพบจากงานวิจัยเชิงคุณภาพแบ่งออกได้เป็น สี่ประเด็น ดังนี้: ประโยชน์การมีเพื่อนช่วยคลอด บทบาทเพื่อนช่วยคลอด การฝึกอบรมเพื่อนช่วยคลอด และปัจจัยที่ส่งผลต่อการปฏิบัติ ข้อค้นพบแสดงให้เห็นว่า ผู้ให้บริการทางการแพทย์ ผู้หญิงและญาติของพวกเธอ มีทัศนคติทางบวกต่อการมีเพื่อนช่วยคลอด ข้อมูลจากการประเมินความพร้อมและข้อมูลเชิงคุณภาพมีความสอดคล้องกันที่แสดงให้เห็นความท้าทายในการให้มีเพื่อนช่วยคลอดได้ คือ การฝึกอบรมเพื่อนช่วยคลอด ข้อจำกัดพื้นที่ทางกายภาพ การแออัด และนโยบายของโรงพยาบาล

**การอภิปรายผล**

หากเพื่อนช่วยคลอดได้รับการฝึกอบรมอย่างดีเกี่ยวกับ วิธีการสนับสนุนให้กำลังใจผู้หญิง การช่วยลดความปวด และการติดต่อกับผู้ให้บริการ เป็นกิจกรรมที่สามารถนำใช้เพื่อนช่วยคลอดสำหรับโรงพยาบาลในประเทศไทยได้ แต่อุปสรรคในการนำใช้เพื่อนช่วยคลอดจะต้องคำนึงถึงโอกาสที่จะเพิ่มการประสบความสำเร็จที่มีความสัมพันธ์กับการอบรมและพื้นที่ทางกายภาพ ข้อค้นพบนี้ได้ถูกนำเสนอในยุทธศาสตร์ของโครงการ the QUALI-DEC
