# Supplementary material for: Factors influencing the implementation of labour companionship: formative qualitative research in Thailand
Source: BMJ Open. 2022 May 26;12(5):e054946. doi: 10.1136/bmjopen-2021-054946 (PMC9327797; doi:10.1136/bmjopen-2021-054946)
Supplement: Author's manuscript [file bmjopen-2021-054946.draft_revisions.pdf]

BMJ Open is committed to open peer review. As part of this commitment we make the peer review history of every article we publish publicly available.

When an article is published we post the peer reviewers' comments and the authors' responses online. We also post the versions of the paper that were used during peer review. These are the versions that the peer review comments apply to.

The versions of the paper that follow are the versions that were submitted during the peer review process. They are not the versions of record or the final published versions. They should not be cited or distributed as the published version of this manuscript.

BMJ Open is an open access journal and the full, final, typeset and author-corrected version of record of the manuscript is available on our site with no access controls, subscription charges or pay-per-view fees (http://bmjopen.bmj.com).

If you have any questions on BMJ Open's open peer review process please email info.bmjopen@bmj.com

BMJ Open

Factors influencing the implementation of labour companionship: formative qualitative research in Thailand

Journal:	*BMJ Open*
Manuscript ID	bmjopen-2021-054946
Article Type:	Original research
Date Submitted by the Author:	29-Jun-2021
Complete List of Authors:	Rungreangkulkij, Somporn; Khon Kaen University Faculty of Nursing<br>Ratinthorn, Ameporn; Mahidol University Faculty of Nursing<br>Lumbiganon, Pisake; Khon Kaen University Faculty of Medicine, Obstetrics and Gynaecology<br>Zahroh, Rana; The University of Melbourne, Gender and Women's Health Unit, Centre of Health Equity, Melbourne School of Population and Global Health<br>Hanson, Claudia; Karolinska Institute, Public Health Sciences; London School of Hygiene and Tropical Medicine Faculty of Infectious and Tropical Diseases, Dept of Disease Control<br>Dumont, Alexandre; University of Paris, CEPED, Institute for Research on Sustainable Development, IRD-Université de Paris, ERL INSERM SAGESUD<br>de Loenzien, Myriam; Centre Population et Developpement,<br>Betran, Ana Pilar; World Health Organization, 8. UNDP/UNFPA/UNICEF/World Bank Special Program of Research, Development and Research Training in Human Reproduction (HRP), Department of Sexual and Reproductive Health and Research<br>Bohren, Meghan ; University of Melbourne School of Population and Global Health, Gender and Women's Health Unit, Centre for Health Equity
Keywords:	OBSTETRICS, PUBLIC HEALTH, International health services < HEALTH SERVICES ADMINISTRATION & MANAGEMENT, Quality in health care < HEALTH SERVICES ADMINISTRATION & MANAGEMENT, QUALITATIVE RESEARCH

SCHOLARONE™
Manuscripts

I, the Submitting Author has the right to grant and does grant on behalf of all authors of the Work (as defined in the below author licence), an exclusive licence and/or a non-exclusive licence for contributions from authors who are: i) UK Crown employees; ii) where BMJ has agreed a CC-BY licence shall apply, and/or iii) in accordance with the terms applicable for US Federal Government officers or employees acting as part of their official duties; on a worldwide, perpetual, irrevocable, royalty-free basis to BMJ Publishing Group Ltd ("BMJ") its licensees and where the relevant Journal is co-owned by BMJ to the co-owners of the Journal, to publish the Work in this journal and any other BMJ products and to exploit all rights, as set out in our licence.

The Submitting Author accepts and understands that any supply made under these terms is made by BMJ to the Submitting Author unless you are acting as an employee on behalf of your employer or a postgraduate student of an affiliated institution which is paying any applicable article publishing charge ("APC") for Open Access articles. Where the Submitting Author wishes to make the Work available on an Open Access basis (and intends to pay the relevant APC), the terms of reuse of such Open Access shall be governed by a Creative Commons licence – details of these licences and which Creative Commons licence will apply to this Work are set out in our licence referred to above.

Other than as permitted in any relevant BMJ Author's Self Archiving Policies, I confirm this Work has not been accepted for publication elsewhere, is not being considered for publication elsewhere and does not duplicate material already published. I confirm all authors consent to publication of this Work and authorise the granting of this licence.

For peer review only

Factors influencing the implementation of labour companionship: formative qualitative research in Thailand

Authors:

Somporn Rungreangkulkij[1]
Ameporn Ratinthorn[2]
Pisake Lumbiganon[3]
Rana Islamiah Zahroh[4]
Claudia Hanson[5, 6]
Alexandre Dumont[7]
Myriam de Loenzien[7]
Ana Pilar Betrán[8]
Meghan A. Bohren[4*]

* Corresponding author: Meghan A. Bohren meghan.bohren@unimelb.edu.au

Affiliations:

1. Faculty of Nursing, Khon Kaen University, Khon Kaen, Thailand
2. Faculty of Nursing, Mahidol University, Bangkok, Thailand
3. Department of Obstetrics and Gynaecology, Faculty of Medicine, Khon Kaen University, Khon Kaen, Thailand
4. Gender and Women's Health Unit, Centre for Health Equity, School of Population and Global Health, University of Melbourne, Melbourne, Australia
5. Department of Global Public Health, Karolinska Institutet, Stockholm, Sweden
6. London School of Hygiene and Tropical Medicine, London, United Kingdom
7. CEPED, Institute for Research on Sustainable Development, IRD-Université de Paris, ERL INSERM SAGESUD, Paris, France
8. UNDP/UNFPA/UNICEF/World Bank Special Program of Research, Development and Research Training in Human Reproduction (HRP), Department of Sexual and Reproductive Health and Research, World Health Organization, Geneva, Switzerland

For peer review only - http://bmjopen.bmj.com/site/about/guidelines.xhtml

Abstract

Introduction

WHO recommends that all women have the option to have a companion of their choice throughout labour and childbirth. Despite clear benefits of labour companionship, including better birth experiences and reduced caesarean section, labour companionship is not universally implemented. In Thailand, there are no policies for public hospitals to support companionship. This study aims to understand factors affecting implementation of labour companionship in Thailand.

Methods

This is formative qualitative research to inform the "Appropriate use of caesarean section through QUALIty DECision-making by women and providers" (QUALI-DEC) study, to design, adapt and implement a strategy to optimize use of caesarean section. We use in-depth interviews and readiness assessments to explore perceptions of healthcare providers, women, and potential companions about labour companionship in eight Thai public hospitals. Qualitative data were analysed using thematic analysis, and narrative summaries of the readiness assessment were generated. Factors potentially affecting implementation were mapped to the Capability, Opportunity, and Motivation behaviour change model (COM-B).

Results

127 qualitative interviews and eight readiness assessments are included in this analysis. The qualitative findings were grouped in four themes: benefits of labour companions, roles of labour companions, training for labour companions, and factors affecting implementation. The findings showed that healthcare providers, women, and their relatives, all had positive attitudes toward having labour companions. The readiness assessment highlighted implementation challenges related to training the companion, physical space constraints, overcrowding, and facility policies, reiterated by the qualitative reports.

Discussion

If labour companions are well-trained on how to best support women, help them to manage pain, and engage with healthcare teams, it may be a feasible intervention to implement in Thailand. However, key barriers to introducing labour companionship must be addressed to maximise the likelihood of success mainly related to training and space. These findings will be integrated into the QUALI-DEC implementation strategies.

For peer review only - http://bmjopen.bmj.com/site/about/guidelines.xhtml

Strengths and limitations of this study

- Labour companionship has important benefits for the woman and baby, and is recommended by WHO. This is the first study to understand needs and preferences related to labour companionship, and map factors that might affect implementation of labour companionship in Thailand.
- We found that implementation of labour companionship is feasible if labour companions and health workers are well-trained on how to best support women and engage with one another. Addressing key barriers to introducing labour companionship can include changes to the physical environment, implementing facility-level policies on labour companionship, and context-specific solutions to minimise fears on lawsuits and infection.
- A key strength of our study is the triangulation of qualitative research and facility readiness assessments, and mapping of key factors affecting implementation of labour companionship to the COM-B model of behaviour change.
- Using the COM-B model to guide analysis, we show how to use our formative research findings to guide intervention design and support a systematic, targeted, and theory-based development of implementation strategies for labour companionship.
- While our research was conducted in eight public hospitals across different regions of Thailand, the findings may not be transferrable to all settings in Thailand, as most study hospitals were in urban settings with high caesarean section rates.

For peer review only

For peer review only - http://bmjopen.bmj.com/site/about/guidelines.xhtml

Introduction

Efforts to improve maternal health globally have shifted in recent years to improving quality of care. A critical component of quality of care is the person's 'experience of care', which the World Health Organization (WHO) has defined as ensuring that all pregnant people are treated with respect and dignity, have effective communication with health workers, and access to emotional support that meets their needs (1). Within labour and childbirth care, supporting women to have a labour companion of their choice present is an effective way to improve women's experiences by providing respectful care and emotional support (2, 3). Labour companionship refers to a person of the woman's choice, who accompanies the woman continuously throughout labour and childbirth; typically this is the woman's partner or husband, friend or family member (4). Labour companionship empowers women in four key ways (4). Labour companions help to facilitate informational support by helping to communicate between the woman and health workers and helping women with non-pharmacological pain relief (4). Labour companions also act as advocates who speak up in support of the woman, and vocalising her needs and preferences (4). Labour companions provide practical support, such as providing massage, holding the woman's hand and encouraging her to mobilize (4). Lastly, labour companions provide emotional support by being a continuous presence, and praising and reassuring the woman (4).

Labour companionship has important benefits for the woman and baby. A Cochrane intervention review analysed the impact of continuous support for women during labour and childbirth from 26 studies conducted with over 15,000 women in 17 countries, and found that women with continuous support were more likely to have a spontaneous vaginal birth, and less likely to report negative ratings of or feelings about their childbirth experience, or to have a caesarean birth (5). Women with labour companionship also have a shorter duration of labour and better five-minute Apgar scores for their babies. Based on this evidence, WHO recommends that all women have the opportunity to have a labour companion of their choice with them throughout labour and childbirth (3).

Despite clear evidence of benefit, implementation of labour companionship in health facilities across the world remains sub-optimal. A Cochrane qualitative evidence synthesis highlighted several factors affecting implementation, including women and health workers not recognizing the benefits of labour companionship, labour companionship viewed as a 'nice to have' but not essential service, physical space constraints on labour wards and thus difficulties to maintain privacy, and integrating labour companions into part of the care team (4).

For peer review only - http://bmjopen.bmj.com/site/about/guidelines.xhtml

Context of labour companionship in Thailand

In Thailand, labour companions are not typically allowed in most public and some private hospitals. Most public hospitals have a policy allowing women's relative to wait outside the labour room, with certain hours allocated to allow relatives or friends to visit the women in the labour room, typically during lunch or dinner time. Anecdotally, some reasons for not allowing labour companionship were the concern about infection risks (even prior to COVID-19) and maintaining the privacy of women, who normally share rooms, especially from other male companions. With increasing access to mobile phones, there are also emerging concerns about pictures and audio video recordings, which may be used in potential litigation case against medical teams. Similar to the results of the Cochrane review, a quasi-experimental study in eastern Thailand compared the effect of companionship on primiparous women's experiences and found that women with companionship were more satisfied with their childbirth experiences, but no significant difference on self-reported suffering or ability to cope with labour pain was found (6).

The QUALI-DEC Project

In the context of sustained growing caesarean section rates in Thailand, the Ministry of Health and other stakeholders are examining factors underlying the increase and interventions to optimize its use. The QUALI-DEC study: "Appropriate use of caesarean section through **QUALI**ty **DEC**ision-making by women and providers" (7) aims to design, adapt and evaluate a multi-faceted strategy, for the appropriate use of caesarean section in Argentina, Burkina Faso, Thailand and Viet Nam. The QUALI-DEC strategy is designed to combine four key components: 1) Opinion leaders to implement evidence-based clinical guidelines; 2) Caesarean audits and feedback to help providers identify potentially avoidable caesarean sections; 3) A Decision Analysis Tool (DAT) to help women make an informed decision on mode of birth; and 4) Implementation of WHO recommendations on companionship during labour and childbirth (7). The QUALI-DEC strategy for labour companionship supports the woman to choose any person to act as her labour companion. The QUALI-DEC research team and local implementation partners including opinion leaders will co-develop a tailored model for labour companionship in each setting that includes information on 1) changing hospital policy to allow for labour companionship, 2) establishing eligibility criteria for women and companions, 2) identifying how health workers can help women to choose and train the labour companion, 3) defining how health workers engage with women and companions, how many companions are allowed, and when they are present, 5) designing modifications for the physical space to accommodate companions, and 6) developing educational tools for companions on how to support women. Based on the formative research conducted among the local stakeholders in Thailand, the

For peer review only - http://bmjopen.bmj.com/site/about/guidelines.xhtml

aim of this paper is to describe the needs and preferences related to labour companionship, and to map factors that might affect implementation of labour companionship in Thailand, using a behaviour change model.

Methods

This is a formative qualitative study using in-depth interviews (IDIs) and a readiness assessment, described in detail in the study protocol (8) and below. Eight hospitals in Thailand were purposively selected for the QUALI-DEC project according to the willingness to participate, programmatic activities, country priorities, and geographical representation (Table 1). The formative research was conducted in these eight hospitals, where caesarean section rate ranged from 34.3-56.9%.

Participants and recruitment

Five groups of participants were identified for this study: pregnant women, postpartum women, potential companions (before birth), potential companions (after birth), and healthcare providers (doctors, nurse-midwives) and administrators or managers. Pregnant women and postpartum women aged 18 to 49 years who attended antenatal and/or postnatal care at the study hospitals were invited to participate in in-depth interviews (IDIs), aiming for diversity (mix of urban or rural residence, parity, age, and ethnicity - target per facility: 2-3 pregnant and 2-3 postpartum women). Initially, nurse-midwives explored the interest of women during antenatal care or postnatal care visits. If they were potentially interested in participating, then the research team approached women face-to-face. The pregnant and postpartum women who participated in the study identified a person who they would have liked to be their labour companion ("potential companion"), and the research team approached the potential companions face-to-face to participate in an IDI (target per facility: 2-3 potential companions before birth and 2-3 after birth). Typically, the potential companion was already on the hospital grounds. Healthcare providers working on the antenatal, delivery and postnatal wards of the study hospitals and healthcare administrators were contacted by the research team and invited to participate in IDIs, with considerations for a diverse group based on age, gender and years of working experience (target per facility: 2-3 nurse-midwives, 2-3 doctors, 2 administrators). We prespecified the target sample size for each type of participant to account for the variable contexts and patient populations in each facility. No participants approached refused to participate.

For peer review only - http://bmjopen.bmj.com/site/about/guidelines.xhtml

Data collection

After agreeing to participate and completing a consent form, the research team conducted IDIs in Thai at the respective health facility. IDIs lasted 30-90 minutes, had no other people present, were audio-recorded, and participants received 500 Baht (USD$16) compensation for their time. General conversation was initiated prior going to main interview questions to build rapport. Data were collected from July to October 2020. All audio recordings were transcribed verbatim in Thai, complemented with field notes. De-identified transcripts were stored on a password protected computer. There was no further contact with the research participants after the IDI.

The interview guides were developed based on the implementation challenges identified in the Cochrane qualitative review (4) and covered a range of topics including: 1) values and needs around the childbirth period, 2) prenatal education, 3) preferences and decision-making processes regarding mode of birth, and 4) labour companionship (Appendix X: interview guide). Interview guides were piloted and refined prior to data collection. This analysis focuses on the labour companionship module, which included questions such as: Did you want someone to stay with you/women during labour and birth? Why or why not? Who should be the companion? What could their roles be and what are your expectations for them? What type of information or training may they need? What are the potential challenges, opportunities and suggestions related to implementing labour companionship?

In addition to IDIs, a readiness assessment was conducted to describe and assess the service delivery context ahead of the intervention implementation, and was carried out concurrently with the IDIs (Appendix X: readiness assessment). The readiness assessment provides a systematic approach to assessing readiness to engage in the implementation, in order to inform and tailor the interventions in a way best suitable to the local context (8). Readiness assessments were conducted by the research team in each of the study hospitals. During data collection, the researchers used a semi-structured form to observe the service delivery context in each facility setting related to possibility or barriers for companionship implementation such as the sign for visiting information, physical environment in latent room, labour room, and post-partum room (8).

Reflexivity

The QUALI-DEC research team consists of Thai and international social scientists, nurses, doctors, and epidemiologists with maternal health expertise. The research team believed that labour companionship is beneficial for women and families, and may help reduce caesarean section rates.

For peer review only - http://bmjopen.bmj.com/site/about/guidelines.xhtml

The research team was aware of their assumptions and mindful through the study process to mitigate any potentially negative biases that could influence participant responses or interpretations of responses. Six members of research team conducted the IDIs, all were female nursing professors with extensive qualitative experience, no prior relationship with any participants, and did not work at the study sites. Prior to starting data collection, the research team underwent a three-day training on caesarean section globally and in Thailand, QUALI-DEC project, and data collection and management.

Data analysis

Thematic analysis was performed by hand according to the following steps: organizing the data; generating categories, themes, patterns; testing emergent hypothesis; searching for alternative explanations (9). Four members of the research team were involved in the data analysis. Initially, the researchers repeatedly read the interview transcripts to develop initial codes of the data. Secondly, the researchers conducted a systematic identification of themes from the codes such as support, being a representative, and shorten labour. Thirdly, from the themes and codes, researchers identify emerging patterns from the data, such as benefit of having labour companion. Lastly, the researchers review the coded data extracts for each theme to consider whether they appear to form a coherent pattern. If we found inadequacies in the initial coding and themes, we revisit the themes again and changed when they needed. For trustworthiness, during data analysis the findings were discussed among the research team and emergent findings were presented to the representative obstetrician (QUALI-DEC opinion leader) from the study settings. Key themes emerging from the IDIs were combined with data from the Readiness Assessment to identify and prioritize barriers, and to develop potential implications for implementation. Data analysis was conducted in Thai in order to retain the original meaning, and excerpts from the interview transcripts in this article were translated by a bilingual Thai-English translator who is a member of the research team.

The research findings were then conceptualised as factors potentially affecting implementation, and mapped to the Capability, Opportunity, and Motivation model of behaviour change (COM-B) (10). The COM-B model theorises that for a desired behaviour to occur (e.g. labour companionship), individuals must have the **c**apability, **o**pportunity, and **m**otivation to enact the behaviour. Capability refers to factors such as attention, decision-making, knowledge, and skills (10). Opportunity refers to how environments influence behaviour, and includes both physical (e.g. access to supplies and resources, staffing, infrastructure) and social (e.g. team-work, support, practice norms, social and professional identities) contexts (10). Motivation refers to the internal processes that direct and encourage behaviours to occur or not, and includes factors such as perceived benefits, risks and

For peer review only

For peer review only - http://bmjopen.bmj.com/site/about/guidelines.xhtml

consequences, emotions, and priorities (10). The COM-B model has been widely used in implementation research to improve implementation and to explore barriers and facilitators to changing clinical practice. By identifying factors (e.g. barriers and facilitators) that may affect implementation, teams can then design implementation strategies to address these factors and, in turn, optimise the likelihood of successful implementation and potential for scale-up.

Ethical considerations

This research was approved by the Thai Central Research Ethics Committee (CREC) (COA-CREC020/2020), related university research ethics committees, and all hospital research ethics committees. Scientific and technical approval was obtained from the WHO Human Reproduction Programme (HRP) Review Panel on Research Projects (RP2), and ethical approval by the WHO Ethical Review Committee (protocol ID, 004571) and the French Research Institute for Sustainable Development. All participants provided written consent to participate and IDIs were conducted in a private place with no other people present. There was no patient or public involvement in this study.

This paper is reported according to the consolidated criteria for reporting qualitative research (COREQ) guidance (11).

Patient and public involvement

Patients and/or the public were not involved in the design, or conduct, or reporting, or dissemination plans of this research.

Results

From the eight participating hospitals, a total of 127 IDIs are included in this analysis: 27 pregnant women, 25 postpartum women, 16 potential companions, 8 facility administrators, 18 doctors and 33 nurse-midwives working in maternity care. Table 2 presents the sociodemographic characteristics of women and potential companions. Pregnant and postpartum women's ages ranged from 18 to 42 years, almost all were married or cohabitating with a partner, and most were employed. Among pregnant women, about half were nulliparous, including two women who had planned for a caesarean birth. The other half were multiparous. Among postpartum women, at their most recent birth, 32% (8/25) had a vaginal birth, and 68% (17/25) had a caesarean birth. Almost all potential companions identified by the women were their husbands, except one who was the woman's mother.

Table 3 presents the sociodemographic characteristics of healthcare providers. There were 12 men (doctors and administrators) and 59 women (14 female doctors/administrators; all nurse-midwives

For peer review only - http://bmjopen.bmj.com/site/about/guidelines.xhtml

were women). About two-thirds of doctors were working in their professions (12/18) and study facility (11/18) for 10 or fewer years. Compared to doctors, nurse-midwives were working in their profession and study facility for longer (more than 10 years).

Contextual insights from the Readiness Assessment

Observations of the eight hospitals during the readiness assessment demonstrated space limitations and crowding on the labour ward, typically with multiple beds in the same room, close together and only divided by a curtain. Key challenges to introducing labour companionship will thus be overcrowding of the labour ward and maintaining privacy.

There are differences in current visiting hours in the labour and delivery wards across the hospitals. Two hospitals (hospitals 5 and 6) limit the visiting hours to three times a day, 1-2 hours in the morning, noon, and evening. In contrast, the five other hospitals allow visitors from 11 AM- 8 PM, but with limits on the number of visitors and duration of visits. Almost all hospitals allow only one visitor to visit for 15-20 minutes at a time. There is only one hospital (hospital 8) that allows woman who are in labour to visit the relatives at the ward reception area until 8 PM. The restrictions on the timing of visitations and number of visitors allowed may challenge the implementation of labour companionship, and may need to be adjusted prior to implementation to ensure that companions are not subjected to visitor restrictions.

Two hospitals (hospitals 5 and 6) provide onsite overnight accommodation for relatives. One hospital (hospital 2) provides accommodation to the relatives only if the woman in labour is under 20 years old. In addition, two hospitals (hospitals 1 and 8) have a room for the relatives to be with the woman in labour until after the birth for extra charge.

Discussions between the research team and clinical staff as part of the readiness assessment suggested that a potential solution for seven hospitals would be to implement labour companionship for some, but not all women. For example, if seven women are in labour at the same time, labour companionship could be piloted with approximately two or three women without compromising care for all women. In these hospitals, it may be possible to make more private space for women during labour, for example by moving a woman who is in active labour to the corner of the ward and using curtains that are already available. Hospital 5 had serious concerns regarding the seriously limited space that might challenge the implementation of labour companionship.

For peer review only

Qualitative findings related to labour companionship

The findings showed that, in general, healthcare providers, women, and potential companions had positive attitudes about labour companionship. The qualitative findings are grouped in four categories in the subsequent sections: 1) benefits of labour companions, 2) the roles of labour companions, 3) training for labour companions, and 4) factors affecting implementation.

1.0 Benefits of labour companions

Women, companions, and health workers expressed the benefits of having labour companions, including 1) support, warmth and improved marital relationship, 2) having a representative to communicate with the medical team, and 3) perception of clinical benefits. They have noticed the benefits of having the companion included shorten labour duration, to reduce caesarean section, to understand the work of medical team, to reduce the nurse-midwife's workload by being the woman's emotional supporter, and to provide opportunity for professional development. These benefits are outlined in the following sections. Recognition of the benefits of labour companionship are important facilitators for the *reflective* and *automatic motivation* domains of behaviour change, as they refer to the conscious thought processes (plans, evaluations) and habits or desires that influence motivation.

1.1 Support, warmth and improved marital relationship

Many women expressed that they feel anxious during the labour and birth. They feared the labour and birth process in the unfamiliar hospital environment. They experienced pain from contractions and worried about their safety and the baby's health. These women believed that having a companion might reduce fear and anxiety:

> *It is very nice to have some support. Some people need emotional support, wanting to have some familiar faces around. They looked around - they saw only the strangers. If they could see the mom or the husband, they would have felt some support that at least they have a friend. Having companions is very beneficial.*
>
> (Labour nurse-midwife 4, 7-years work experience, hospital 4)

> *It's good to have a companion…have someone to talk to while waiting…I would have felt relaxed…But if I were to have someone with me, I would have felt less anxious and forgot the pain a little bit.*
>
> (Postpartum woman 4, 35 years, hospital 4)

For peer review only - http://bmjopen.bmj.com/site/about/guidelines.xhtml

The participants from all groups said that having a labour companion present during the woman's labour and birth could improve the marital relationship if the husband was chosen to be a companion. The husband and wife could go through the experience of the labour pain, emotional journey together.

> *Having my husband as a companion was very good. It's a very good bonding experience before the baby arrives. It's better for our family relationship.*

(Postpartum woman 10, 23 years, hospital 5)

> *One of the good things about having the companion is that we can support and consult with each other. We can go through it and help each other along the way.*

(Husband 1, 29 years, hospital 8)

1.2 Representative to the medical team

Participants described how labour pain can affect the woman's decision-making, perceptions, and judgment. Therefore, having a companion during labour who was a family member could be useful to act as a representative to communicate with the medical team. This can improve effective communication of the women's needs and preferences.

> *Many times, the patients are in so much pain. We couldn't really communicate with them…They couldn't make sound decisions. If they have a relative who can be their representative, it improves the communication and decision making.*

(Obstetrician 3, 10-years work experience, hospital 4)

> *Having a companion is a good thing. They can be my representative, if something is wrong. They can get a nurse for me.*

(Postpartum woman 4, 35 years, hospital 4)

1.3 Perception of clinical benefits

Some healthcare providers believed that if women had good support, they would be able to manage their pain which, in turn, seem to help shorten the labour duration.

> *One of the good things about having the companion is the smooth delivery…For example if the mom is with the patient, the mother might be able to support because the mother has experienced labour before. They can help the patient to follow the medical team's teaching like how to push correctly. The partner can help guide the patient to a successful labour.*

For peer review only - http://bmjopen.bmj.com/site/about/guidelines.xhtml

(Obstetrician-Administrator 2, 30-years work experience, hospital 4)

Moreover, some healthcare providers believed that when the women had good support, they may manage the pain better than if they did not have a companion. This could result in fewer caesarean births, as some women ask for caesarean because they no longer wish to tolerate the labour pain.

> *Having a companion with the woman seems to help with the surgery request* [for caesarean section]*. When the women are in labour pain, they will have someone with them to distract from the pain…. Many cases they ask for surgery because they are experiencing labour pain and don't want to wait until the natural delivery.*

(Obstetrician 1, 11-years work experience, hospital 7)

1.4 Labour companions at witnesses

When the women's relatives stay with them throughout labour, they can witness the work of medical personals directly. Healthcare workers described that when family members are present, they tended to be more careful while working, which may therefore improve service quality.

> *It is like the companions are the quality assurance inspectors. They see how our system works. It is like a two-way communication that we can improve the quality of our service.*

(Labour nurse-midwife 13, 34-years work experience, hospital 6)

Healthcare workers also felt the presence of companions could reduce some misunderstanding about medical malpractice, as the companion could witness and understand the work of the medical team which may lead to fewer lawsuits.

> *It's beneficial to have a labour companion. If there are any complications during the labour and the delivery, they will see that we try our best. When they see that we are trying the best we can, that might reduce the lawsuits. They have witnessed that we do pay attention. They can participate in the care.*

(Obstetrician 2, 3-years work experience, hospital 7)

1.5 Reduce the nursing workload in emotional support

One of the nursing roles is providing emotional support to women during labour. The nurse-midwives also monitor frequency of contractions and provide other nursing care. When there are many women in labour, the nurse-midwives might not be able to provide close attention to every woman, and emotional support in particular can be compromised. Having a labour companion who

For peer review only

For peer review only - http://bmjopen.bmj.com/site/about/guidelines.xhtml

has been trained on how to support women could therefore potentially reduce the nursing workload.

> *It helps reducing my workload.….I try to pay close attention to all my patients. I can do that when I have only a few patients. But when the patient has a labour companion, I feel good that my patients do receive intensive care, even though it's from the companion, not me.*

(Labour nurse-midwife 14, 10-years work experience, hospital 6)

1.6 Labour companion may not be helpful

Most participants expressed the benefits of having labour companions. However, there were four women who said that they did not need a labour companion, primarily because they believed that during labour, nobody could help alleviate pain. These women believed that during labour, women tended to have limited attention and negative moods.

> *Either way is fine with me, having a companion or not. I am in labour. I will feel pain, no matter I have someone with me or not. Having a companion isn't helping with my pain.*

(Pregnant woman 13, 31 years, hospital 5)

Moreover, one husband also said it was not helpful for him to be there. He said it is better for the woman to be with the medical team, and feared to see her suffer.

> *I think I will not be a labour companion. I will wait outside the room. I don't want to be in the way of the medical team. I am worried but I don't want to see her crying and suffering.*

(Husband 3, 42 years, hospital 3)

2.0 The roles of labour companion

Most healthcare workers said that the women should be the one who select their labour companion. Most women preferred their husbands to be their labour companions, as they think that it will enhance the family relationship, and a few women preferred their mothers as they viewed their mothers' own labour experiences to be beneficial in supporting them. The participants from all groups expressed the roles of the labour companion very similarly, to provide emotional support, massage and support coping with pain, assisting with daily activities, and communicating with the medical team.

For peer review only

I would like someone who can be around and help out. Someone who holds me when I am in pain. Someone who can help getting things for me when I can't really help myself. It is better than being alone.

(Pregnant woman 10, 38 years, hospital 2)

The health workers also perceived that labour companions could play key roles in supporting them to better care for the women in labour.

The first thing is to be my support. Other duties can be understanding the labour and delivery process. So that person isn't in panic. If they notice any unusual symptoms, they can alert the medical team. They should have the ability to observe and report any abnormality. I see this person as a censor who detects problems.

(Obstetrician-Administrator 5, 20-years work experience, hospital 7)

I want to teach and train the companion. They should learn how to assess the labour pain, where they can check or touch. They will be the one who communicates with the nurses that the contraction is more frequent and intense. They can tell the nurses that the patient wants to push already.

(Labour nurse-midwife 15, 5-years work experience, hospital 6)

If labour companions were trained, for example during childbirth education classes or antenatal visits, these health workers believed that they could help the woman to manage pain, and communicate to the health workers if the woman needs help or is ready to push.

These critical roles played by labour companions are important facilitators to the *psychological capability* domain of behaviour change, which can influence the relationship between motivation and enacting the behaviour (labour companionship). If labour companions are appropriately equipped with the skills and knowledge to support women during labour, then they in turn have increased motivation, and health workers may feel better able to integrate them into the care team.

3.0 Training the labour companion

Participants expressed that potential labour companions should receive training to understand the process of labour and how to best support the woman. Preparation of the labour companions could be integrated into the existing antenatal classes.

They have to pass some trainings, including both the woman and the companion. The labour companion should attend some preparation courses. So they know what to do, how to help

For peer review only

For peer review only - http://bmjopen.bmj.com/site/about/guidelines.xhtml

the woman, how to give massage, etc. There has to be a curriculum that prepare the women and the relatives about the labour, what's going to happen while waiting before the delivery, how much the pain, how to prepare, and what to prepare.

(Labour nurse-midwife 3, 11 -years work experience, hospital 4)

Most participants agreed that the training and preparation for the labour companion should start in the third trimester, approximately week 32 of the pregnancy. They should attend the class at least two times, for about 30-60 minutes.

At least they should attend the classes twice. The first time can be when they come to get the lab result after the first or second prepartum visit. But definitely after week 32, the labour companion can attend the ongoing "smart-mom" class. They have to attend at least 2 classes.

(Labour nurse-midwife 16, 16-years work experience, hospital 3)

The key content and skills for labour companions to learn during these sessions is how to provide emotional support, pain management techniques, and understanding the process of labour. One female participant said that the labour companion should understand the emotions while the woman is going through labour pain so they can support the woman appropriately.

The labour companion has to learn how to support the patient. We should teach them what labour is and the pain associate to the labour, how much pain, when to report to the medical team. For instance, if the patient's water broke, they have to let us know. If the patient wants to push, they have to report.

(Obstetrician 2, 3-years work experience, hospital 7)

They have to learn the labour process. It will be somewhat a long process so they can help with the pain while waiting for the delivery. They can be a pushing coach. They have to be perceptive to our moods.

(Postpartum woman 24, 21 years, hospital 1)

The husband of a pregnant woman echoed the desire for learning how to support his wife, and particularly how he could help ease her pain during labour:

I want to learn what I should do, the process of getting on the labour and delivery wards, what to do when I am on the ward, how I can help my wife with the pain.

For peer review only - http://bmjopen.bmj.com/site/about/guidelines.xhtml

(Husband 8, 35 years, hospital 1)

Appropriate training of the labour companion is an important facilitator to the *physical* and *psychological capability* domains of behaviour change, which can increase *motivation*.

4.0 Factors affecting implementation

While all participants noted the many benefits to having a labour companion, some barriers and challenges to implementing companionship were identified. These factors affecting implementation are important barriers and facilitators to *physical* and *social opportunity*, as they relate to creating enabling physical environments and influencing positive sociocultural norms. Many labour and delivery wards in public hospitals are not designed to accommodate labour companions, as the wards are already crowded with women in labour. Consequently, four main barriers were identified by participants: 1) maintaining privacy and confidentiality, 2) increased risk of infection, 3) risk of lawsuits, and 4) perceived additional work for health workers to support companions. Maintaining privacy was already a challenge without labour companions, as the labour ward beds are close together, in a narrow and crowded room. In Thai culture, it is improper for women's bodies to be exposed. Therefore, if a labour companion is a male, it may create discomfort and awkwardness during this sensitive time.

> *Our hospital is a public hospital, not a private one. When the patients in labour, waiting to deliver, they are in their bed with a curtain as a divider between beds. There is no privacy. It's difficult for me to work and to protect my patients' privacy. For example, I am trying to do the pelvic exam but the next bed has a husband accompany her. The voices can travel through. It's difficult to work.*

(Obstetrician 4, 2-years work experience, hospital 4)

In addition to the challenges of physical privacy, some participants also feared that having more visitors and relatives on the ward will be difficult for the medical team to protect the confidential information of patients.

> *I am very afraid of the risk of the confidentiality violation. The companions might talk about other patients to other people. I am very worried about this.*

(Antenatal nurse-midwife 9, 21-years work experience, hospital 3)

Participants, particularly healthcare providers, expressed concerns about increased risks of infection, as the ward is usually crowded with women in labour. Adding the labour companion could lead to the increased risk of infection spread (referring to non-COVID-19 infection).

For peer review only - http://bmjopen.bmj.com/site/about/guidelines.xhtml

I think it's kind of risky for the infection. People wear their normal clothing, not sterile. That might increase the infection spread.

(Labour nurse-midwife 2, 3-years work experience, hospital 7)

Healthcare providers expressed concern that the presence of a labour companion may lead to misunderstanding and lawsuits. They worried that while they are providing care, the companions might think that the medical team are disorganized and in chaos, and that people may post these issues on social media. These misunderstandings and miscommunications had the potential to lead to lawsuits.

When I am on duty, I have to be more careful. My co-workers also warn me about this. For instance, I might be using my smartphone playing on my break but the relatives think I am not helping the patient who are yelling from pain. If they record and pose on social media, people see and misunderstand that I am not doing my job. Having a labour companion is like a two way sword. It has good and bad points.

(Labour nurse-midwife 14, 10-years work experience, hospital 6)

Lastly, many of the study hospitals had high ratios of women to healthcare providers, and healthcare providers feared that introducing companions to the ward may increase their workloads.

The objective of having a labour companion is to have someone to help us. But I doubt that the person can really help me. I have to explain and communicate more. It will double the communication times because I not only communicate with a patient, I have to communicate with the relatives.

(Labour nurse-midwife 6, 3-years work experience, hospital 2)

For successful implementation of companionship, these barriers would need to be considered and addressed in the implementation strategy. However, despite the barriers, the participants, particularly healthcare providers, believed that the potential benefits of introducing labour companionship would outweigh the risks, suggesting that labour companionship was highly acceptable.

I think it's possible to implement the labour companion policy because of the substantial benefits. It is easily acceptable. When there are many evidence-based research that show the benefits of having the labour companion can reduce the active and the second phase of the labour, they will change the policy and practice.

(Obstetrician 8, 3-years work experience, hospital 5)

For peer review only - http://bmjopen.bmj.com/site/about/guidelines.xhtml

For peer review only

If there is a policy to include the labour companion, I think it's possible to follow. They have to provide the space. When the direct order comes to the hospital to do it, they will set up more private space. I think it's possible. There shouldn't be any problems.

(Obstetrician-Administrator 1, 35-years work experience, hospital 7)

Understanding factors affecting implementation using the COM-B model

Figure 1 maps the potential factors affecting implementation from the qualitative interviews and readiness assessment to the COM-B model of behaviour change. The defined behaviour is that all women have the option to have a companion of their choice throughout labour and childbirth. To optimise the likelihood for this behaviour to occur in the QUALI-DEC hospitals in Thailand, the implementation strategies should ensure that the key barriers identified are addressed, and that the facilitators are present and encouraged in all sites.

Discussion

We found that healthcare providers, women, and potential companions in eight public hospitals in Thailand had generally positive attitudes towards having labour companions, and particularly belief that labour companions would provide beneficial psychological and physical support for the women. However, we identified some opportunities and threats in implementing labour companionship for all women. Training the labour companion, for instance through childbirth education classes or attendance at antenatal visits, was important to ensure that the companion knew how to support the woman and understood what to expect during labour and birth. Limited physical space on the labour wards, overcrowding, and multiple beds in the same labour room were major concerns to introducing labour companionship. While policies at the hospital and national level do not currently mention labour companionship, changes are more likely to be made at the hospital-level, and may need to include changes to the visitation policies and where women's families are allowed on the labour ward.

A key facilitator related to the social opportunities is that historically in Thai culture, childbirth occurred at home where the woman was surrounded by her family, and strong values and happiness in welcoming a new family member. Introducing labour companionship for births occurring in health facilities may therefore reflect the values and cultural appropriateness of having a woman's social network supporting her during labour and birth. While there are important barriers to address, namely around policies, training, and reorganisation of the physical environment for birth, social opportunities and psychological capabilities that value companionship are critical which appear to

For peer review only - http://bmjopen.bmj.com/site/about/guidelines.xhtml

be present in Thai culture. These facilitators and barriers are remarkably similar to an implementation study conducted in public hospitals in Egypt, Lebanon, and Syria, where women and families highly valued companion support, but health workers identified critical organisational factors such as limited physical space, lack of training of companions, and limited policy engagement as barriers to successful implementation (12, 13). The implementation study in Egypt, Lebanon, and Syria used participatory engagement through engagement with hospital leaders, seminars with healthcare providers, communications materials for companions, and changes to the physical space (chairs for companions, curtains around beds, access to hot water and toilets, and disposable gowns and nametags for companions) to address these barriers (12), which may also be a useful approach to inform the QUALI-DEC implementation.

Afulani and colleagues similarly explored women and health workers' perceptions of labour companionship in a public maternity unit in rural Kenya, and identified similar facilitators to labour companionship and roles that labour companions could play (14). In contrast to our findings, the Kenyan study identified additional social barriers, including women's belief that companions cannot help them, embarrassment to have a non-health worker see them during labour, and fears that the labour companion would gossip about what they saw during the birth to others or that the labour companion may abuse the woman during labour (14). While we did not identify these social barriers to implementation, it is possible that particularly the embarrassment and fears of gossip and abuse may be present in more rural areas of Thailand (all QUALI-DEC study hospitals are in urban areas and therefore may not be as influenced by these factors present in smaller communities).

Most women and companions believed a partner or husband to be the optimal companion, believing that witnessing the pain and supporting during the difficult time could strengthen the family bonding including the father and the baby, which was consistent with previous studies (4, 15). Only a few women preferred her mother as a companion. This finding is different from other women in India and Bangladesh, most those women wanted their mothers to be a companion (16, 17). Having a female companion, especially a mother, could yield another benefit. This is because mother can share her own experiences on childbirth, which could serve as encouragement to women. We note that cultural and gender norms may influence the choice of a companion, and that ultimately the woman herself should be the person who chooses who will support her.

There are several key implications for research, practice, and implementation of the QUALI-DEC study. We plan to use opinion leaders (influential and respected healthcare leaders who are effective communicators, and identified by their colleagues or local authorities) at each study hospital to help support implementation (7). Engaging with the opinion leaders about the benefits of

For peer review only - http://bmjopen.bmj.com/site/about/guidelines.xhtml

For peer review only

labour companionship and co-designing strategies to address barriers to implementation that are feasible and acceptable in their clinical settings will be critical. We plan to engage with the opinion leaders during an intensive, five-day pre-study training workshop, where we will present the results of this formative research and engage to design strategies to optimise implementation (7). We expect that at a minimum, some reorganisation of the physical space of the labour ward will be needed, for example introducing chairs and supplying curtains where necessary. Likewise, some facility policies may need to be adjusted to change restrictions on visiting hours for the labour ward to ensure that companions are not subjected to visitor restrictions. More work will be needed to explore how to engage with labour companions during the antenatal period, and information, education, and communications materials will be developed to communicate how companions can support women and how health workers can engage them in care.

Our study had both limitations and strengths. While we aimed to include diverse public hospitals across different regions of Thailand, the findings may not be transferable to all settings in Thailand, including Southern Thailand where we could not include any hospitals. All study hospitals were in urban settings and generally hospitals with relatively high caesarean section rates, so there may be limited transferability to rural settings or settings with lower caesarean section rates. We collected the data during the COVID-19 pandemic, which may have introduced additional barriers to implementation around people's presence on the labour wards (during the data collection period July to October 2020, there were typically less than 10 COVID-19 cases per day in Thailand). We note that WHO COVID-19 clinical management guidance recommends that during the pandemic, all women should have access to woman-centred, respectful care, including a companion of their choice; this includes women with suspected, probably, or confirmed COVID-19 (18). Key strengths of our study include triangulation of results from qualitative research and the facility readiness assessment, and mapping of key factors affecting implementation to the COM-B model to guide decision-making during QUALI-DEC intervention design and support a systematic, targeted, and theory-based development of implementation strategies.

Conclusion

Labour companionship is viewed by women, potential companions, and health workers as highly beneficial and acceptable in the Thai context. If labour companions are well-trained on how to best support women, help them to manage pain, and engage with healthcare teams, it may be a feasible intervention to implement in the study hospitals. However, key barriers to introducing labour companionship must be addressed to maximise the likelihood of success. This includes changes to the physical environment in the labour ward to ensure that privacy can be adequately maintained

For peer review only - http://bmjopen.bmj.com/site/about/guidelines.xhtml

and that there is space for companions to comfortably support women. Facility-level policies may need adjustment, particularly around visitation hours and where companions are not restricted. Context-specific solutions may need to be developed to assuage health worker concerns about potential misunderstandings, lawsuits, or reputational risks stemming from the introduction of labour companionship. Health workers will also need training to understand how to engage with labour companionships as part of a woman's care team, to minimise the risk of role encroachment and understand how companionship can be mutually beneficial. These key findings will be considered and deliberated on when developing the QUALI-DEC implementation strategies for introducing labour companionship.

For peer review only

For peer review only - http://bmjopen.bmj.com/site/about/guidelines.xhtml

Acknowledgements

We thank the woman, potential companions, and health workers for their valuable time, and are grateful to the social scientist team for their hard work in data collection: Nilubon Rujiraprasert, Sasitara Nuampa, Natthapat Buaboon, Waraluk Kittiwatanapaisan, and Dasavanh Bounmany.

Funding statement

This study is part of the QUALI-DEC-project which is co-funded by the European Union's Horizon 2020 research and innovation programme under grant agreement No 847567, and by UNDP-UNFPA-UNICEF-WHO-World Bank Special Programme of Research, Development and Research Training in Human Reproduction (HRP), a cosponsored programme executed by the World Health Organization (WHO) in the Department of Sexual and Reproductive Health and Research (SRH). The contents of this article are solely the responsibility of the authors and do not reflect the views of the EU, UNDP, UNFPA, UNICEF, WHO or the World Bank.

Contributorship statement

SR, AR, PL, CH, AD, MdL, APB and MAB designed the study. SR, AR, and PL led data collection with support from CH, AD, MdL, APB and MAB. SR led data analysis with support from RZ and MAB. SR and MAB drafted the manuscript, and all authors reviewed the manuscript.

Data sharing statement

No additional data available.

Patient and public involvement

Patients and/or the public were not involved in the design, or conduct, or reporting, or dissemination plans of this research.

For peer review only - http://bmjopen.bmj.com/site/about/guidelines.xhtml

References

1. Tunçalp Ö, Were WM, MacLennan C, Oladapo OT, Gülmezoglu AM, Bahl R, et al. Quality of care for pregnant women and newborns-the WHO vision. Bjog. 2015;122(8):1045-9.
2. World Health Organization. Companion of choice during labour and childbirth for improved quality of care. World Health Organization; 2016 2016.
3. World Health Organization. WHO recommendations: intrapartum care for a positive childbirth experience. World Health Organization; 2018 2018. Report No.: 978-92-4-155021-5.
4. Bohren MA, Berger BO, Munthe-Kaas H, Tunçalp Ö. Perceptions and experiences of labour companionship: a qualitative evidence synthesis. The Cochrane database of systematic reviews. 2019;3(3):Cd012449.
5. Bohren MA, Hofmeyr GJ, Sakala C, Fukuzawa RK, Cuthbert A. Continuous support for women during childbirth. The Cochrane database of systematic reviews. 2017;7(7):Cd003766.
6. Jaroensook C. Effects of Labor Support by a Female Relative on Suffering from Labor Pain, Coping with Labor Pain, and Satisfaction with Childbirth Experiences Among Primiparous Parturients. The Journal of Faculty of Nursing Burapha University. 2019;26(2):67-75.
7. Dumont A, Betrán AP, Kaboré C, de Loenzien M, Lumbiganon P, Bohren MA, et al. Implementation and evaluation of nonclinical interventions for appropriate use of cesarean section in low- and middle-income countries: protocol for a multisite hybrid effectiveness-implementation type III trial. Implementation Science. 2020;15(1):72.
8. Bohren MA, Opiyo N, Kingdon C, Downe S, Betrán AP. Optimising the use of caesarean section: a generic formative research protocol for implementation preparation. Reproductive health. 2019;16(1):170.
9. Braun V, Clarke V. Using thematic analysis in psychology. Qualitative Research in Psychology. 2006;3(2):77-101.
10. Michie S, Van Stralen MM, West R. The behaviour change wheel: a new method for characterising and designing behaviour change interventions. Implementation science. 2011;6(1):42.
11. Tong A, Sainsbury P, Craig J. Consolidated criteria for reporting qualitative research (COREQ): a 32-item checklist for interviews and focus groups. International Journal for Quality in Health Care. 2007;19(6):349-57.
12. Kabakian-Khasholian T, Bashour H, El-Nemer A, Kharouf M, Elsheikh O, the Labour Companionship Study G. Implementation of a labour companionship model in three public hospitals in Arab middle-income countries. Acta Paediatrica. 2018;107(S471):35-43.
13. Kabakian-Khasholian T, El-Nemer A, Bashour H. Perceptions about labor companionship at public teaching hospitals in three Arab countries. International journal of gynaecology and obstetrics: the official organ of the International Federation of Gynaecology and Obstetrics. 2015;129(3):223-6.
14. Afulani P, Kusi C, Kirumbi L, Walker D. Companionship during facility-based childbirth: results from a mixed-methods study with recently delivered women and providers in Kenya. BMC pregnancy and childbirth. 2018;18(1):150-.
15. Kabakian-Khasholian T, Portela A. Companion of choice at birth: factors affecting implementation. BMC Pregnancy and Childbirth. 2017;17(1):265.
16. Bangal V, Bayaskar V, Arjun A, Khan I, Thorat U. Opinions of pregnant women regarding desire and choice of labour companion. Women's Health. 2018;7.
17. Perkins J, Rahman AE, Mhajabin S, Siddique AB, Mazumder T, Haider MR, et al. Humanised childbirth: the status of emotional support of women in rural Bangladesh. Sexual and Reproductive Health Matters. 2019;27(1):228-47.
18. World Health Organization. Clinical management of COVID-19 patients: Living guidance. Geneva, Switzerland: World Health Organization; 2021.

For peer review only - http://bmjopen.bmj.com/site/about/guidelines.xhtml

Figures and Tables

Table 1. Study sites in Thailand

Hospital #	Region	Type of hospital	# of births per year (2020)	Caesarean section rate (2020)
Hospital 1	Central Thailand	Public hospital	4,431	43.6%
Hospital 2	Central Thailand	Public hospital	4,605	34.3%
Hospital 3	Central Thailand	Public teaching hospital	5,203	48.5%
Hospital 4	Northeast Thailand	Public teaching hospital	1,727	42.5%
Hospital 5	Northeast Thailand	Public hospital	4,756	43.6%
Hospital 6	Northeast Thailand	Public hospital	3,361	49.2%
Hospital 7	Northern Thailand	Public hospital	5,025	50.1%
Hospital 8	Eastern Thailand	Public hospital	3,268	56.9%

For peer review only

For peer review only - http://bmjopen.bmj.com/site/about/guidelines.xhtml

Table 2. Sociodemographic of participants: women and potential companions

	Pregnant women	Postpartum women	Potential companions
**Total number of participants**	27	25	16
**Age (years)**			
18-24	8	4	0
25-30	9	9	4
31-42	10	12	10
43-59	0	0	2
**Marital status**			
Single	0	0	0
Married/cohabitating	26	25	15
Divorced/widowed	1	0	1
**Occupation**			
Government officer	3	2	0
Business owner	8	5	5
Employed (other)	8	11	10
Unemployed	8	7	1
**Parity and planned mode of birth**			
Nulliparous (no planned CS)	10	-	-
Nulliparous (planned CS)	2	-	-
Multiparous (no planned CS)	9	-	-
Multiparous (planned CS)	6	-	-
**Mode of birth (most recent birth)**			
Vaginal birth	-	8	-
CS	-	17	-

CS: caesarean section

For peer review only

Table 3. Sociodemographic of participants: health care providers

	Administrators	Doctors	Nurse-midwives
**Total number of participants**	8	18	33
**Gender**			
Female	2	12	33
Male	6	6	0
**Years working in total**			
1-5	0	7	8
6-10	0	5	2
11-15	0	2	1
16-20	1	2	5
21-25	0	1	5
26-30	4	1	4
≥ 31	3	0	8
**Years working at study facility**			
1-5	0	11	10
6-10	0	1	5
11-15	0	3	4
16-20	1	2	4
21-25	0	0	4
26-30	4	1	3
≥ 31	3	0	3

For peer review only

For peer review only - http://bmjopen.bmj.com/site/about/guidelines.xhtml

Figure 1. Mapping the factors affecting implementation of labour companionship in Thailand to the COM-B model of behaviour change. This figure maps the factors affecting labour companionship from the qualitative research findings and readiness assessment to the COM-B model of behaviour change. The COM-B model is a useful way to identify what changes need to occur for an intervention – such as companionship – to be effective. Developing implementation strategies that capitalise on the facilitators and address the barriers to capability, opportunity, and motivation is a critical next step for the QUALI-DEC project.

[insert figure 1 here]

Footnote to figure 1:

Data coming from: * = women, ˠ = labour companion, † = doctors, ‡ = nurse-midwives, R = readiness assessment

For peer review only

For peer review only - http://bmjopen.bmj.com/site/about/guidelines.xhtml

Capability to have a labour companion

Physical

Facilitators (*for labour companionship to be implemented*):

Labour companions should have sufficient time to assist woman throughout labour*
Labour companions should be able to join parenting classes or antenatal visits ‡
Labour companions should receive training on how to support women during labour and birth in parenting class or antenatal visit*¥†‡
Some facilities have piloted labour companionship successfully†‡

Barriers:

No national policies include labour companionship†‡
Inability to maintain privacy of women†‡

Psychological

Facilitators (*for labour companionship to be implemented*):

Labour companion, women and health workers should be aware the benefits of labour companionship*¥†‡
Women and health workers should be aware that women can have someone to support them during labour*†‡
Labour companions should understand labour process*†‡
Labour companions should understand women's pain and how to support them*
Labour companions should know how to massage and other measures to support women to cope with pain*¥
Labour companions should can provide emotional support to women*¥
Labour companions should know how to assist with daily activities while at the facility*
Labour companions should know how to and are comfortable to communicate with medical team*
Labour companions should **have previous experience** of labour or labour companionship*
Labour companions should have a family relation of the woman, most preferred husband or mother*
Labour companions should help ease health worker workload by providing emotional support to women†‡
Labour companions should can help identify when women need additional help from health workers†‡

Barriers:

Labour companion **has no previous experience** in labour or labour companionship*¥

Motivation to have a labour companion

Reflective

Facilitators:

Participants beliefs that labour companion will:

Have positive clinical effects: shorten labour duration, reduction of caesarean section, professional development†‡
Improve understanding between family and medical team†‡
Reduce nurses' workload in providing emotional support to women‡
Represent and communicate women's needs*¥†‡
Improve pain management of women†‡
Be a quality assurance inspector†‡

Barriers:

Fear of increased infection risk from labour companion's presence on labour ward*†‡
Fear of misunderstandings and lawsuits†‡
Fear of increased workload for health workers†‡

Automatic

Facilitators:

Belief of positive benefits of labour companionship*¥†‡
Belief that labour companion will reduce women's stress, loneliness and pain during labour*¥†‡
Belief that labour companion's presence will strengthen marital relationship*¥†‡
Women feel supported, relaxed and warmth*¥†‡

Barriers:

Labour companion fear of seeing women suffering¥
Perception that labour companion is not helpful*¥

Opportunity to have a labour companion

Physical

Facilitators:

When labour occurs in private room with no others present[R]*¥†‡
Some facilities have 'premium' suites that allow for labour companionship[R]†‡
Labour space management for labour to ensure privacy, i.e. corner of the room[R]‡

Barriers:

Space limitations in labour ward*[R]†‡
Overcrowding of labour ward*[R]†‡
Privacy in labour ward is by curtains between beds which may be insufficient for maintaining privacy†‡
No accommodation available for labour companion[R]
Current restrictions on timing and number of visitations[R]
Potential labour companions may not have time availability to participate in training as a companion - may not know how to best support women[R]*¥

Social

Facilitators:

Thai culture promotes family support during labour [R]
Opportunity for local action and planning to implement labour companionship[R]†‡

Barriers:

May not be appropriate for women to have men who are not health workers see their bodies*†‡
No national policies support labour companionship†‡

Behaviour: *All women have the option to have a companion of their choice throughout labour and childbirth*

Appendix 1. In-depth interview guides

This file contains the in-depth interview guides for all participant groups in this study:

1. Pregnant women (page 2)
2. Postpartum women (page 6)
3. Potential companions before birth (page 11)
4. Potential companions after birth (page 15)
5. Health workers (page 19)

For peer review only

In-depth interview: pregnant women

Step 1: Introduce yourself to the participant. Describe the purpose of the interview and how information will be used. Obtain oral/written consent.
Step 2: Ask the participant to identify herself. Interviewer: fill out the information below prior to beginning the interview.
Step 3: Conduct the interview. Please remember to audio record the interview.

Interview place: ______________________________

Interview date: _______________________________

Start time: __________________________________

End time: ___________________________________

Interviewer name: ______________________________

Interview identification: _________________________________

<u>Participant information</u>

Duration of current pregnancy (weeks/months, please label):______________________________

Age (years): ____

Marital status (Single, married/cohabitating, divorced, widowed): ____________________

Occupation (write in): ____

For peer review only

For peer review only - http://bmjopen.bmj.com/site/about/guidelines.xhtml

Part 1. Values and needs surrounding the childbirth period

1. What are some things you are doing to prepare for your birth?
    a. Probe: who is helping you to prepare? How are they helping?

2. Thinking about your pregnancy, what are some of the things you value most? Why?
    a. Probe: to keep yourself healthy?
    b. Probe: to keep your baby healthy?

3. Thinking to the future about your birth, what are some of the things that are most important to you?
    a. Probe: What will you need from your doctors and midwives to make sure this happens?
    b. Probe: what will you need from your family to make sure this happens?
    c. Where are you planning to give birth? Why?
        i. Did anyone help you decide where to give birth? Who? How did they help?

Part 2. Prenatal education

4. Thinking about when you go to your antenatal care visits, what are some of the things that are most important to you to learn about?
    a. Probe: What are some of the most important things you have learned during antenatal care? Why are they important?
    b. Probe: How do you think antenatal care could be improved?
        i. What do you think is missing from your antenatal care visits?
        ii. Are there any things that you would remove or change during your antenatal care visits?

Part 3. Preferences and decision-making processes regarding mode of childbirth

5. Could you tell me about the different ways that women can give birth?
    a. Probe: How did you learn about these options?

6. What do you think about vaginal birth and caesarean section?

7. What do you think are some of the positive things about vaginal birth?
    a. Probe: Why are these positive things?
    b. Probe: How did you learn about these positive things?

8. What are some of the negative things about vaginal birth?
    a. Probe: Why are these negative things?
    b. Probe: How did you learn about these negative things?

9. What do you think are some of the positive things about caesarean section?
    a. Probe: Why are these positive things?
    b. Probe: How did you learn about these positive things?

10. What are some of the negative things about caesarean section?
    a. Probe: Why are these negative things?
    b. Probe: How did you learn about these negative things?

11. How did you learn about vaginal birth and caesarean section?

For peer review only - http://bmjopen.bmj.com/site/about/guidelines.xhtml

12. How would you prefer to give birth? For example, caesarean or vaginal birth?
    a. Why do you prefer to give birth this way?
    b. How important is it for you to give birth this way? Why?
    c. Was anyone involved in helping you make a decision about how you prefer to give birth?
        i. Probe: Will your (husband/partner) influence this decision? How?
        ii. Probe: Will your family influence this decision? How?
        iii. Probe: Will your friends influence this decision? How?
        iv. Probe: Will your doctor or midwife influence this decision? How?
        v. Probe: Will the media influence this decision? How?

13. Do you feel like you have enough information to understand the options that you have for how to give birth? Why or why not?
    a. Probe: What other type of information about different modes of childbirth would you be interested to learn about?
    b. At what point during your pregnancy would you like to receive this information?

14. Did you discuss your preference with your doctor or midwife? If so, what was the discussion like?

15. *A decision-analysis tool could help to educate women about their options for mode of birth and how to discuss their preferences with a doctor. Interviewer: show the woman the Vietnam decision analysis tool.*
    a. Would this type of tool be helpful to you? Why or why not?
    b. What type of information would you like to have included?
    c. *This type of decision tool can be paper based like this example, or could be an application for a phone*. Which of these options do you prefer and why?

16. Pregnancy and childbirth are exciting times but can also be scary for some women. Is there anything that you are afraid or nervous of during your pregnancy? Why or why not?
    Note to interviewer: if they bring up fear of pain, then probe about what pain management technique they have learned about.

    a. What about during your birth, is there anything that you are afraid of? Why or why not?
        i. Have you spoken to your doctor or midwife about these fears? Why or why not? What did they tell you?
        ii. Have you spoken to anyone else about these fears?
            1. *If yes*:
                a. Who did you speak to? Why did you choose to speak to this person?
                b. What type of advice did they give you?
            2. *If no*: Do you plan to speak to anyone about these fears? Why or why not?
        iii. What do you think could be done to help reduce this fear for you?

Part 4. Labour companionship

17. What do you need in order to have a positive experience when you go to the hospital for childbirth?
    d. What type of support do you think you need during labour and childbirth?

For peer review only - http://bmjopen.bmj.com/site/about/guidelines.xhtml

Interviewer read: A labour companion is a person of the woman's choice, who can help to provide emotional support to the woman during labour and childbirth. Typically, this person would be with the woman continuously throughout labour and childbirth. This person may be the woman's husband/partner, her mother, or a friend.

18. Do you think you will receive this type of support? Why or why not?

19. <u>Have you ever heard of someone providing this type of support?</u>

20. <u>What do you think of this type of support?</u>

21. <u>Do you know if labour companionship is allowed in the hospital you plan to give birth in?</u>
    - e. *If labour companionship is not allowed*: What do you think are the reasons for not allowing a labour companion?
    - f. Would you be allowed a labour companion if you requested it? Why or why not?
    - g. In your opinion, what changes do you think the hospital could make in order to make it more comfortable for women to have a labour companion?

22. <u>Do you think you would want to have a labour companion for your upcoming birth? Why or why not?</u>
    - h. What type of information or education would YOU need before deciding if you wanted to have a labour companion to support you?

23. <u>If you were to have a labour companion with you:</u>
    - i. What would you expect from this person?
    - j. When would you want this person to be with you in the hospital (probe: the whole time, only during labour but not during the birth, something else?)
    - k. Who would you prefer this person to be? Why?
    - l. When would you like to start talking to your labour companion about their role during your labour and childbirth?
  - iii. Probe: at what month during your pregnancy?

24. <u>What type of information or education do you think a labour companion would need to be able to support you?</u>

Thank you so much for your time. Is there anything else that you would like to share with me today about anything we talked about?

For peer review only - http://bmjopen.bmj.com/site/about/guidelines.xhtml

In-depth interview: postpartum women

Step 1: Introduce yourself to the participant. Describe the purpose of the interview and how information will be used. Obtain oral/written consent.
Step 2: Ask the participant to identify herself. Interviewer: fill out the information below prior to beginning the interview.
Step 3: Conduct the interview. Please remember to audio record the interview.

Interview place: ________________________________

Interview date: _________________________________

Start time: ____________________________________

End time: _____________________________________

Interviewer name: _________________________________

Interview identification: ___________________________________

<u>Participant information</u>

Duration of current pregnancy (weeks/months, please label):________________________________

Age (years): ____

Marital status (Single, married/cohabitating, divorced, widowed): _____________________

Occupation (write in): ____

For peer review only - http://bmjopen.bmj.com/site/about/guidelines.xhtml
For peer review only

Part 1. Values and needs surrounding the childbirth period

25. Can you tell me about your recent birth?
    a. Where did you give birth?
    b. Were you planning to give birth there? Why or why not?
    c. How did you make the decision about where to give birth?
        i. Who was involved in the decision about where to give birth?
        ii. Probe: Did you influence this decision? How?
        iii. Probe: Did your family or her family influence this decision? How?
        iv. Probe: Did your friends or her friends influence this decision? How?
        v. Probe: Did the doctor or midwife influence this decision? How?
        vi. Probe: Did the media influence this decision? How?

26. <u>How did you feel about your overall experience of giving birth?</u>
    a. How would you describe your birth experience? Why?

27. <u>Satisfaction</u>
    a. How satisfied are you with the type of care you received during labour and childbirth?
    b. Can you give me an example of something you are very satisfied with?
    c. Can you give me an example of something you are NOT satisfied with?

28. <u>How well do you feel that your healthcare provider respected your opinions about care during labour and childbirth?</u>
    a. Can you give me an example of a time when your healthcare provider respected your opinions about your care during labour and childbirth?
        i. How did this make you feel?
    b. Can you give me an example of a time when your healthcare provider did NOT respect your opinions about your care during labour and childbirth?
        i. Probe: Or, an example of a time during labour and childbirth when someone else was making decision without talking with you?
            1. How did this make you feel?

Part 2. Decision-making processes regarding mode of childbirth

29. <u>Could you tell me about the different ways that women can give birth?</u>
    a. Probe: How did you learn about these options?

30. <u>What about for your most recent birth – how did you give birth? For example, caesarean or vaginal birth?</u>
    a. Is this the way that you preferred to give birth? Why or why not?
    b. Probe: How did you come to give birth in this way?
        i. Probe: Did your (husband/partner) influence this decision? How?
        ii. Probe: Did your family influence this decision? How?
        iii. Probe: Did your friends influence this decision? How?
        iv. Probe: Did your doctor or midwife influence this decision? How?
            1. Did you discuss this decision with your doctor or midwife? If so, what was the discussion like?
        v. Probe: Did the media influence this decision? How?

31. <u>What do you think about vaginal birth and caesarean section?</u>
    a. What do you think are some of the positive things about vaginal birth?

For peer review only - http://bmjopen.bmj.com/site/about/guidelines.xhtml

i. Probe: Why are these positive things?
ii. Probe: How did you learn about these positive things?

b. What are some of the negative things about vaginal birth?
   i. Probe: Why are these positive things?
   ii. Probe: How did you learn about these positive things?
c. What do you think are some of the positive things about caesarean section?
   i. Probe: Why are these positive things?
   ii. Probe: How did you learn about these positive things?
d. What are some of the negative things about caesarean section?
   i. Probe: Why are these positive things?
   ii. Probe: How did you learn about these positive things?

32. <u>How did you learn about vaginal birth and caesarean section?</u>

33. <u>Did you feel like you had enough information to understand the options that you had for how to give birth? Why or why not?</u>
    a. Probe: What other type of information about different modes of childbirth would you have been interested to learn about?
    b. At what point during your pregnancy would you like to receive this information?

Part 3. Prenatal education

34. <u>Thinking back to your antenatal care visits, what were some of the things that were most important to you to learn about?</u>
    a. Probe: What are some of the most important things you have learned during antenatal care? Why are they important?
    b. Probe: How do you think antenatal care could be improved?
       i. What do you think was missing from your antenatal care visits?
       ii. Are there any things that you would remove or change during your antenatal care visits?

Part 4. Decision-aids

35. <u>Where did you get most of the information to educate you about what to expect while giving birth?</u>
    a. What type of topics did you learn about?
    b. How well do you feel these educational materials prepared you to give birth? Why?
       i. Probe: can you give me an example of something that you felt very well prepared for?
       ii. Probe: can you give me an example of something that you did NOT feel well prepared for?
    c. Overall, how well prepared did you feel to give birth? Why?

For peer review only - http://bmjopen.bmj.com/site/about/guidelines.xhtml

36. Did you feel like you had sufficient time to talk to your doctor or midwife about any concerns that you had about labour and childbirth? Why or why not?
    a. Can you give me an example of a time when you felt that you were able to discuss your questions or concerns with your doctor or midwife?
    b. Can you give me an example of a time when you felt that you were NOT able to discuss your questions or concerns with your doctor or midwife?

37. *A decision-analysis tool could help to educate women about their options for mode of birth and how to discuss their preferences with a doctor.* Interviewer: show the woman the Vietnam decision analysis tool.
    a. Would this type of tool be helpful to you? Why or why not?
    b. What type of information would you like to have included?
    c. This type of decision tool can be paper based like this example, or could be an application for a phone. Which of these options do you prefer and why?

Part 5. Labour companionship

38. Who was with you while you were in labour?
    a. Probe: was your husband/partner with you?
        i. *If yes*:
            1. What was he doing while you were in labour and giving birth?
            2. Was he in the room with you? Why or why not?

    b. Probe: were any of your family members or friends with you?
        i. *If yes*:
            1. Who was with you?
            2. What were they doing while you were in labour and giving birth?
            3. Were they in the room with you? Why or why not?

39. What type of support do you think that you need during labour and childbirth while in the facility?
    a. Did you feel that you were supported during labour and childbirth? Why or why not?
    b. Can you give me an example of when you did feel supported?
    c. Can you give me an example of when you did not feel supported?
    d. What could have been done to improve your experience of support during labour and childbirth?
        i. Probe: Why do you think this is important?

Interviewer to read: Some women have a person with them during labour and childbirth, and we call this person a "labour companion". A labour companion is typically a woman's husband, boyfriend, sister, mother, or friend, who stays with the woman throughout labour and childbirth. They help the woman by providing emotional support, praising her and reassuring her.

40. What do you think about this type of support?

41. Would you have wanted someone to support you in this way during your labour and childbirth? Why or why not?

For peer review only - http://bmjopen.bmj.com/site/about/guidelines.xhtml

42. Who would you want this person to be?
    a. Probe: your husband/partner? Why?
    b. Probe: a sister or friend? Why?
    c. Probe: a mother or mother-in-law? Why?

43. When would you want to have this person with you?
    a. Probe: all of the time during labour and childbirth?
    b. Probe: only some of the time (e.g. only during labour, but not the birth)

44. How do you think having a labour companion might be helpful?

45. What are some challenges to having a labour companion?

46. Do you know if labour companionship is allowed in the hospital you gave birth in?
    a. *If labour companionship is not allowed:* What do you think are the reasons for not allowing a labour companion in this hospital?

47. What changes do you think the hospital could make to make it more comfortable for women to have a labour companion?

48. Do you have any other comments or feedback about labour companionship?

Thank you so much for your time. Is there anything else that you would like to share with me today about anything we talked about?

For peer review only

For peer review only - http://bmjopen.bmj.com/site/about/guidelines.xhtml

In-depth interview: partner / potential companion (before birth)

Step 1: Introduce yourself to the participant. Describe the purpose of the interview and how information will be used. Obtain oral/written consent.
Step 2: Ask the participant to identify herself. Interviewer: fill out the information below prior to beginning the interview.
Step 3: Conduct the interview. Please remember to audio record the interview.

Interview place: ______________________________

Interview date: ______________________________

Start time: ______________________________

End time: ______________________________

Interviewer name: ______________________________

Interview identification: ______________________________

<u>Participant information</u>

Relationship with pregnant woman: ______________________________

Age (years): ______________________________

Marital status (Single, married/cohabitating, divorced, widowed): ____________________

Occupation (write in): ______________________________

<u>Information on pregnant women</u>

N° of women's interview: ______________________________

Duration of current pregnancy:______________________________

For peer review only - http://bmjopen.bmj.com/site/about/guidelines.xhtml
For peer review only

Part 1. Values and needs surrounding the childbirth period

49. What are some things you are doing to prepare for your (wife/partner/daughter/sister…) birth?
    a. Probe: who is helping you to prepare? How are they helping?

50. Thinking about your wife/partner/sister pregnancy, what are some of the things you value most? Why?
    a. Probe: to keep her healthy?
    b. Probe: to keep the baby healthy?
    c. Probe: for yourself personally?

51. Thinking to the future about your (wife/partner/daughter/sister…) birth, what are some of the things that are most important to you?
    a. Probe: What will you need from your doctors and midwives to make sure this happens?
    b. Probe: what will you need from your family to make sure this happens?

52. Place of birth
    a. Where is your (wife/partner/daughter/sister…) planning to give birth? Why?
    b. How did she make the decision about where to give birth?
        i. Did anyone help her make the decision about where to give birth? Who? How?

Part 2. Decision-making processes regarding mode of childbirth

53. Could you tell me about the different ways that women can give birth?
    a. Probe: How did you learn about these options?

54. What do you think about vaginal birth and caesarean section?
    a. What do you think are some of the positive things about vaginal birth?
    b. What are some of the negative things about vaginal birth?
    c. What do you think are some of the positive things about caesarean section?
    d. What are some of the negative things about caesarean section?

55. How would you prefer that your (wife/partner/daughter/sister…) gives birth? For example, caesarean or vaginal birth?
    a. Probe: Why do you prefer this way?
    b. Does your (wife/partner/daughter/sister…) also prefer to give birth this way? Why or why not?

56. Did your (wife/partner/daughter/sister…) decide about how she will give birth? For example, caesarean or vaginal birth?
    a. Probe: Who makes this decision?
       (note: the decision about mode of birth has not yet been made, or if he/she responds that they don't know to question 7, please instead ask: "How do you think she would plan to make this decision?")
        i. Probe: Did you influence this decision? How?
        ii. Probe: Did your family or her family influence this decision? How?
        iii. Probe: Did your friends or her friends influence this decision? How?
        iv. Probe: Did the doctor or midwife influence this decision? How?
        v. Probe: Did the media influence this decision? How?

For peer review only - http://bmjopen.bmj.com/site/about/guidelines.xhtml

Part 3. Prenatal education

57. Have you been to any antenatal care visits with your (wife/partner/daughter/sister…) ? Why or why not?
    a. *If yes:* Thinking about when you went to the antenatal care visits, what are some of the things that are most important to you?
        vi. Probe: What are some of the most important things you have learned during antenatal care? Why are they important?
        vii. Probe: How do you think antenatal care could be improved?
        viii. What do you think is missing from the antenatal care visits?
        ix. Are there any things that you would remove or change during the antenatal care visits? What are they and why would you change?

58. Thinking about antenatal care visits, what are some of the things that are most important to you to learn about?
    a. Probe: What are some of the most important things you have learned during antenatal care? Why are they important?
    b. Probe: How do you think antenatal care could be improved?

Part 4. Decision-aids

59. How did you learn about vaginal birth and caesarean section?

60. Do you feel like you have enough information to understand the options that women have for how to give birth? Why or why not?
    i. Probe: What other type of information about different modes of childbirth would you be interested to learn about?
    ii. At what point during your (wife/partner/sister…'s) pregnancy would you like to receive this information?

61. Pregnancy and childbirth are exciting times but can also be scary. Is there anything that you are afraid of or nervous about pregnancy or childbirth? Why or why not?
    a. What about during the birth, is there anything that you are afraid of? Why or why not?
    b. *If yes*:
        i. Have you spoken to anyone about these fears? Why or why not?
            1. *If yes*: What did they tell you?
            2. *If no*: Do you plan to speak to anyone about these fears? Why or why not?
        ii. What do you think could be done to help reduce this fear for you?

Part 5. Labour companionship

62. Do you plan to go to the hospital with your (wife/partner/daughter/sister…) when she gives birth? Why or why not?

63. If you do go to the hospital when your (wife/partner/ daughter/sister…) gives birth, what do you need in order to have a positive experience?

64. What type of support do you think your (wife/partner/ daughter/sister…) needs during labour and childbirth?
    a. Do you think she will receive this type of support? Why or why not?

For peer review only - http://bmjopen.bmj.com/site/about/guidelines.xhtml

Interviewer read: A labour companion is a person of the woman's choice, who can help to provide emotional support to the woman during labour and childbirth. Typically, this person would be with the woman continuously throughout labour and childbirth. This person may be the woman's husband/partner, her mother, or a friend.

65. Have you ever heard of someone providing this type of support?

66. What do you think of this type of support?

67. Do you know if labour companionship is allowed in the hospital your (wife/partner/ daughter/sister…) plan to give birth in?
    a. *If labour companionship is not allowed*: What do you think are the reasons for not allowing a labour companion?
    b. Would your (wife/partner/sister…) be allowed a labour companion if she requested it? Why or why not?

68. Have you ever provided this type of support before? (*If yes*: Could you tell me more about this?)

69. Do you think your (wife/partner/ daughter/sister…) would want to have a labour companion for her upcoming birth? Why or why not?

70. If your (wife/partner/ daughter/sister…) were to have a labour companion with her, who do you think she would prefer this person to be? Why?

71. *If your (wife/partner/ daughter/sister…) were to have a labour companion with her, what do you think she would expect from this person?*

72. *Would you be interested in being a labour companion to your (wife/partner/ daughter/sister…)? Why or why not?*

73. What would you need in order to be a good labor companion?
    a. What do you need from the woman?
    b. What do you need from the nurses and doctors?
    c. What do you need from the hospital?

74. What type of information or education do you think a labour companion would need to be able to support her?
    a. When during pregnancy do you think a woman or a nurse should start talking to a potential labour companion about their role during labour and childbirth?

75. In your opinion, what changes do you think the hospital could make in order to make it more comfortable for women to have a labour companion?

Thank you so much for your time. Is there anything else that you would like to share with me today about anything we talked about?

For peer review only - http://bmjopen.bmj.com/site/about/guidelines.xhtml

In-depth interview: partner / potential companion (postpartum)

Step 1: Introduce yourself to the participant. Describe the purpose of the interview and how information will be used. Obtain oral/written consent.
Step 2: Ask the participant to identify herself. Interviewer: fill out the information below prior to beginning the interview.
Step 2: Conduct the interview. Please remember to audio record the interview.

Interview place: ______________________________

Interview date: ______________________________

Start time: ______________________________

End time: ______________________________

Interviewer name: ______________________________

Interview identification: ______________________________

Participant information

Relationship with pregnant woman: ______________________________

Age (years): ______________________________

Marital status (Single, married/cohabitating, divorced, widowed): ____________________

Occupation (write in): ______________________________

Information on pregnant women

N° of women's interview: ______________________________

Woman's date of most recent birth:______________________________

For peer review only

For peer review only - http://bmjopen.bmj.com/site/about/guidelines.xhtml

Part 1. Values and needs surrounding the childbirth period

Note to interviewers: only ask questions 1-3 at Khon Kaen University Hospital or any hospital that allows companions.

76. How did you feel about your overall experience of your (wife/partner/daughter/sister…) giving birth?
    a. How would you describe your experience? Why?

77. Satisfaction
    a. How satisfied are you with the type of care your (wife/partner/daughter/sister…) received by your (wife/partner/daughter/sister…)during labour and childbirth?
    b. Can you give me an example of something you are very satisfied with?
    c. Can you give me an example of something you are NOT satisfied with?

78. How well do you feel that your (wife/partner/daughter/sister…) healthcare provider respected your opinions about care during labour and childbirth?
    a. Can you give me an example of a time when your (wife/partner/daughter/sister…) healthcare provider respected your opinions about care during labour and childbirth?
    b. Can you give me an example of a time when your (wife/partner/daughter/sister…) healthcare provider did NOT respect your opinions about your care during labour and childbirth?
        i. Probe: Or, an example of a time during labour and childbirth when someone else was making decision without talking with you or your (wife/partner/daughter/sister…)?

79. Place of birth
    a. Was she planning that she would give birth in this facility? Why or why not?
    b. How was the decision made about where she gave birth?
        i. Who was involved in the decision-making?

80. Thinking back to your wife/partner/sister birth, what are some of the things you value most? Why?
    a. Probe: to keep her healthy?
    b. Probe: to keep the baby healthy?
    c. Probe: for yourself personally?

Part 2. Decision-making processes regarding mode of childbirth

81. Could you tell me about the different ways that women can give birth?
    a. Probe: How did you learn about these options?

82. What about for your (wife/partner/daughter/sister…) most recent birth – how did she give birth? For example, caesarean or vaginal birth?
    a. Is this the way that you preferred your (wife/partner/daughter/sister…) would give birth? Why or why not?

    b. Probe: How did you decide that you preferred her to give birth in this way?
        i. Probe: Did your (wife/partner/daughter/sister…) influence your opinion? How?
        ii. Probe: Did your family influence your opinion? How?
        iii. Probe: Did your friends influence your opinion? How?

For peer review only - http://bmjopen.bmj.com/site/about/guidelines.xhtml

iv. Probe: Did your (wife/partner/daughter/sister…) doctor or midwife influence your opinion? How?
v. Probe: Did the media influence your opinion? How?

c. Did you discuss your opinion with your (wife/partner/daughter/sister…) doctor or midwife? If so, what was the discussion like?

83. Who decided about how she would give birth?
    a. Probe: who was involved in the decision-making?
    b. Probe: Did you influence this decision? How?
    c. Probe: Did your family or her family influence this decision? How?
    d. Probe: Did your friends or her friends influence this decision? How?
    e. Probe: Did the doctor or midwife influence this decision? How?
    f. Probe: Did the media influence this decision? How?

84. How did you learn about vaginal birth and caesarean section?

85. Did you feel like you had enough information to understand the options that your (wife/partner/daughter/sister) had for how to give birth? Why or why not?

86. Did you feel like you had sufficient time to talk to your (wife/partner/daughter/sister…) doctor or midwife about any concerns that you had about your (wife/partner/daughter/sister…) labour and childbirth? Why or why not?
    a. Can you give me an example of a time when you felt that you were able to discuss your questions or concerns with your (wife/partner/daughter/sister…) doctor or midwife?
    b. Can you give me an example of a time when you felt that you were NOT able to discuss your questions or concerns with your (wife/partner/daughter/sister…) doctor or midwife?

87. Pregnancy and childbirth are exciting times but can also be scary. Is there anything that you were afraid of or nervous about the childbirth?

88. *A decision-analysis tool could help to educate women about their options for mode of birth and how to discuss their preferences with a doctor.* Interviewer: show the woman the Vietnam decision analysis tool.
    a. Would this type of tool be helpful to you? Why or why not?
    b. What type of information would you like to have included?
    c. This type of decision tool can be paper based like this example, or could be an application for a phone. Which of these options do you prefer and why?

Part 3. Labour companionship

89. Were you at the hospital with your (wife/partner/daughter/sister…) when she gave birth? Why or why not?

90. If you have gone to the hospital when your (wife/partner/ daughter/sister…) gave birth, what did you need in order to have a positive experience?

91. What type of support do you think your (wife/partner/ daughter/sister…) needed during labour and childbirth?
    a. Do you think she will received this type of support? Why or why not?

For peer review only - http://bmjopen.bmj.com/site/about/guidelines.xhtml

Interviewer read: A labour companion is a person of the woman's choice, who can help to provide emotional support to the woman during labour and childbirth. Typically, this person would be with the woman continuously throughout labour and childbirth. This person may be the woman's husband/partner, her mother, or a friend.

92. Have you ever heard of someone providing this type of support?

93. What do you think of this type of support?

94. Do you know if labour companionship was allowed in the hospital your (wife/partner/ daughter/sister…) gave birth in?
    a. *If labour companionship was not allowed*: What do you think are the reasons for not allowing a labour companion?
    b. Had your (wife/partner/sister…) be allowed a labour companion if she had requested it? Why or why not?

95. Have you ever provided this type of support before?
    a. *If yes*: Could you tell me more about this?

96. Do you think your (wife/partner/ daughter/sister…) would have wanted to have a labour companion for her birth? Why or why not?

97. If your (wife/partner/ daughter/sister…) were to have a labour companion with her, who do you think she would prefer this person to be? Why?

98. If your (wife/partner/ daughter/sister…) were to have a labour companion with her, what do you think she would expect from this person?

99. Would you be interested in being a labour companion to your (wife/partner/ daughter/sister…)? Why or why not?

100. What would you need in order to be a good labor companion?
    a. What do you need from the woman?
    b. What do you need from the nurses and doctors?
    c. What do you need from the hospital?

101. What type of information or education do you think a labour companion would need to be able to support her?
    a. When do you think a woman should start talking to a potential labour companion about their role during labour and childbirth? (probe: at what month during the pregnancy?)

102. In your opinion, what changes do you think the hospital could make in order to make it more comfortable for women to have a labour companion?

Thank you so much for your time. Is there anything else that you would like to share with me today about anything we talked about?

For peer review only - http://bmjopen.bmj.com/site/about/guidelines.xhtml

In-depth interview: providers

Step 1: Introduce yourself to the participant. Describe the purpose of the interview and how information will be used. Obtain oral/written consent.
Step 2: Complete sociodemographic information about the participant.
Step 3: Conduct the interview. Please remember to audio record the interview.

Interview place: ________________________________

Interview date: ________________________________

Start time: ____________________________________

End time: _____________________________________

Interviewer name: _________________________________

Interview identification: ___________________________________

Participant information

Name of health facility currently employed at (write in): ______________________

Cadre and position (write in): ____

Number of years working at current health facility:__________________________________

Number of years as a (doctor/midwife/nurse) in total:_______________________________

Age (years): ____

Marital status (Single, married/cohabitating, divorced, widowed): ____________________

For peer review only

For peer review only - http://bmjopen.bmj.com/site/about/guidelines.xhtml

Decision-making processes regarding mode of childbirth

1. Could you describe what you think quality care provided during childbirth is?
    a. Could you give me an example of a situation when this kind of care was provided by you?
        i. Why do you think you were able to provide quality care in this situation?
        ii. How did your colleagues support you to provide quality care?

    b. Could you give me an example of a situation when this kind of care was NOT provided by you or by a coworker?
        i. Why do you think you weren't able to provide quality care in this situation?
        ii. Did you feel like your colleagues supported you in this situation? Why or why not?

2. In your health facility, how are decisions made about whether a woman will give birth vaginally or by caesarean section?
    i. Who is involved in making the decision, and what roles do they play?
    ii. In your health facility, what are some of the clinical indications for caesarean section?
    iii. Other than clinical indications for caesarean section, what factors might influence if a woman has a caesarean section?

3. Is assisted vaginal delivery (e.g. by vacuum or forceps) used in your facility?
    i. Probe: why or why not?
    ii. Probe: were you trained on how to provide assisted vaginal delivery? Please explain.

4. In your facility, do you think that women prefer to give birth by caesarean section or vaginally? Please explain.

5. In your health facility, how do you manage women who request to have an elective caesarean section?
    i. Why do you think women may request to have a caesarean section without a medical indication (e.g. elective caesarean)?
    ii. Who do you think influences women's decisions to have a caesarean section without a medical indication (e.g. elective caesarean)?

6. As a clinician, do you prefer for women to give birth vaginally or by caesarean section? Why?
    i. What are some of the benefits/challenges of caesarean section/vaginal birth?
    ii. Which do you think is safer: vaginal birth or caesarean section? Why?

7. In your opinion, are high rates of caesarean section a problem in your health facility? Why or why not?
    i. *Probe if yes:*
        i. Why do you think there are high rates of caesarean section in your facility?
        ii. Do you think that the caesarean section rate in your facility can be reduced? Why or why not?
        iii. Do you think that the caesarean section rate in your facility should be reduced? Why or why not?
        iv. What are the barriers to reducing high rates of caesarean section in your facility?

For peer review only - http://bmjopen.bmj.com/site/about/guidelines.xhtml

v. What could be done to reduce high rates of caesarean section in your facility?

ii. *Probe if no:*

i. Do you think that the caesarean section rate in your facility could be reduced? Why or why not?

ii. Do you think that the caesarean section rate in your facility should be reduced? Why or why not?

iii. What are the barriers to reducing high rates of caesarean section in your facility?

8. From your perspective, is a caesarean section more or less work for a healthcare provider, compared to a vaginal birth? Please explain.

9. In your opinion, do you think it is financially more profitable for providers or health facilities to conduct a caesarean section, compared to a vaginal birth?
    a. *Probe:* Why or why not?

Prenatal education and decision-analysis tool

Interviewer: The next section of this interview is about the type of health education about mode of birth that you think women would like to receive during antenatal care. I would like to ask you some questions about what you think about different topics of health education to be discussed during antenatal care.

10. In your opinion, do you think that women have sufficient knowledge about their options and the risks and benefits for different mode of birth? Why or why not?

11. In your practice, how do pregnant women they access information about their options for mode of birth?
    a. What do you think about these information resources?

12. What type of information do you think that women need to inform their preferences and decisions about their mode of birth?
    a. *Probe:* Risks of different methods, benefits of different methods, personal preferences

13. Do you think that groups of women may have different needs for information about mode of birth?
    a. *Probe if yes*: what groups of women do you think may need different information?
    b. *Probe if yes*: What type of information do you think that these women might need?

14. During antenatal care, do you (or providers conducting antenatal care in your facility) discuss with women whether they have a preference for vaginal birth or caesarean section?
    a. *IF YES, probe:* What do you discuss with the women?
    b. *IF NO, probe:* Do you think that discussing their preferences for vaginal birth or caesarean section could be helpful? Why or why not?

15. What information do you think could be included in prenatal education about vaginal birth and caesarean section?

16. At what point during a woman's pregnancy do you think they should receive this information about mode of birth? Why?

For peer review only - http://bmjopen.bmj.com/site/about/guidelines.xhtml

17. How often do you think women should receive this information about vaginal birth and caesarean section?
    a. *Probe:* Once? More than once?

18. How would do you think women should receive this information?
    a. *Probe:* Should she receive this information verbally, from her healthcare provider? Why or why not?
    b. *Probe:* Should she receive this information in a pamphlet or brochure? Why or why not?
    c. *Probe:* Should she receive this information using a computer or a mobile phone application? Why or why not?

(Interviewer: use the Vietnam decision-analysis tool as an example)
The next section of this interview is about using decision-tools (such as a computer, tablet or a smart phone) to help pregnant women with previous caesarean sections to understand their choices about mode of birth. By this, we mean whether the woman will have planned vaginal birth, trial of labour, or a caesarean section. These decision-tools would provide them with information about potential benefits and harms of the different options. They would be in addition to any regular counselling or discussions with healthcare providers. I would like to ask you some questions about what you think about these decision-tools.

19. *A decision-tool could provide descriptions of the health outcomes associated with planned vaginal birth, planned caesarean section, and emergency caesarean section. They can also ask questions about a woman's values and preferences for possible outcomes. Once this information is provided, the decision-tool can produce a recommended "preferred option", based on a woman's preferences. The woman could then bring this to her healthcare provider to discuss in more detail.* What do you think about this type of decision-tool?
    Does this description of a decision-tool sound like something that might be useful to you? Why or why not?
    a. What do you think are some of the benefits of using a decision-tool to help decide about how a woman will give birth?
        i. *Probe:* to you as a provider?
        ii. *Probe:* to the woman
    b. What do you think are some of the challenges of using a decision-tool to help decide about how a woman will give birth?
        iii. *Probe:* to you as a provider?
        iv. *Probe:* to the woman
    c. At what point during a woman's pregnancy would it be most helpful for her to have access to this type of decision-tool? Why?
    d. How might you use the results of the decision tool, or the woman's "preferred option", to discuss her options for mode of birth?
    e. Do you think that you would recommend that women use this type of decision-tool? Why or why not?

20. *These types of decision-tools can come in different formats. For example, on paper, a computer, a tablet, or a smart phone application.* What format do you think would be most helpful? Why?

Audit and feedback

Note to interviewer: use graphic of audit and feedback/Robson classification to explain to providers who don't understand. Consider using an example of maternal morbidity and mortality conference as example of audit and feedback.

For peer review only - http://bmjopen.bmj.com/site/about/guidelines.xhtml

Interviewer: The next part of the study is about ***using audit and feedback as a tool for quality improvement****. The purpose of audit and feedback is to encourage individuals and teams to follow professional standards or targets and to monitor changes and outcomes when these are used. During an audit and feedback process, an individual's or department's professional practice and/or performance is measured and compared to targets or professional standards. The results of this comparison are fed back to the individual by either a colleague, supervisor or third party, in the form of verbal or written communication. I would like to ask you some questions about what you think about audit and feedback.*

21. Could you tell me about a time where you have been involved in an audit and feedback project?
    a. *If they have been involved in an audit and feedback project:*
        i. What did you find helpful about the audit and feedback process?
        ii. What did you find challenging about the audit and feedback process?
        iii. What were the main things you learned from the audit and feedback process?
        iv. Overall, what was your opinion regarding the audit and feedback process?

22. What areas of health do you think would be most interesting and relevant for audit and feedback? For example, this might include reasons for caesarean section, severe morbidity. Why are these interesting?

Audit and feedback to improve obstetric care may include activities like critical case incident reviews, indications for caesarean section, time from decision to operation for caesarean section, decision-making processes for caesarean section, and appropriate management of complications. This may be done by reviewing individual patient records, labour and delivery logs, and observations of clinical practice.

23. How would you feel about the idea of a regular audit and feedback process in your health facility to address rising caesarean section rates?

24. What might be some of the benefits of audit and feedback may be related to caesarean section?

25. What might be some of the challenges of audit and feedback may be related to caesarean section?

26. Do you think starting an audit and feedback process may change people's behaviour in your department? Why or why not?

27. Do you think starting an audit and feedback process may change health outcomes? Why or why not?

28. What could be done in your health facility to ensure that audit and feedback is conducted in a supportive way that emphasises learning rather than punishing providers for certain behaviours?

29. How can audit and feedback be presented to you to ensure that any information gathered is "actionable" so that an individual can work to improve their practice?

30. What type of person would be the most appropriate person to:
    a. Review medical records?
    b. Analyse the data and prepare a summary report?

For peer review only - http://bmjopen.bmj.com/site/about/guidelines.xhtml

c. To present or discuss the report with you?
d. Probe: Would you prefer that this person were a colleague, supervisor, or someone external? Why?

31. Approximately how often do you think that audit and feedback processes should occur in your health facility? Please explain.

(Interviewer: do not ask these questions to antenatal nurses)
*Interviewer: The next part of the study is about the **audit and feedback tools for classifying caesarean sections**. These tools may be useful for healthcare providers and administrators to monitor which women are receiving caesarean sections, and also to help to compare caesarean section rates over time or across different health facilities and countries. This may help to design and implement interventions to make sure that an optimal caesarean section rate can occur in a specific health facility. I would like to ask you some questions about what you think of such audit and feedback tools. In order to understand drivers of rising Caesarean section rates, we need to have tools to monitor and compare caesarean section rates in a setting over time. One way to do this is the Robson classification system, which prospectively classifies women admitted for childbirth into one of ten groups.*

32. Have you heard of the Robson classification system before?
    a. *If yes:* Can you tell me what you know about the Robson classification system?
    b. *If no,* Do you know of any other classification systems to classify women giving birth?

33. *Conducting audit and feedback for caesarean section requires reviewing patient medical records and/or facility logs.* Could you tell me about how records are prepared and kept in your health facility?
    a. What is your perception regarding the completeness of labour and delivery records in your health facility?
        1. *Probe:* Do you think that labour and delivery records are complete and accurate for all or most women in your health facility? Why or why not?
    b. *Probe:* Who is responsible for recording in the medical records?
    c. *Probe:* In addition to the individual patient's record, how else is data collected and recorded on the labour and delivery ward?
    b. *Probe:* is there a facility-level logbook? If so, who is responsible for this? What type of data is recorded?

In-service training and implementation of clinical practice guidelines

34. How well do you feel your training prepared you for your current position? Please explain.

For peer review only - http://bmjopen.bmj.com/site/about/guidelines.xhtml

Interviewer: The next part of the study is about continuous training and implementation of clinical practice guidelines. This refers to the processes by which guideline recommendations are used to help healthcare providers make informed decisions about how and when to provide care in order to achieve the best health outcomes. I would like to ask you some questions about what you think about guideline implementation.

35. Are you aware of any clinical practice guidelines (algorithms/flowcharts/clinical protocols) related to obstetrics?
    a. *If yes,* which clinical practice guidelines are you familiar with?
        i. In your opinion, how valuable are the obstetrics clinical practice guidelines to your practice?
        ii. In your opinion, how accessible are these clinical practice guidelines to healthcare providers?
        iii. *Probe:* what could be done to improve the accessibility of clinical practice guidelines to other healthcare providers?
        iv. Could you describe the process of how obstetrics clinical practice guidelines are prioritised in your health facility?
        v. How are the clinical practice guidelines communicated to other healthcare providers in your facility?
        vi. How do you use clinical practice guidelines in your practice?
    b. *If no, probe:*
        i. How do healthcare providers in your facility make decisions about how to manage patients?
        ii. In your health facility, are clinical practice guidelines currently used in obstetrics?
        iii. In your opinion, what could be done to improve the accessibility of clinical practice guidelines to other healthcare providers?

36. *Imagine that your health facility will start a process of updating and implementing obstetrics clinical* practice guidelines (algorithms/flowcharts/clinical protocols). Who would need to support this initiative in order for it to be successful?
    a. *Probe:* Why would this person/these people need to support the initiative?

    b. *Probe:* How would this person/these people best support the initiative?

    c. What type of training would be helpful to ensure that all staff understand the clinical practice guidelines?
        i. What type of topics would you like to have covered during the training?
            1. Would you be interested to learn about how clinical practice guidelines were developed? Why or why not?
            2. Would you be interested to learn about the evidence behind the recommendations in clinical practice guidelines, such as the systematic reviews or clinical trials?
        ii. How long should the training last for?
        iii. How often should the training be repeated?
        iv. Where should the training be held (e.g. within the facility, outside the facility)?

    d. What resources would be needed in order to successful implement obstetrics clinical protocols?

For peer review only - http://bmjopen.bmj.com/site/about/guidelines.xhtml

e. In your opinion, what are some barriers to successful implementation of obstetrics clinical practice guidelines?

f. In your opinion, what are some facilitators to successful implementation of obstetrics clinical practice guidelines?

g. *Usually when clinical practice guidelines are implemented in health facilities, there are activities to evaluate if the guidelines are being implemented correctly and consistently*. What type of evaluation activities would be helpful to assess if obstetrics clinical practice guidelines were being implemented correctly and consistently?
   i. What format would be appropriate to feedback the evaluations to healthcare providers?
   ii. If meetings were held to feedback on the progress of obstetric clinical practice guidelines implementation, what would you like to hear discussed?
      1. Who would attend these meetings and why?
      2. How often would these meetings be held?

37. In your opinion, how important is providing pain relief for women during labour (vaginal birth only)? Why?

   a. In your facility, what pain relief options are there for women during labour (vaginal birth only)? Probe: pharmacological and non-pharmacological methods

   b. In your opinion, how important is it for women to walk around during labour (vaginal birth only)? Why?

   c. In your opinion, how important is it for women to be able to sit upright during labour (vaginal birth only)? Why?

Opinion leader education

Interviewer: The next part of the study is about using opinion leaders in a specific health facility to act as champions for change. Opinion leaders are influential individuals who are nominated by their peers to change the culture and norms of healthcare provider peer groups. For example, these individuals may be responsible for adapting clinical guidelines to a specific health facility context, and identifying measures to ensure quality improvement. I would like to ask you some questions about what you think about the use of opinion leaders in your health facility.

38. What do you think are the characteristics of a good opinion leader?

39. What do you think about the idea of using opinion leaders to adapt clinical guidelines to your health facility?

40. What type of healthcare provider would be most appropriate to act as an opinion leader for caesarean section? *(probe: nurse/midwife/doctor, what level of training)*

41. How do you think an opinion leader would be received by other healthcare providers in your health facility?

For peer review only - http://bmjopen.bmj.com/site/about/guidelines.xhtml

42. What challenges do you think an opinion leader would face if they tried to adapt and implement clinical guidelines in your health facility?

43. What type of *training* would an opinion leader need to succeed?

44. What resources would an opinion leader need to succeed?

Organization and relationships in the facility

45. Could you please describe for me what the relationship that you have with your peers is like?
    a. Could you tell me about a time when your peers supported you?
    b. Could you tell me about a time when your peers did not support you?
    c. If you are struggling to meet the demands of your work, can you look to your peers for help? How so?
    d. In your opinion, are men and women treated equally in your work place? Why or why not?

46. In your opinion, how well do doctors and midwives work together in general?
    a. How well do you think doctors and midwives communicate?
    b. What are some of the challenges in having midwives and doctors work together?
    c. Can you tell me about a time when midwives and doctors did NOT work together?
        i. Why do you think this happened?
    d. Can you tell me about a time when midwives and doctors worked very well together?
        i. Why do you think this happened?

47. Overall, how supportive do you feel that your work environment is? Please explain.

48. How do you feel about the current environment around malpractice lawsuits and legal liability for doctors?
    a. Do you feel that the health system or your health facility would support you in a legal case?
    b. How do you think that the legal environment may influence your own, or your colleagues', medical practice?

49. Do you feel afraid of malpractice lawsuits in your current work? Please explain.

50. What strategies do you employ to minimise the risk of a malpractice lawsuit?
    a. Do you think these strategies are reasonable?

For peer review only - http://bmjopen.bmj.com/site/about/guidelines.xhtml

Labour companionship

Interviewer: The next part of the study is about the type of support that women could receive during childbirth in a health facility. In some settings, a "labour companion" can provide this type of support. A labour companion is a person of the woman's choice, for example her husband, her sister, her mother, her friend, or a doula, who stays with the woman throughout the duration of labour and childbirth. I would like to ask you some questions about what you think about support from a companion during childbirth.

51. <u>What type of support do you think women need during labour and childbirth?</u>
    a. Do you think that women in your hospital receive this kind of support you have described? Why or why not?

52. <u>What do you know about labour companionship?</u>
    a. What are the benefits of labour companionship?
        i. *Probe*: What are benefits for the woman?
        ii. *Probe*: What are benefits for the providers?
        iii. *Prove*: What are benefits for the companion?
    b. Are there any harms of labour companionship?
        i. *Probe*: What are harms for the woman?
        ii. *Probe*: What are harms for the providers?
        iii. *Probe*: What are harms for the companion?

53. <u>Do you have any previous experience with working in a hospital that offered labour support?</u>
    i. If yes, what was this experience like for you as a provider?

54. <u>Do you know if labour companionship is allowed in this hospital?</u>
    a. *If labour companionship is not allowed:* What do you think are the reasons for not allowing a labour companion in this hospital?

55. <u>How could labour companionship be implemented in your hospital or other hospitals like this?</u>
    a. What would be the main challenges to implementing labour companionship?
    b. Who do you think women would prefer as a labour companion? Why?
    c. As a provider, what are your expectations from a woman's labour companion?
    d. When would a labour companion be able to be with the woman in the hospital?
    e. What would the role of the labour companion be?
        i. How could the labour companion's roles be communicated to them?
    f. At what point during the care process should women and providers start talking about labour companionship and the role of the companion?
    g. What type of information or education do you think a labour companion would need to be able to support you?
    h. How could we ensure that the companion is a person of the woman's choice, and not someone selected for her by someone else?
    i. What changes do you think the hospital could make to make it more comfortable for women to have a labour companion?
    j. If labour companionship is to be implemented in this hospital, what would ensure successful implementation?
        a. What could be done to ensure that labour companionship was sustainable in the long-term?

For peer review only - http://bmjopen.bmj.com/site/about/guidelines.xhtml

Appendix 2. Readiness assessment

1.	Name of hospital	
2.	Hospital code	

This activity is part of the readiness assessment, to explore factors to be assessed, considered and integrated into implementation plans. There are five components of this readiness assessment:

1. *Inventory of physical space and readiness;*
2. *Health workforce and model of care;*
3. *Protocols and guidelines for managing clinical care during labour and childbirth;*
4. *Continuous education and quality improvement*
5. *Assessment of data availability and access for audit and feedback; and*
6. *Understanding of labour companionship in practice.*

For each of the study health facilities, please have a member of the research team visit to conduct an observation of the labour ward and medical records. It may be most appropriate for this person to have some clinical knowledge. We expect that this activity will take approximately four to six hours to complete (depending on how busy the facility is). Prior to conducting the readiness assessment, please ensure that all members of the maternity care unit at each health facility are briefed on the purpose of the activity, what the readiness assessment will entail, and how they may be of assistance. This will help to ensure that the readiness assessment (and other research activities) will be welcomed by the unit.

Initial inventory

3.	# of Deliveries (1 October 2019 – 31 January 2020)	
4.	No of delivery handled exclusively by a certain obstetrician (private)	
5.	Women not handled exclusively by a certain obstetrician (non-private)	
6.	No. of Caesarean section (1 October 2019 – 31 January 2020)	
7.	Elective (pre-labour c-section)<br>*Note: if this information is not readily available, then please ignore for now.*	
8.	Emergency (intrapartum c-section)	
9.	No. of vaginal instrumental delivery (1 October 2019 – 31 January 2020)	

For peer review only - http://bmjopen.bmj.com/site/about/guidelines.xhtml

Part 1. Inventory of physical space and resources

Please observe the physical space of the labour, delivery and postnatal wards. If these are in separate areas (e.g.: women in latent labour in a labour ward, women in active labour in a separate room/delivery ward, separate postnatal ward), please assess both areas according to all points below. Please provide a narrative description of the wards, as well as a visual depiction.

Description of the physical space

Please detail:

# of beds in labour /admission room (1st stage of labour room):	
# of beds in labour / childbirth room (2nd stage of labour room):	
# Ultrasound machine in the labour room (indicate number working)	
# Electronic fetal heat rate monitor (CTG) (indicate number of devices working)	

Description of any curtains, dividers or other means of protecting a woman's privacy

Description of the potential for crowding. For example, how many beds are present? Are they currently or usually full? What happens if there is overcrowding?

For peer review only

For peer review only - http://bmjopen.bmj.com/site/about/guidelines.xhtml

Description of the visiting hours and allowable visitors (check if visually displayed and ask an administrator) (please take a photo if there is a sign)

Description of the accessibility to toilets or washrooms

Description of overnight accommodation for family members/friends of women (check if visually displayed and ask an administrator)

Description of the **operating theatres** (how is access to the theatre, is there one theatre reserved for obstetrics, is the theatre on the same floor? How are handwashing facilities)

Please detail how many theatres are exclusively to perform a CS (#)	

For peer review only - http://bmjopen.bmj.com/site/about/guidelines.xhtml

Please sketch the physical space

For peer review only

For peer review only - http://bmjopen.bmj.com/site/about/guidelines.xhtml

Part 2. Health workforce and model of care.

Completing this section may require both observation of the labour ward and a discussion with staff, e.g.: a matron-in-charge or head of obstetrics.

Please indicate below the **staffing available for the delivery ward alone. Please include all staff on the payroll**. Please do not count the staff only working on ANC or PNC. If staff works part-time in the maternity, try to summarise to have full-time-equivalents.

STAFFING – LABOUR AND DELIVERY WARD ONLY		
	**Staff category**	Number employed (Full-time equivalent)
1.	Obstetrician	
2.	Anaesthesiologists	
3.	Paediatrician	
4.	Medical doctors (graduated, no specialization)	
5.	Residents (medics in training)	
6.	Nurses / nurse-midwives	
7.	Distinct midwives (exclusively trained on midwifery)	
8.	Delivery assistants / auxiliary nursing staff	
9.	Cleaners / other auxiliary non-nursing staff	

STAFFING ON SHIFTS IN LABOUR AND DELIVERY			
	**Staff category**	**DAY SHIFT** Number employed (Full-time equivalent)	**NIGHT SHIFT** Number employed (Full-time equivalent)
1.	Obstetrician		
2.	Anesthesiologists		
3.	Pediatrician		
4.	Medical doctors (graduated, no specialization)		
5.	Residents (medics in training)		
6.	Nurses / nurse-midwives		
7.	Distinct midwives (exclusively trained in midwifery)		
8.	Delivery assistants / auxiliary nursing staff		
9.	Cleaners / other auxiliary non-nursing staff		

For peer review only - http://bmjopen.bmj.com/site/about/guidelines.xhtml
For peer review only

Please indicate below the **staffing available for the <u>ANTENATAL CARE ward</u> alone. Please include all staff on the payroll**. Please do not count the staff only working on ANC or PNC. If staff works part-time in the maternity, try to summarise to have Full-time-equivalents.

STAFFING – ANTENATAL CARE WARD ONLY		
	**Staff category**	Number employed (Full-time equivalent)
1.	Obstetrician	
2.	Anesthesiologists	
3.	Pediatrician	
4.	Medical doctors (graduated, no specialization)	
5.	Residents (medics in training)	
6.	Nurses / nurse-midwives	
7.	Distinct midwives (exclusively trained on midwifery)	
8.	Delivery assistants / auxiliary nursing staff	
9.	Cleaners / other auxiliary non-nursing staff	

STAFFING ON SHIFTS IN ANTENATAL CARE WARD			
	**Staff category**	**DAY SHIFT** Number employed (Full-time equivalent)	**NIGHT SHIFT** Number employed (Full-time equivalent)
1.	Obstetrician		
2.	Anesthesiologists		
3.	Pediatrician		
4.	Medical doctors (graduated, no specialization)		
5.	Residents (medics in training)		
6.	Nurses / nurse-midwives		
7.	Distinct midwives (exclusively trained in midwifery)		
8.	Delivery assistants / auxiliary nursing staff		
9.	Cleaners / other auxiliary non-nursing staff		

For peer review only - http://bmjopen.bmj.com/site/about/guidelines.xhtml

Please explain the on-call system: Is the doctor to perform a CS in the hospital even at night, or is s/he on-call at home? Are there other resource-persons one can call in if needed?

Part 3. Protocols and guidelines for managing clinical care during labour and childbirth.

Please list the protocols available and in use in this hospital. Please indicate if the head of maternity indicates the presence. If so ask about the type of guidelines (national standard guidelines e.g by MoH or professional organisation) and if the guideline is physically available, e.g in a folder or displayed at the wall.

**S. No**	**List of Protocols**	Present<br>1. Yes<br>2. No	Type<br>1. National Standards<br>2. Hospital Specified	Displayed<br>Yes<br>No
1.	Partograph use / fetal monitoring			
2.	Active Management of Third Stage of labor			
3.	Postpartum haemorrhage management			
4.	Blood transfusion			
5.	Pre-term labor			
6.	Induction / augmentation of labour			
7.	Antenatal steroids			
8.	Obstructed labour			
9.	Previous CS (trial of labour)			

For peer review only - http://bmjopen.bmj.com/site/about/guidelines.xhtml

Completing this section may require both observation of the labour ward (e.g.: posters or signs) and a discussion with staff, e.g.: a matron-in-charge or head of obstetrics. If possible, make a photo if there are guidelines on the wall.

Could you describe how any clinical protocols or guidelines for managing *routine or complicated labour and childbirth* care were developed or adapted, and updated? Is there a team in the hospital taking care of this? Can you explain how are these clinical protocols/guidelines used?

Continuous education and quality improvement

Specify for trainings provided in the last 1 year

**List of training provided**	Type<br>1. National Standard training<br>2. Hospital Specified	Was the training provided to<br>1. All providers<br>2. Only doctors<br>3. Only nurse-midwives
Partograph use / fetal monitoring		
Active Management of Third Stage of labour		
Postpartum haemorrhage management		
Blood transfusion		
Pre-term labor /		
Introduction / augmentation of labour		
Antenatal steroids		
Obstructed labour		
Previous CS (trial of labour)		

For peer review only - http://bmjopen.bmj.com/site/about/guidelines.xhtml

For peer review only

Could you please describe other quality improvement activities ongoing in this facility. How are nurses and doctors informed about new knowledge and guidelines? Do you need to go regularly to refresh knowledge to workshops or trainings?

Do you do on-the-job training / mentoring of younger colleagues? Is there a system of supervision? Is there a system to discuss difficult cases, e.g. during a morning report? Do you do audits of cases?

Please explain how informed consent is obtained for caesarean section (oral, written, included in medical record, etc).

For peer review only - http://bmjopen.bmj.com/site/about/guidelines.xhtml
For peer review only

Part 4. Assessment of facility medical records and data management systems

Please review individual medical records to assess the information currently collected related to key obstetric variables at an individual level. Please also review any facility-level register, log book or other records to assess information currently collected related to key obstetric variables at a facility level. It may be helpful to discuss the medical and facility records with the staff, e.g.: a matron-in-charge or head of obstetrics. Collecting this information will help to inform the implementation of the Robson classification system, e.g.: to identify what data is already routinely collected, and what data may need to be added to routine data collection.

Please explain which information is used. Description of other information routinely collected about caesarean section (e.g. provider, morbidity)

For peer review only

There are typically two different places for documentation a) facility-level register, log book, or other records collating key obstetric variables and b) case notes/patient records. Now first we like to have information on the first type:

Please describe the description of any facility-level register, log book, or other records collating key obstetric variables at the facility-level. Please include whether this register is paper-based or electronic, when it is updated, when and how information is summarised and how often it is reported. Please take a photo (covering patient names).

Who is responsible for completing the facility-level register?

For peer review only - http://bmjopen.bmj.com/site/about/guidelines.xhtml

How often is the facility-level register updated and summaries are prepared?

How is the information about facility-level key obstetric variables and outcomes currently integrated into audit and feedback?

Description of the consistency of reporting for these indicators (e.g.: consistently reported across all records reviewed, some data missing – be specific).

Who is present during audit and feedback sessions, and who leads the sessions?

For peer review only - http://bmjopen.bmj.com/site/about/guidelines.xhtml

Description of the health facility's "decision-to-incision" time to perform a caesarean section. If not available, please specify.

Now concerning the second type of records: case notes/medical records: Please describe the medical record structure (e.g. electronic or paper), and who keeps the records (e.g. woman or provider)? Is there a standard form? Is the WHO partograph used? Are the data used for audits and feedback?

Is there any regular feedback of these reports to the providers? If so, how often and in what format?

Review of medical records to assess if key obstetric variables needed for Robson classification are correctly and consistently reported ***at an individual level.*** *For each variable, please (1) ask the administrator how it is reported, and (2) observe a subset of records to assess how variable is actually reported (e.g. 5-10 medical records).*

Parity	
**Administrator response**	**Observation of records**

For peer review only - http://bmjopen.bmj.com/site/about/guidelines.xhtml

Previous caesarean section	
Administrator response	Observation of records

Onset of labour (spontaneous, induced, no labour/pre-labour caesarean section)	
Administrator response	Observation of records

Gestational age (preterm <37 weeks, term $\geq$ 37 weeks)	
Administrator response	Observation of records

Fetal presentation or lie (cephalic, breech, transverse)	
Administrator response	Observation of records

Number of fetuses (singleton, multiple)	
Administrator response	Observation of records

Who is responsible for completing the individual-level medical records? Does anyone else check for consistent and correct reporting?

For peer review only - http://bmjopen.bmj.com/site/about/guidelines.xhtml
For peer review only

Based on the observations and record assessment, what would you consider to be the most appropriate method of implementation of the Robson classification system (manually, using a spreadsheet or automatic calculator, or via electronic records)? Please explain.

[*Ask the administrator*] What type of person would be the most appropriate facility-level champion to implement the Robson classification system, and why? [e.g.: type of provider, what skills this person would have]

[*Ask the administrator*] Who is the best person to *record* data for Robson classification, and why?

[*Ask the administrator*] Who is the best person to *analyse* data for Robson classification, and why?

For peer review only

For peer review only - http://bmjopen.bmj.com/site/about/guidelines.xhtml

[*Ask the administrator*] Who is the best person to *report and present* data for Robson classification, and why?

Part 5. Understanding of labour companionship in practice

Completing this section may require both observation of the labour ward and a discussion with staff, e.g.: a matron-in-charge or head of obstetrics. If companionship is not currently allowed at the facility, please specify below.

Description of who is currently allowed to act as a companion for the woman

Description of for what periods of time companionship is offered (e.g.: from admission to discharge, during labour but not childbirth, only at childbirth)

Please detail at what stages / and time of the day companions are allowed:

Labour / first stage	Yes / no
Delivery / second stage	Yes / no
Immediate postpartum period (first hour or two)	Yes / no
Postnatal ward	Yes / no
Day-time	Yes / no
Night-time	Yes / no

For peer review only - http://bmjopen.bmj.com/site/about/guidelines.xhtml

For peer review only

Description of the roles that companions usually undertake (e.g.: emotional support, providing food/water/tea to the woman, supporting staff)

Description of how staff currently interact with companions

Existence and content of any orientation materials, protocols, or guidelines related to how staff should work with companions, or on the role of companions. If no materials exist, please state this.

Any other feedback, observations or reflections

For peer review only - http://bmjopen.bmj.com/site/about/guidelines.xhtml

BMJ Open

Factors influencing the implementation of labour companionship: formative qualitative research in Thailand

Journal:	*BMJ Open*
Manuscript ID	bmjopen-2021-054946.R1
Article Type:	Original research
Date Submitted by the Author:	10-Feb-2022

Complete List of Authors:	Rungreangkulkij, Somporn; Khon Kaen University Faculty of Nursing<br>Ratinthorn, Ameporn; Mahidol University Faculty of Nursing<br>Lumbiganon, Pisake; Khon Kaen University Faculty of Medicine, Obstetrics and Gynaecology<br>Zahroh, Rana; The University of Melbourne, Gender and Women's Health Unit, Centre of Health Equity, Melbourne School of Population and Global Health<br>Hanson, Claudia; Karolinska Institute, Public Health Sciences; London School of Hygiene and Tropical Medicine Faculty of Infectious and Tropical Diseases, Dept of Disease Control<br>Dumont, Alexandre; University of Paris, CEPED, Institute for Research on Sustainable Development, IRD-Université de Paris, ERL INSERM SAGESUD<br>de Loenzien, Myriam; Centre Population et Developpement,<br>Betran, Ana Pilar; World Health Organization, 8. UNDP/UNFPA/UNICEF/World Bank Special Program of Research, Development and Research Training in Human Reproduction (HRP), Department of Sexual and Reproductive Health and Research<br>Bohren, Meghan ; University of Melbourne School of Population and Global Health, Gender and Women's Health Unit, Centre for Health Equity

**Primary Subject Heading**:	Global health
Secondary Subject Heading:	Health policy, Health services research, Public health, Qualitative research
Keywords:	OBSTETRICS, PUBLIC HEALTH, International health services < HEALTH SERVICES ADMINISTRATION & MANAGEMENT, Quality in health care < HEALTH SERVICES ADMINISTRATION & MANAGEMENT, QUALITATIVE RESEARCH

SCHOLARONE™
Manuscripts

I, the Submitting Author has the right to grant and does grant on behalf of all authors of the Work (as defined in the below author licence), an exclusive licence and/or a non-exclusive licence for contributions from authors who are: i) UK Crown employees; ii) where BMJ has agreed a CC-BY licence shall apply, and/or iii) in accordance with the terms applicable for US Federal Government officers or employees acting as part of their official duties; on a worldwide, perpetual, irrevocable, royalty-free basis to BMJ Publishing Group Ltd ("BMJ") its licensees and where the relevant Journal is co-owned by BMJ to the co-owners of the Journal, to publish the Work in this journal and any other BMJ products and to exploit all rights, as set out in our licence.

The Submitting Author accepts and understands that any supply made under these terms is made by BMJ to the Submitting Author unless you are acting as an employee on behalf of your employer or a postgraduate student of an affiliated institution which is paying any applicable article publishing charge ("APC") for Open Access articles. Where the Submitting Author wishes to make the Work available on an Open Access basis (and intends to pay the relevant APC), the terms of reuse of such Open Access shall be governed by a Creative Commons licence – details of these licences and which Creative Commons licence will apply to this Work are set out in our licence referred to above.

Other than as permitted in any relevant BMJ Author's Self Archiving Policies, I confirm this Work has not been accepted for publication elsewhere, is not being considered for publication elsewhere and does not duplicate material already published. I confirm all authors consent to publication of this Work and authorise the granting of this licence.

For peer review only

Factors influencing the implementation of labour companionship: formative qualitative research in Thailand

Authors:

Somporn Rungreangkulkij[1]
Ameporn Ratinthorn[2]
Pisake Lumbiganon[3]
Rana Islamiah Zahroh[4]
Claudia Hanson[5, 6]
Alexandre Dumont[7]
Myriam de Loenzien[7]
Ana Pilar Betrán[8]
Meghan A. Bohren[4*]

* Corresponding author: Meghan A. Bohren meghan.bohren@unimelb.edu.au

Affiliations:

1. Faculty of Nursing, Khon Kaen University, Khon Kaen, Thailand
2. Faculty of Nursing, Mahidol University, Bangkok, Thailand
3. Department of Obstetrics and Gynaecology, Faculty of Medicine, Khon Kaen University, Khon Kaen, Thailand
4. Gender and Women's Health Unit, Centre for Health Equity, School of Population and Global Health, University of Melbourne, Melbourne, Australia
5. Department of Global Public Health, Karolinska Institutet, Stockholm, Sweden
6. London School of Hygiene and Tropical Medicine, London, United Kingdom
7. CEPED, Institute for Research on Sustainable Development, IRD-Université de Paris, ERL INSERM SAGESUD, Paris, France
8. UNDP/UNFPA/UNICEF/World Bank Special Program of Research, Development and Research Training in Human Reproduction (HRP), Department of Sexual and Reproductive Health and Research, World Health Organization, Geneva, Switzerland

For peer review only

For peer review only - http://bmjopen.bmj.com/site/about/guidelines.xhtml

Abstract

Introduction

WHO recommends that all women have the option to have a companion of their choice throughout labour and childbirth. Despite clear benefits of labour companionship, including better birth experiences and reduced caesarean section, labour companionship is not universally implemented. In Thailand, there are no policies for public hospitals to support companionship. This study aims to understand factors affecting implementation of labour companionship in Thailand.

Methods

This is formative qualitative research to inform the “Appropriate use of caesarean section through QUALIty DECision-making by women and providers” (QUALI-DEC) study, to design, adapt and implement a strategy to optimize use of caesarean section. We use in-depth interviews and readiness assessments to explore perceptions of healthcare providers, women, and potential companions about labour companionship in eight Thai public hospitals. Qualitative data were analysed using thematic analysis, and narrative summaries of the readiness assessment were generated. Factors potentially affecting implementation were mapped to the Capability, Opportunity, and Motivation behaviour change model (COM-B).

Results

127 qualitative interviews and eight readiness assessments are included in this analysis. The qualitative findings were grouped in four themes: benefits of labour companions, roles of labour companions, training for labour companions, and factors affecting implementation. The findings showed that healthcare providers, women, and their relatives, all had positive attitudes toward having labour companions. The readiness assessment highlighted implementation challenges related to training the companion, physical space constraints, overcrowding, and facility policies, reiterated by the qualitative reports.

Discussion

If labour companions are well-trained on how to best support women, help them to manage pain, and engage with healthcare teams, it may be a feasible intervention to implement in Thailand. However, key barriers to introducing labour companionship must be addressed to maximise the likelihood of success mainly related to training and space. These findings will be integrated into the QUALI-DEC implementation strategies.

For peer review only - http://bmjopen.bmj.com/site/about/guidelines.xhtml

Thai abstract

บทนำ

องค์การอนามัยโลกมีข้อเสนอแนะว่าผู้หญิงทุกคนควรได้รับโอกาสให้มีทางเลือกที่จะมีเพื่อนช่วยคลอดตลอดระยะเวลาเจ็บครรภ์คลอดจนถึงเด็กคลอดออกมา ทั้ง ๆ ที่มีข้อมูลเกี่ยวกับประโยชน์ของการมีเพื่อนช่วยคลอดอย่างชัดเจน เช่น ประสบการณ์การคลอดที่ดี ลดอัตราการผ่าตัดคลอด แต่พบว่าการมีเพื่อนช่วยคลอดกลับไม่ได้ถูกนำมาใช้อย่างแพร่หลาย สำหรับประเทศไทยการมีเพื่อนช่วยคลอดยังไม่ถูกนำมาใช้กำหนดเป็นนโยบายสำหรับโรงพยาบาลในสังกัดกระทรวงสาธารณสุข การวิจัยนี้มีวัตถุประสงค์ เพื่ออธิบายปัจจัยที่ส่งผลต่อการใช้เพื่อนช่วยคลอดในประเทศไทย

วิธีการ

การวิจัยเชิงคุณภาพระยะก่อรูปนี้เพื่อใช้ในการให้ข้อมูล สำหรับ “โครงการวิจัยการตัดสินใจของผู้หญิงและผู้ให้บริการสุขภาพอย่างมีคุณภาพต่อการใช้วิธีการผ่าตัดคลอด” (QUALI-DEC) study เพื่อนำมาใช้ออกแบบ การปรับวิธีการ และการลงปฏิบัติ เพื่อให้การผ่าตัดคลอดมีประโยชน์สูงสุด การศึกษานี้ใช้การเก็บข้อมูลโดยการสัมภาษณ์เชิงลึก การประเมินความพร้อม เพื่อทำความเข้าใจ การรับรู้ของผู้ให้บริการ ผู้หญิง และผู้ที่มีโอกาสเป็นเพื่อนช่วยคลอดเกี่ยวกับการมีเพื่อนช่วยคลอดจากโรงพยาบาลของรัฐแปดโรงพยาบาล วิเคราะห์ข้อมูลโดยการวิเคราะห์ประเด็น และการพรรณนา วิเคราะห์ปัจจัยที่ส่งผลต่อการนำใช้เพื่อนช่วยคลอดตามกรอบ ความสามารถ โอกาส แรงจูงใจและ และการเปลี่ยนแปลงพฤติกรรม (COM-B)

ข้อค้นพบ

ข้อมูลที่ใช้ในการวิเคราะห์ครั้งนี้ได้มาจากการสัมภาษณ์จำนวน 127 คน และจากแบบประเมินความพร้อมจากแปดโรงพยาบาล ข้อค้นพบจากงานวิจัยเชิงคุณภาพแบ่งออกได้เป็นสี่ประเด็น ดังนี้: ประโยชน์การมีเพื่อนช่วยคลอด บทบาทเพื่อนช่วยคลอด การฝึกอบรมเพื่อนช่วยคลอด และปัจจัยที่ส่งผลต่อการปฏิบัติ ข้อค้นพบแสดงให้เห็นว่า ผู้ให้บริการทางการแพทย์ ผู้หญิงและญาติของพวกเธอ มีทัศนคติทางบวกต่อการมีเพื่อนช่วยคลอด ข้อมูลจากการประเมินความพร้อมและข้อมูลเชิงคุณภาพมีความสอดคล้องกันที่แสดงให้เห็นความท้าทายในการให้มีเพื่อนช่วยคลอดได้ คือ การฝึกอบรมเพื่อนช่วยคลอด ข้อจำกัดพื้นที่ทางกายภาพ การแออัด และนโยบายของโรงพยาบาล

การอภิปรายผล

For peer review only

หากเพื่อนช่วยคลอดได้รับการฝึกอบรมอย่างดีเกี่ยวกับ วิธีการสนับสนุนให้กำลังใจผู้หญิง การช่วยลดความปวด และการติดต่อกับผู้ให้บริการ เป็นกิจกรรมที่สามารถนำใช้เพื่อนช่วยคลอดสำหรับโรงพยาบาลในประเทศไทยได้ แต่อุปสรรคในการนำใช้เพื่อนช่วยคลอดจะต้องคำนึงถึงโอกาสที่จะเพิ่มการประสบความสำเร็จที่มีความสัมพันธ์กับการอบรมและพื้นที่ทางกายภาพ ข้อค้นพบนี้ได้ถูกนำเสนอในยุทธศาสตร์ของโครงการ the QUALI-DEC

For peer review only

For peer review only - http://bmjopen.bmj.com/site/about/guidelines.xhtml

Strengths and limitations of this study

- Labour companionship has important benefits for the woman and baby, and is recommended by WHO. This is the first study to understand needs and preferences related to labour companionship, and map factors that might affect implementation of labour companionship in Thailand.
- We found that implementation of labour companionship is feasible if labour companions and health workers are well-trained on how to best support women and engage with one another. Addressing key barriers to introducing labour companionship can include changes to the physical environment, implementing facility-level policies on labour companionship, and context-specific solutions to minimise fears on lawsuits and infection.
- A key strength of our study is the triangulation of qualitative research and facility readiness assessments, and mapping of key factors affecting implementation of labour companionship to the COM-B model of behaviour change.
- Using the COM-B model to guide analysis, we show how to use our formative research findings to guide intervention design and support a systematic, targeted, and theory-based development of implementation strategies for labour companionship.
- While our research was conducted in eight public hospitals across different regions of Thailand, the findings may not be transferrable to all settings in Thailand, as most study hospitals were in urban settings with high caesarean section rates.

For peer review only - http://bmjopen.bmj.com/site/about/guidelines.xhtml

Introduction

Efforts to improve maternal health globally have shifted in recent years to improving quality of care. A critical component of quality of care is the person's 'experience of care', which the World Health Organization (WHO) has defined as ensuring that all pregnant people are treated with respect and dignity, have effective communication with health workers, and access to emotional support that meets their needs (1). Within labour and childbirth care, supporting women to have a labour companion of their choice present is an effective way to improve women's experiences by providing respectful care and emotional support (2, 3). Labour companionship refers to a person of the woman's choice, who accompanies the woman continuously throughout labour and childbirth; typically this is the woman's partner or husband, friend or family member (4). Labour companionship empowers women in several key ways: improving communication between women and health workers, helping women with non-pharmacological pain relief, acting as advocates to help voice the woman's preferences, providing practical support such as massage and hand-holding, and providing emotional support as a continuous presence (4).

Labour companionship has important benefits for both the woman and baby. A Cochrane intervention review analysed the impact of continuous support for women during labour and childbirth from 26 studies conducted with over 15,000 women in 17 countries, and found that women with continuous support were more likely to have a spontaneous vaginal birth, and less likely to report negative ratings of or feelings about their childbirth experience, or to have a caesarean birth (5). Women with labour companionship also have a shorter duration of labour and better five-minute Apgar scores for their babies. Based on this evidence, WHO recommends that all women have the opportunity to have a labour companion of their choice with them throughout labour and childbirth (3).

Despite clear evidence of benefit, implementation of labour companionship in health facilities across the world remains sub-optimal. A Cochrane qualitative evidence synthesis highlighted several factors affecting implementation, including women and health workers not recognizing the benefits of labour companionship, labour companionship viewed as a 'nice to have' but not essential service, physical space constraints on labour wards and thus difficulties to maintain privacy, and integrating labour companions into part of the care team (4).

For peer review only

Context of labour companionship in Thailand

In Thailand, labour companions are not typically allowed in most public and some private hospitals. Most public hospitals have a policy allowing women's relative to wait outside the labour room, with certain hours allocated to allow relatives or friends to visit the women in the labour room, typically during lunch or dinner time. Anecdotally, some reasons for not allowing labour companionship were the concern about infection risks (even prior to COVID-19) and maintaining the privacy of women, who normally share rooms, especially from other male companions. With increasing access to mobile phones, there are also emerging concerns about pictures and audio video recordings, which may be used in potential litigation cases against medical teams. Similar to the results of the Cochrane review, a quasi-experimental study in eastern Thailand compared the effect of companionship on primiparous women's experiences and found that women with companionship were more satisfied with their childbirth experiences, but no significant differences in self-reported suffering or ability to cope with labour pain (6).

The QUALI-DEC Project

In the context of sustained growing caesarean section rates in Thailand, the Ministry of Health and other stakeholders are examining factors underlying the increase and interventions to optimize its use. The QUALI-DEC study: "Appropriate use of caesarean section through **QUALi**ty **DEC**ision-making by women and providers" (7) aims to design, adapt and evaluate a multi-faceted strategy, for the appropriate use of caesarean section in Argentina, Burkina Faso, Thailand and Viet Nam. The QUALI-DEC strategy is designed to combine four key components: 1) Opinion leaders to implement evidence-based clinical guidelines; 2) Caesarean audits and feedback to help providers identify potentially avoidable caesarean sections; 3) A Decision Analysis Tool (DAT) to help women make an informed decision on mode of birth; and 4) Implementation of WHO recommendations on companionship during labour and childbirth (7). Labour companionship is included as a QUALI-DEC intervention component given the association between continuous support and increased chance of vaginal birth (5), as well as due to emerging evidence that companionship may improve women's experience of care and reduce mistreatment during childbirth (8, 9).

The QUALI-DEC strategy supports the woman to choose any person to act as her labour companion. The QUALI-DEC research team and implementation partners will co-develop and tailor a model for labour companionship in each hospital that includes information on 1) changing hospital policy to allow for labour companionship, 2) establishing eligibility criteria for women and companions, 2) identifying how health workers can help women to choose and train the labour companion, 3) defining how health workers engage with women and companions, how many companions are

For peer review only

For peer review only - http://bmjopen.bmj.com/site/about/guidelines.xhtml

allowed, and when they are present, 5) designing modifications for the physical space to accommodate companions, and 6) developing educational tools for companions on how to support women. Based on the formative research conducted among the local stakeholders in Thailand, the aim of this paper is to describe the needs and preferences of women, potential companions, and healthcare providers related to labour companionship, and to map factors that might affect implementation of labour companionship in Thailand, using a behaviour change model.

Methods

This is a formative qualitative study using a health facility readiness assessment and in-depth interviews (IDIs) with women, potential companions, and healthcare providers, described in detail in the study protocol (10) and below. In short, the readiness assessment and IDIs explored the needs and preferences of these key stakeholders to introduce labour companionship in each setting. During the analysis, we conceptualised findings from the readiness assessment and IDIs as 'factors potentially affecting implementation of labour companionship', and used behaviour change frameworks to map the findings in order to better understand what is needed to develop effective intervention implementation strategies. This paper is reported according to the consolidated criteria for reporting qualitative research (COREQ) guidance (11).

Eight hospitals in Thailand were purposively selected for the QUALI-DEC project according to the willingness to participate, programmatic activities, country priorities, and geographical representation (Table 1). The formative research was conducted in these eight hospitals, where caesarean section rate ranged from 34.3-56.9%.

Participants and recruitment

Five groups of participants were identified for this study: 1) pregnant women, 2) postpartum women, 3) a person identified by the woman as someone she would have liked as a companion (potential companions; before birth), 4) potential companions (after birth), and 5) healthcare providers (doctors, nurse-midwives) and administrators or managers. Pregnant women and postpartum women aged 18 to 49 years who attended antenatal and/or postnatal care at the study hospitals were invited to participate in in-depth interviews (IDIs), aiming for diversity (mix of urban or rural residence, parity, age, and ethnicity - target per facility: 2-3 pregnant and 2-3 postpartum women). Initially, nurse-midwives explored the interest of women during antenatal care or postnatal care visits, and if they were potentially interested in participating, then the research team approached women face-to-face. The pregnant and postpartum women who participated in the study identified a person who they

For peer review only - http://bmjopen.bmj.com/site/about/guidelines.xhtml

would have liked to be their labour companion ("potential companion"), and the research team approached the potential companions face-to-face to participate in an IDI (target per facility: 2-3 potential companions before birth and 2-3 after birth). Typically, the potential companion was already on the hospital grounds. Healthcare providers working on the antenatal, delivery and postnatal wards of the study hospitals and healthcare administrators were contacted by the research team and invited to participate in IDIs, with considerations for a diverse group based on age, gender and years of working experience (target per facility: 2-3 nurse-midwives, 2-3 doctors, 2 administrators). We prespecified the target sample size for each type of participant to account for the variable contexts and patient populations in each facility. No participants approached refused to participate.

Data collection

After agreeing to participate and completing a consent form, the research team conducted IDIs in Thai at the respective health facility. IDIs lasted 30-90 minutes, had no other people present, were audio-recorded, and participants received 500 Baht (USD$16) compensation for their time. General conversation was initiated prior going to main interview questions to build rapport. Data were collected from July to October 2020. All audio recordings were transcribed verbatim in Thai, complemented with field notes. De-identified transcripts were stored on a password protected computer. There was no further contact with the research participants after the IDI.

The interview guides were developed based on the implementation challenges identified in the Cochrane qualitative review (4) and covered a range of topics including: 1) values and needs around the childbirth period, 2) prenatal education, 3) preferences and decision-making processes regarding mode of birth, and 4) labour companionship (Appendix 1: interview guide). Interview guides were piloted and refined prior to data collection. This analysis focuses on the labour companionship module.

In addition to IDIs, a readiness assessment was conducted to describe and assess the service delivery context ahead of the intervention implementation, and was carried out concurrently with the IDIs (Appendix 2: readiness assessment). The readiness assessment provides a systematic approach to assessing readiness to engage in the implementation, in order to inform and tailor the interventions in a way best suitable to the local context (10). Readiness assessments were conducted by members of the QUALI-DEC research team who were professors of nursing, but not employed by the study hospitals. During data collection, the researchers used a semi-structured form to observe the service delivery context in each facility setting related to possibility or barriers for companionship

For peer review only - http://bmjopen.bmj.com/site/about/guidelines.xhtml

implementation such as the sign for visiting information, physical environment in latent room, labour room, and post-partum room (10).

Reflexivity

The QUALI-DEC research team consists of Thai and international social scientists, nurses, doctors, and epidemiologists with maternal health expertise. The research team believed that labour companionship is beneficial for women and families, and may help reduce caesarean section rates. The research team was aware of their assumptions and mindful through the study process to mitigate any potentially negative biases that could influence participant responses or interpretations of responses. Six members of research team conducted the IDIs, all were female nursing professors with extensive qualitative experience, no prior relationship with any participants, and did not work at the study sites. Prior to starting data collection, the research team underwent a three-day training on caesarean section globally and in Thailand, QUALI-DEC project, and data collection and management.

Data analysis

Thematic analysis was performed by hand according to the following steps: organizing the data; generating categories, themes, patterns; testing emergent hypothesis; searching for alternative explanations (12). Four members of the research team were involved in the data analysis. First, the researchers repeatedly read the interview transcripts to develop initial codes of the data. Secondly, the researchers conducted a systematic identification of themes from the codes such as support, being a representative, and shorten labour. Thirdly, from the themes and codes, researchers identify emerging patterns from the data, such as benefits of having labour companion. Lastly, the researchers review the coded data extracts for each theme to consider whether they appear to form a coherent pattern. In this stage, the research team considered how the different themes were similar and different across different participant groups (e.g. women and healthcare providers), and explored hypothesis for why these similarities and differences may exist. If we found inadequacies in the initial coding and themes, we revisit the themes again and iterated on necessary changes when needed. For trustworthiness, during data analysis the findings were discussed among the research team and emergent findings were presented to a representative obstetrician (QUALI-DEC opinion leader) from the study settings. Key themes emerging from the IDIs were combined with data from the readiness assessment to identify and prioritize barriers, and to develop potential implications for implementation. Data analysis was conducted in Thai in order to retain the original meaning, and excerpts from the interview transcripts in this article were translated by a bilingual Thai-English translator who is a member of the research team.

For peer review only - http://bmjopen.bmj.com/site/about/guidelines.xhtml

The research findings were then conceptualised as factors potentially affecting implementation, and mapped to the Capability, Opportunity, and Motivation model of behaviour change (COM-B) (13). The COM-B model theorises that for a desired behaviour to occur (e.g. labour companionship), individuals must have the **c**apability, **o**pportunity, and **m**otivation to enact the behaviour. Capability refers to factors such as attention, decision-making, knowledge, and skills (13). Opportunity refers to how environments influence behaviour, and includes both physical (e.g. access to supplies and resources, staffing, infrastructure) and social (e.g. team-work, support, practice norms, social and professional identities) contexts (13). Motivation refers to the internal processes that direct and encourage behaviours to occur or not, and includes factors such as perceived benefits, risks and consequences, emotions, and priorities (13). The COM-B model has been widely used in implementation research to improve implementation and to explore barriers and facilitators to changing clinical practice. By identifying factors (e.g. barriers and facilitators) that may affect implementation, teams can then design implementation strategies to address these factors and, in turn, optimise the likelihood of successful implementation and potential for scale-up.

Ethical considerations

This research was approved by the Thai Central Research Ethics Committee (CREC) (COA-CREC020/2020), related university research ethics committees, and all hospital research ethics committees. Scientific and technical approval was obtained from the WHO Human Reproduction Programme (HRP) Review Panel on Research Projects (RP2), and ethical approval by the WHO Ethical Review Committee (protocol ID, 004571) and the French Research Institute for Sustainable Development. All participants provided written consent to participate and IDIs were conducted in a private place with no other people present.

Patient and public involvement

Patients and/or the public were not involved in the design, or conduct, or reporting, or dissemination plans of this research.

Results

From the eight participating hospitals, a total of 127 IDIs are included in this analysis: 27 pregnant women, 25 postpartum women, 16 potential companions, 8 facility administrators, 18 doctors and 33 nurse-midwives working in maternity care. Table 2 presents the sociodemographic characteristics of women and potential companions. Pregnant and postpartum women's ages ranged from 18 to 42 years, almost all were married or cohabitating with a partner, and most were employed. Among pregnant women, about half were nulliparous, including two women who had planned for a

For peer review only

For peer review only - http://bmjopen.bmj.com/site/about/guidelines.xhtml

caesarean birth. Among postpartum women, at their most recent birth, about one-third had a vaginal birth, and two-hirds had a caesarean birth. Almost all potential companions identified by the women were their husbands, except one who was the woman's mother. Table 3 presents the sociodemographic characteristics of healthcare providers. There were 12 men (doctors and administrators) and 59 women (14 female doctors/administrators; all nurse-midwives were women).

Contextual insights from the readiness assessment

Observations of the eight hospitals during the readiness assessment demonstrated space limitations and crowding on the labour ward, typically with multiple beds in the same room, close together and only divided by a curtain.

There are differences in current visiting hours in the labour and delivery wards across the hospitals. Two hospitals (hospitals 5 and 6) limit the visiting hours to three times a day, 1-2 hours in the morning, noon, and evening. In contrast, the five other hospitals allow visitors from 11 AM- 8 PM, but with limits on the number of visitors and duration of visits. Almost all hospitals allow only one visitor to visit for 15-20 minutes at a time. There is only one hospital (hospital 8) that allows woman who are in labour to visit the relatives at the ward reception area until 8 PM.

Two hospitals (hospitals 5 and 6) provide onsite overnight accommodation for relatives. One hospital (hospital 2) provides accommodation to the relatives only if the woman in labour is under 20 years old. In addition, two hospitals (hospitals 1 and 8) have a room for the relatives to be with the woman in labour until after the birth for extra charge.

Discussions between the research team and clinical staff as part of the readiness assessment suggested that a potential solution for seven hospitals would be to implement labour companionship for some, but not all women. For example, if seven women are in labour at the same time, labour companionship could be piloted with approximately two or three women without compromising care for all women. In these hospitals, it may be possible to make more private space for women during labour, for example by moving a woman who is in active labour to the corner of the ward and using curtains that are already available. Hospital 5 had serious concerns regarding the seriously limited space that might challenge the implementation of labour companionship.

Qualitative findings related to labour companionship

The findings showed that, in general, healthcare providers, women, and potential companions had positive attitudes about labour companionship. The qualitative findings are grouped in four

For peer review only

For peer review only - http://bmjopen.bmj.com/site/about/guidelines.xhtml

categories in the subsequent sections: 1) benefits of labour companions, 2) the roles of labour companions, 3) training for labour companions, and 4) factors affecting implementation.

1.0 Benefits of labour companions

Women, companions, and health workers expressed similar benefits and challenges of having labour companions, including 1) support, warmth and improved marital relationship, 2) having a representative to communicate with the medical team, 3) perception of clinical benefits, 4) labour companions as witnesses, 5) reduce the nursing workload in emotional support, and 6) labour companions may not be helpful. They have noticed the benefits of having the companion included shorten labour duration, to reduce caesarean section, to understand the work of medical team, to reduce the nurse-midwife's workload by being the woman's emotional supporter, and to provide opportunity for professional development. These benefits are outlined in the following sections. Recognition of the benefits of labour companionship are important facilitators for the *reflective* and *automatic motivation* domains of behaviour change, as they refer to the conscious thought processes (plans, evaluations) and habits or desires that influence motivation.

1.1 Support, warmth and improved marital relationship

Many women expressed that they feel anxious during the labour and birth. They feared the labour and birth process in the unfamiliar hospital environment. They experienced pain from contractions and worried about their safety and the baby's health. These women believed that having a companion might reduce fear and anxiety:

> *It is very nice to have some support. Some people need emotional support, wanting to have some familiar faces around. They looked around - they saw only the strangers. If they could see the mom or the husband, they would have felt some support that at least they have a friend. Having companions is very beneficial.*
>
> (Labour nurse-midwife 4, 7-years work experience, hospital 4)

> *It's good to have a companion…have someone to talk to while waiting…I would have felt relaxed…But if I were to have someone with me, I would have felt less anxious and forgot the pain a little bit.*
>
> (Postpartum woman 4, 35 years, hospital 4)

The participants from all groups said that having a labour companion present during the woman's labour and birth could improve the marital relationship if the husband was chosen to be a

For peer review only

For peer review only - http://bmjopen.bmj.com/site/about/guidelines.xhtml

companion. The husband and wife could go through the experience of the labour pain, emotional journey together.

> *Having my husband as a companion was very good. It's a very good bonding experience before the baby arrives. It's better for our family relationship.*

(Postpartum woman 10, 23 years, hospital 5)

> *One of the good things about having the companion is that we can support and consult with each other. We can go through it and help each other along the way.*

(Husband 1, 29 years, hospital 8)

1.2 Representative to the medical team

Participants described how labour pain can affect the woman's decision-making, perceptions, and judgment. Therefore, having a companion during labour who was a family member could be useful to act as a representative to communicate with the medical team. This can improve effective communication of the women's needs and preferences.

> *Many times, the patients are in so much pain. We couldn't really communicate with them…They couldn't make sound decisions. If they have a relative who can be their representative, it improves the communication and decision making.*

(Obstetrician 3, 10-years work experience, hospital 4)

> *Having a companion is a good thing. They can be my representative, if something is wrong. They can get a nurse for me.*

(Postpartum woman 4, 35 years, hospital 4)

1.3 Perception of clinical benefits

Some healthcare providers believed that if women had good support, they would be able to manage their pain which, in turn, seem to help shorten the labour duration.

> *One of the good things about having the companion is the smooth delivery…For example if the mom is with the patient, the mother might be able to support because the mother has experienced labour before. They can help the patient to follow the medical team's teaching like how to push correctly. The partner can help guide the patient to a successful labour.*

(Obstetrician-Administrator 2, 30-years work experience, hospital 4)

For peer review only - http://bmjopen.bmj.com/site/about/guidelines.xhtml

Moreover, some healthcare providers believed that when the women had good support, they may manage the pain better than if they did not have a companion. This could result in fewer caesarean births, as some women ask for caesarean because they no longer wish to tolerate the labour pain.

> *Having a companion with the woman seems to help with the surgery request* [for caesarean section]. *When the women are in labour pain, they will have someone with them to distract from the pain…. Many cases they ask for surgery because they are experiencing labour pain and don't want to wait until the natural delivery.*
>
> (Obstetrician 1, 11-years work experience, hospital 7)

1.4 Labour companions at witnesses

When the women's relatives stay with them throughout labour, they can witness the work of medical personals directly. Healthcare workers described that when family members are present, they tended to be more careful while working, which may therefore improve service quality.

> *It is like the companions are the quality assurance inspectors. They see how our system works. It is like a two-way communication that we can improve the quality of our service.*
>
> (Labour nurse-midwife 13, 34-years work experience, hospital 6)

Healthcare workers also felt the presence of companions could reduce some misunderstanding about medical malpractice, as the companion could witness and understand the work of the medical team which may lead to fewer lawsuits.

> *It's beneficial to have a labour companion. If there are any complications during the labour and the delivery, they will see that we try our best. When they see that we are trying the best we can, that might reduce the lawsuits. They have witnessed that we do pay attention. They can participate in the care.*
>
> (Obstetrician 2, 3-years work experience, hospital 7)

1.5 Reduce the nursing workload in emotional support

One of the nursing roles is providing emotional support to women during labour. The nurse-midwives also monitor frequency of contractions and provide other nursing care. When there are many women in labour, the nurse-midwives might not be able to provide close attention to every woman, and emotional support in particular can be compromised. Having a labour companion who has been trained on how to support women could therefore potentially reduce the nursing workload.

For peer review only

It helps reducing my workload.....I try to pay close attention to all my patients. I can do that when I have only a few patients. But when the patient has a labour companion, I feel good that my patients do receive intensive care, even though it's from the companion, not me.

(Labour nurse-midwife 14, 10-years work experience, hospital 6)

1.6 Labour companion may not be helpful

Most participants expressed the benefits of having labour companions. However, there were four women who said that they did not need a labour companion, primarily because they believed that during labour, nobody could help alleviate pain. These women believed that during labour, women tended to have limited attention and negative moods.

Either way is fine with me, having a companion or not. I am in labour. I will feel pain, no matter I have someone with me or not. Having a companion isn't helping with my pain.

(Pregnant woman 13, 31 years, hospital 5)

Moreover, one husband also said it was not helpful for him to be there. He said it is better for the woman to be with the medical team, and feared to see her suffer.

I think I will not be a labour companion. I will wait outside the room. I don't want to be in the way of the medical team. I am worried but I don't want to see her crying and suffering.

(Husband 3, 42 years, hospital 3)

2.0 The roles of labour companion

Most healthcare workers said that the women should be the one who select their labour companion. Most women preferred their husbands to be their labour companions, as they think that it will enhance the family relationship, and a few women preferred their mothers as they viewed their mothers' own labour experiences to be beneficial in supporting them. The participants from all groups expressed the roles of the labour companion very similarly, to provide emotional support, massage and support coping with pain, assisting with daily activities, and communicating with the medical team.

I would like someone who can be around and help out. Someone who holds me when I am in pain. Someone who can help getting things for me when I can't really help myself. It is better than being alone.

(Pregnant woman 10, 38 years, hospital 2)

For peer review only

The health workers also perceived that labour companions could play key roles in supporting them to better care for the women in labour.

> *The first thing is to be my support. Other duties can be understanding the labour and delivery process. So that person isn't in panic. If they notice any unusual symptoms, they can alert the medical team. They should have the ability to observe and report any abnormality. I see this person as a censor who detects problems.*
>
> (Obstetrician-Administrator 5, 20-years work experience, hospital 7)

> *I want to teach and train the companion. They should learn how to assess the labour pain, where they can check or touch. They will be the one who communicates with the nurses that the contraction is more frequent and intense. They can tell the nurses that the patient wants to push already.*
>
> (Labour nurse-midwife 15, 5-years work experience, hospital 6)

If labour companions were trained, for example during childbirth education classes or antenatal visits, these health workers believed that they could help the woman to manage pain, and communicate to the health workers if the woman needs help or is ready to push.

These critical roles played by labour companions are important facilitators to the *psychological capability* domain of behaviour change, which can influence the relationship between motivation and enacting the behaviour (labour companionship). If labour companions are appropriately equipped with the skills and knowledge to support women during labour, then they in turn have increased motivation, and health workers may feel better able to integrate them into the care team.

3.0 Training the labour companion

Participants expressed that potential labour companions should receive training to understand the process of labour and how to best support the woman. Preparation of the labour companions could be integrated into the existing antenatal classes. Most participants agreed that the training and preparation for the labour companion should start in the third trimester, approximately week 32 of the pregnancy. They should attend the class at least two times, for about 30-60 minutes. The key content and skills for labour companions to learn during these sessions is how to provide emotional support, pain management techniques, and understanding the process of labour. One female participant said that the labour companion should understand the emotions while the woman is going through labour pain so they can support the woman appropriately.

For peer review only - http://bmjopen.bmj.com/site/about/guidelines.xhtml

The labour companion has to learn how to support the patient. We should teach them what labour is and the pain associate to the labour, how much pain, when to report to the medical team. For instance, if the patient's water broke, they have to let us know. If the patient wants to push, they have to report.

(Obstetrician 2, 3-years work experience, hospital 7)

They have to learn the labour process. It will be somewhat a long process so they can help with the pain while waiting for the delivery. They can be a pushing coach. They have to be perceptive to our moods.

(Postpartum woman 24, 21 years, hospital 1)

The husband of a pregnant woman echoed the desire for learning how to support his wife, and particularly how he could help ease her pain during labour:

I want to learn what I should do, the process of getting on the labour and delivery wards, what to do when I am on the ward, how I can help my wife with the pain.

(Husband 8, 35 years, hospital 1)

Appropriate training of the labour companion is an important facilitator to the *physical* and *psychological capability* domains of behaviour change, which can increase *motivation*.

4.0 Factors affecting implementation

While all participants noted the many benefits to having a labour companion, some barriers and challenges to implementing companionship were identified. These factors affecting implementation are important barriers and facilitators to *physical* and *social opportunity*, as they relate to creating enabling physical environments and influencing positive sociocultural norms. Many labour and delivery wards in public hospitals are not designed to accommodate labour companions, as the wards are already crowded with women in labour. Consequently, four main barriers were identified by participants: 1) maintaining privacy and confidentiality, 2) increased risk of infection, 3) risk of lawsuits, and 4) perceived additional work for health workers to support companions. Maintaining privacy was already a challenge without labour companions, as the labour ward beds are close together, in a narrow and crowded room. In Thai culture, it is improper for women's bodies to be exposed; therefore, if a labour companion is a male, it may be uncomfortable for other women in labour at the same time.

For peer review only - http://bmjopen.bmj.com/site/about/guidelines.xhtml

Our hospital is a public hospital, not a private one. When the patients in labour, waiting to deliver, they are in their bed with a curtain as a divider between beds. There is no privacy. It's difficult for me to work and to protect my patients' privacy. For example, I am trying to do the pelvic exam but the next bed has a husband accompany her. The voices can travel through. It's difficult to work.

(Obstetrician 4, 2-years work experience, hospital 4)

In addition to the challenges of physical privacy, some participants also feared that having more visitors and relatives on the ward will be difficult for the medical team to protect the confidential information of patients.

I am very afraid of the risk of the confidentiality violation. The companions might talk about other patients to other people. I am very worried about this.

(Antenatal nurse-midwife 9, 21-years work experience, hospital 3)

Participants, particularly healthcare providers, expressed concerns about increased risks of infection, as the ward is usually crowded with women in labour. Adding the labour companion could lead to the increased risk of infection spread (referring to non-COVID-19 infection).

I think it's kind of risky for the infection. People wear their normal clothing, not sterile. That might increase the infection spread.

(Labour nurse-midwife 2, 3-years work experience, hospital 7)

Healthcare providers expressed concern that the presence of a labour companion may lead to misunderstanding and lawsuits. They worried that while they are providing care, the companions might think that the medical team are disorganized and in chaos, and that people may post these issues on social media. These misunderstandings and miscommunications had the potential to lead to lawsuits.

When I am on duty, I have to be more careful. My co-workers also warn me about this. For instance, I might be using my smartphone playing on my break but the relatives think I am not helping the patient who are yelling from pain. If they record and pose on social media, people see and misunderstand that I am not doing my job. Having a labour companion is like a two way sword. It has good and bad points.

(Labour nurse-midwife 14, 10-years work experience, hospital 6)

For peer review only

Lastly, many of the study hospitals had high ratios of women to healthcare providers, and healthcare providers feared that introducing companions to the ward may increase their workloads.

> *The objective of having a labour companion is to have someone to help us. But I doubt that the person can really help me. I have to explain and communicate more. It will double the communication times because I not only communicate with a patient, I have to communicate with the relatives.*
>
> (Labour nurse-midwife 6, 3-years work experience, hospital 2)

For successful implementation of companionship, these barriers would need to be considered and addressed in the implementation strategy. However, despite the barriers, the participants, particularly healthcare providers, believed that the potential benefits of introducing labour companionship would outweigh the risks, suggesting that labour companionship was highly acceptable.

> *I think it's possible to implement the labour companion policy because of the substantial benefits. It is easily acceptable. When there are many evidence-based research that show the benefits of having the labour companion can reduce the active and the second phase of the labour, they will change the policy and practice.*
>
> (Obstetrician 8, 3-years work experience, hospital 5)

> *If there is a policy to include the labour companion, I think it's possible to follow. They have to provide the space. When the direct order comes to the hospital to do it, they will set up more private space. I think it's possible. There shouldn't be any problems.*
>
> (Obstetrician-Administrator 1, 35-years work experience, hospital 7)

Understanding factors affecting implementation using the COM-B model

Figure 1 maps the potential factors affecting implementation from the qualitative interviews and readiness assessment to the COM-B model of behaviour change. The defined behaviour is that all women have the option to have a companion of their choice throughout labour and childbirth. In short, to improve *capability* to have a labour companion, potential labour companions should be well trained and prepared on how to support women throughout labour and birth, and measures may need to be taken to improve privacy. To improve *motivation* to have a labour companion, all stakeholders (women, potential companions, and healthcare providers) should be knowledgeable about the benefits of companions and how to efficiently integrate them into care, and trust-building between healthcare users and healthcare providers may need to take place in contexts with fear of

For peer review only

litigation. To improve *opportunity* to have a labour companion, labour wards may need to be physically reorganised to optimise space for a companion and woman to interact, revisions may be needed to allow consistent visitation rights for companions regardless of day or time, and facility or public policies may need revision to encourage companionship. To optimise the likelihood for this behaviour to occur in the QUALI-DEC hospitals in Thailand, the implementation strategies should ensure that the key barriers identified are addressed, and that the facilitators are present and encouraged in all sites.

Discussion

We found that healthcare providers, women, and potential companions in eight public hospitals in Thailand had generally positive attitudes towards having labour companions, and particularly belief that labour companions would provide beneficial psychological and physical support for the women. However, we identified some opportunities and threats to implementing labour companionship for all women. Training the labour companion, for instance through childbirth education classes or attendance at antenatal visits, was important to ensure that the companion knew how to support the woman and understood what to expect during labour and birth. Limited physical space on the labour wards, overcrowding, and multiple beds in the same labour room were major concerns to introducing labour companionship. While policies at the hospital and national level do not currently mention labour companionship, changes are more likely to be made at the hospital-level. For example, current restrictions on the timing of visitations and number of visitors allowed may challenge the implementation of labour companionship, and may need to be adjusted prior to implementation to ensure that companions are not subjected to visitor restrictions.

A key facilitator related to the social opportunities is that historically in Thai culture, childbirth occurred at home where the woman was surrounded by her family, and strong values and happiness in welcoming a new family member. Introducing labour companionship for births occurring in health facilities may therefore reflect the values and cultural appropriateness of having a woman's social network supporting her during labour and birth. While there are important barriers to address, namely around policies, training, and reorganisation of the physical environment for birth, social opportunities and psychological capabilities that value companionship are critical which appear to be present in Thai culture. These facilitators and barriers are remarkably similar to an implementation study conducted in public hospitals in Egypt, Lebanon, and Syria, where women and families highly valued companion support, but health workers identified critical organisational factors such as limited physical space, lack of training of companions, and limited policy engagement

For peer review only - http://bmjopen.bmj.com/site/about/guidelines.xhtml

For peer review only

as barriers to successful implementation (14, 15). The implementation study in Egypt, Lebanon, and Syria used participatory engagement through engagement with hospital leaders, seminars with healthcare providers, communications materials for companions, and changes to the physical space (chairs for companions, curtains around beds, access to hot water and toilets, and disposable gowns and nametags for companions) to address these barriers (14), which may also be a useful approach to inform the QUALI-DEC implementation.

Afulani and colleagues similarly explored women and health workers' perceptions of labour companionship in a public maternity unit in rural Kenya, and identified similar facilitators to labour companionship and roles that labour companions could play (16). In contrast to our findings, the Kenyan study identified additional social barriers, including women's belief that companions cannot help them, embarrassment to have a non-health worker see them during labour, and fears that the labour companion would gossip about what they saw during the birth to others or that the labour companion may abuse the woman during labour (16). While we did not identify these social barriers to implementation, it is possible that particularly the embarrassment and fears of gossip and abuse may be present in more rural areas of Thailand (all QUALI-DEC study hospitals are in urban areas and therefore may not be as influenced by these factors present in smaller communities).

Most women and companions believed a partner or husband to be the optimal companion, believing that witnessing the pain and supporting during the difficult time could strengthen the family bonding including the father and the baby, which was consistent with previous studies (4, 17). Only a few women preferred her mother as a companion. This finding is different from other women in India and Bangladesh, most those women wanted their mothers to be a companion (18, 19). Having a female companion, especially a mother, could yield other benefits, as they can share her own experiences of childbirth, which could serve as encouragement to women. We note that cultural and gender norms may influence the choice of a companion, and that ultimately the woman herself should be the person who chooses who will support her.

There are several key implications for research, practice, and implementation of the QUALI-DEC study. We plan to use opinion leaders (influential and respected healthcare leaders who are effective communicators, and identified by their colleagues or local authorities) at each study hospital to help support implementation (7). We plan to engage with the opinion leaders during an intensive, five-day pre-study training workshop, where we will present the results of this formative research and engage to design strategies to optimise implementation (7). Engaging with the opinion leaders about the benefits of labour companionship and co-designing strategies to address barriers to implementation that are feasible and acceptable in their clinical settings will be critical. For

For peer review only - http://bmjopen.bmj.com/site/about/guidelines.xhtml

example, we will explore how to assuage healthcare providers' fears that introducing companions will result in higher workloads, potentially through training solutions to help healthcare providers understand benefits of companions and how to integrate them in their care – a similar approach to Kabakian-Khasholian and colleagues (14). Similarly, we will discuss how to negotiate improving accountability of the health system to women and their families, with the potential risk that instances of poor quality of care are shared on social media by companions.

Moreover, we expect that at a minimum, some reorganisation of the physical space of the labour ward will be needed, for example introducing chairs, developing plans to mitigate the risk of overcrowding, and supplying curtains where necessary to enhance privacy. Likewise, some facility policies may need to be adjusted to change restrictions on visiting hours for the labour ward to ensure that companions are not subjected to visitor restrictions. More work will be needed to explore how to engage with labour companions during the antenatal period, and information, education, and communications (IEC) materials are underdevelopment to communicate how companions can support women and how health workers can engage them in care. The findings from this study have informed what type of material should be included in IEC materials for women and families, as well as health providers. For example, helping to clarify what to expect from a labour companion, how labour companions can help before, during, and after the birth, and practical information to help labour companions support women to the best of their abilities.

Our study had both limitations and strengths. While we aimed to include diverse public hospitals across different regions of Thailand, the findings may not be transferable to all settings in Thailand, including Southern Thailand where we could not include any hospitals. All study hospitals were in urban settings and generally hospitals with relatively high caesarean section rates, so there may be limited transferability to rural settings or settings with lower caesarean section rates. We collected the data during the COVID-19 pandemic, which may have introduced additional barriers to implementation around people's presence on the labour wards (during the data collection period July to October 2020, there were typically less than 10 COVID-19 cases per day in Thailand). We note that WHO COVID-19 clinical management guidance recommends that during the pandemic, all women should have access to woman-centred, respectful care, including a companion of their choice; this includes women with suspected, probably, or confirmed COVID-19 (20). Key strengths of our study include triangulation of results from qualitative research and the facility readiness assessment, and mapping of key factors affecting implementation to the COM-B model to guide decision-making during QUALI-DEC intervention design and support a systematic, targeted, and theory-based development of implementation strategies.

For peer review only - http://bmjopen.bmj.com/site/about/guidelines.xhtml

Conclusion

Labour companionship is viewed by women, potential companions, and health workers as highly beneficial and acceptable in the Thai context. If labour companions are well-trained on how to best support women, help them to manage pain, and engage with healthcare teams, it may be a feasible intervention to implement in the study hospitals. However, key barriers to introducing labour companionship must be addressed to maximise the likelihood of success. This includes changes to the physical environment in the labour ward to ensure that privacy can be adequately maintained and that there is space for companions to comfortably support women. Facility-level policies may need adjustment, particularly around visitation hours and where companions are not restricted. Context-specific solutions may need to be developed to assuage health worker concerns about potential misunderstandings, lawsuits, or reputational risks stemming from the introduction of labour companionship. Health workers will also need training to understand how to engage with labour companionships as part of a woman's care team, to minimise the risk of role encroachment and understand how companionship can be mutually beneficial. These key findings will be considered and deliberated on when developing the QUALI-DEC implementation strategies for introducing labour companionship.

For peer review only

For peer review only - http://bmjopen.bmj.com/site/about/guidelines.xhtml

Acknowledgements

We thank the woman, potential companions, and health workers for their valuable time, and are grateful to the social scientist team for their hard work in data collection: Nilubon Rujiraprasert, Sasitara Nuampa, Natthapat Buaboon, Waraluk Kittiwatanapaisan, and Dasavanh Bounmany. We are indebted to Miss Nampet Jampathong for her professionalism as a project coordinator during the data collection process and her assistance in data transcription.

Funding statement

This study is part of the QUALI-DEC-project which is co-funded by the European Union's Horizon 2020 research and innovation programme under grant agreement No 847567, and by UNDP-UNFPA-UNICEF-WHO-World Bank Special Programme of Research, Development and Research Training in Human Reproduction (HRP), a cosponsored programme executed by the World Health Organization (WHO) in the Department of Sexual and Reproductive Health and Research (SRH). The contents of this article are solely the responsibility of the authors and do not reflect the views of the EU, UNDP, UNFPA, UNICEF, WHO or the World Bank.

For peer review only

Contributorship statement

SR, AR, PL, CH, AD, MdL, APB and MAB designed the study. SR, AR, and PL led data collection with support from CH, AD, MdL, APB and MAB. SR led data analysis with support from RZ and MAB. SR and MAB drafted the manuscript, and all authors reviewed the manuscript.

Data sharing statement

No additional data available.

Patient and public involvement

Patients and/or the public were not involved in the design, or conduct, or reporting, or dissemination plans of this research.

Competing Interests

None declared.

For peer review only - http://bmjopen.bmj.com/site/about/guidelines.xhtml

For peer review only

For peer review only - http://bmjopen.bmj.com/site/about/guidelines.xhtml

References

1. Tunçalp Ö, Were WM, MacLennan C, Oladapo OT, Gülmezoglu AM, Bahl R, et al. Quality of care for pregnant women and newborns-the WHO vision. Bjog. 2015;122(8):1045-9.

2. World Health Organization. Companion of choice during labour and childbirth for improved quality of care. World Health Organization; 2020 2020.

3. World Health Organization. WHO recommendations: intrapartum care for a positive childbirth experience. World Health Organization; 2018 2018. Report No.: 978-92-4-155021-5.

4. Bohren MA, Berger BO, Munthe-Kaas H, Tunçalp Ö. Perceptions and experiences of labour companionship: a qualitative evidence synthesis. The Cochrane database of systematic reviews. 2019;3(3):Cd012449.

5. Bohren MA, Hofmeyr GJ, Sakala C, Fukuzawa RK, Cuthbert A. Continuous support for women during childbirth. The Cochrane database of systematic reviews. 2017;7(7):Cd003766.

6. Jaroensook C. Effects of Labor Support by a Female Relative on Suffering from Labor Pain, Coping with Labor Pain, and Satisfaction with Childbirth Experiences Among Primiparous Parturients. The Journal of Faculty of Nursing Burapha University. 2019;26(2):67-75.

7. Dumont A, Betrán AP, Kaboré C, de Loenzien M, Lumbiganon P, Bohren MA, et al. Implementation and evaluation of nonclinical interventions for appropriate use of cesarean section in low- and middle-income countries: protocol for a multisite hybrid effectiveness-implementation type III trial. Implementation Science. 2020;15(1):72.

8. Balde MD, Nasiri K, Mehrtash H, Soumah A-M, Bohren MA, Diallo BA, et al. Labour companionship and women's experiences of mistreatment during childbirth: results from a multi-country community-based survey. BMJ global health. 2020;5(Suppl 2):e003564.

9. Singh S, Goel R, Gogoi A, Caleb-Varkey L, Manoranjini M, Ravi T, et al. Presence of birth companion—a deterrent to disrespectful behaviours towards women during delivery: an exploratory mixed-method study in 18 public hospitals of India. Health Policy and Planning. 2021;36(10):1552-61.

10. Bohren MA, Opiyo N, Kingdon C, Downe S, Betrán AP. Optimising the use of caesarean section: a generic formative research protocol for implementation preparation. Reproductive health. 2019;16(1):170.

11. Tong A, Sainsbury P, Craig J. Consolidated criteria for reporting qualitative research (COREQ): a 32-item checklist for interviews and focus groups. International Journal for Quality in Health Care. 2007;19(6):349-57.

12. Braun V, Clarke V. Using thematic analysis in psychology. Qualitative Research in Psychology. 2006;3(2):77-101.

13. Michie S, Van Stralen MM, West R. The behaviour change wheel: a new method for characterising and designing behaviour change interventions. Implementation science. 2011;6(1):42.

14. Kabakian-Khasholian T, Bashour H, El-Nemer A, Kharouf M, Elsheikh O, the Labour Companionship Study G. Implementation of a labour companionship model in three public hospitals in Arab middle-income countries. Acta Paediatrica. 2018;107(S471):35-43.

15. Kabakian-Khasholian T, El-Nemer A, Bashour H. Perceptions about labor companionship at public teaching hospitals in three Arab countries. International journal of gynaecology and obstetrics: the official organ of the International Federation of Gynaecology and Obstetrics. 2015;129(3):223-6.

16. Afulani P, Kusi C, Kirumbi L, Walker D. Companionship during facility-based childbirth: results from a mixed-methods study with recently delivered women and providers in Kenya. BMC pregnancy and childbirth. 2018;18(1):150-.

17. Kabakian-Khasholian T, Portela A. Companion of choice at birth: factors affecting implementation. BMC Pregnancy and Childbirth. 2017;17(1):265.

18. Bangal V, Bayaskar V, Arjun A, Khan I, Thorat U. Opinions of pregnant women regarding desire and choice of labour companion. Women's Health. 2018;7.

For peer review only - http://bmjopen.bmj.com/site/about/guidelines.xhtml

19. Perkins J, Rahman AE, Mhajabin S, Siddique AB, Mazumder T, Haider MR, et al. Humanised childbirth: the status of emotional support of women in rural Bangladesh. Sexual and Reproductive Health Matters. 2019;27(1):228-47.

20. World Health Organization. Clinical management of COVID-19 patients: Living guidance. Geneva, Switzerland: World Health Organization; 2021.

For peer review only

For peer review only - http://bmjopen.bmj.com/site/about/guidelines.xhtml

Figures and Tables

Table 1. Study sites in Thailand

Hospital #	Region	Type of hospital	# of births per year (2020)	Caesarean section rate (2020)
Hospital 1	Central Thailand	Public hospital	4,431	43.6%
Hospital 2	Central Thailand	Public hospital	4,605	34.3%
Hospital 3	Central Thailand	Public teaching hospital	5,203	48.5%
Hospital 4	Northeast Thailand	Public teaching hospital	1,727	42.5%
Hospital 5	Northeast Thailand	Public hospital	4,756	43.6%
Hospital 6	Northeast Thailand	Public hospital	3,361	49.2%
Hospital 7	Northern Thailand	Public hospital	5,025	50.1%
Hospital 8	Eastern Thailand	Public hospital	3,268	56.9%

For peer review only

For peer review only - http://bmjopen.bmj.com/site/about/guidelines.xhtml

Table 2. Sociodemographic of participants: women and potential companions

	Pregnant women	Postpartum women	Potential companions
**Total number of participants**	27	25	16
**Age (years)**			
18-24	8	4	0
25-30	9	9	4
31-42	10	12	10
43-59	0	0	2
**Marital status**			
Single	0	0	0
Married/cohabitating	26	25	15
Divorced/widowed	1	0	1
**Occupation**			
Government officer	3	2	0
Business owner	8	5	5
Employed (other)	8	11	10
Unemployed	8	7	1
**Parity and planned mode of birth**			
Nulliparous (no planned CS)	10	-	-
Nulliparous (planned CS)	2	-	-
Multiparous (no planned CS)	9	-	-
Multiparous (planned CS)	6	-	-
**Mode of birth (most recent birth)**			
Vaginal birth	-	8	-
CS	-	17	-

CS: caesarean section

For peer review only

For peer review only - http://bmjopen.bmj.com/site/about/guidelines.xhtml

Table 3. Sociodemographic of participants: health care providers

	Administrators	Doctors	Nurse-midwives
**Total number of participants**	8	18	33
**Gender**			
Female	2	12	33
Male	6	6	0
**Years working in total**			
1-5	0	7	8
6-10	0	5	2
11-15	0	2	1
16-20	1	2	5
21-25	0	1	5
26-30	4	1	4
≥ 31	3	0	8
**Years working at study facility**			
1-5	0	11	10
6-10	0	1	5
11-15	0	3	4
16-20	1	2	4
21-25	0	0	4
26-30	4	1	3
≥ 31	3	0	3

For peer review only

Figure 1. Mapping the factors affecting implementation of labour companionship in Thailand to the COM-B model of behaviour change. This figure maps the factors affecting labour companionship from the qualitative research findings and readiness assessment to the COM-B model of behaviour change. The COM-B model is a useful way to identify what changes need to occur for an intervention – such as companionship – to be effective. Developing implementation strategies that capitalise on the facilitators and address the barriers to capability, opportunity, and motivation is a critical next step for the QUALI-DEC project.

[insert figure 1 here]

Footnote to figure 1:

Data coming from: * = women, ¥ = labour companion, † = doctors, ‡ = nurse-midwives, R = readiness assessment

For peer review only

For peer review only - http://bmjopen.bmj.com/site/about/guidelines.xhtml

Capability to have a labour companion

Physical

Facilitators (*for labour companionship to be implemented*):

Labour companions should have sufficient time to assist woman throughout labour*
Labour companions should be able to join parenting classes or antenatal visits ‡
Labour companions should receive training on how to support women during labour and birth in parenting class or antenatal visit*ɣ†‡
Some facilities have piloted labour companionship successfully†‡

Barriers:

No national policies include labour companionship†‡
Inability to maintain privacy of women†‡

Psychological

Facilitators (*for labour companionship to be implemented*):

Labour companion, women and health workers should be aware the benefits of labour companionship*ɣ†‡
Women and health workers should be aware that women can have someone to support them during labour*†‡
Labour companions should understand labour process*†‡
Labour companions should understand women's pain and how to support them*
Labour companions should know how to massage and other measures to support women to cope with pain*ɣ
Labour companions should can provide emotional support to women*ɣ
Labour companions should know how to assist with daily activities while at the facility*
Labour companions should know how to and are comfortable to communicate with medical team*
Labour companions should **have previous experience** of labour or labour companionship*
Labour companions should have a family relation of the woman, most preferred husband or mother*
Labour companions should help ease health worker workload by providing emotional support to women†‡
Labour companions should can help identify when women need additional help from health workers†‡

Barriers:

Labour companion **has no previous experience** in labour or labour companionship*ɣ

Motivation to have a labour companion

Reflective

Facilitators:

Participants beliefs that labour companion will:

Have positive clinical effects: shorten labour duration, reduction of caesarean section, professional development†‡
Improve understanding between family and medical team†‡
Reduce nurses' workload in providing emotional support to women‡
Represent and communicate women's needs*ɣ†‡
Improve pain management of women†‡
Be a quality assurance inspector†‡

Barriers:

Fear of increased infection risk from labour companion's presence on labour ward*†‡
Fear of misunderstandings and lawsuits†‡
Fear of increased workload for health workers†‡

Automatic

Facilitators:

Belief of positive benefits of labour companionship*ɣ†‡
Belief that labour companion will reduce women's stress, loneliness and pain during labour*ɣ†‡
Belief that labour companion's presence will strengthen marital relationship*ɣ†‡
Women feel supported, relaxed and warmth*ɣ†‡

Barriers:

Labour companion fear of seeing women sufferingɣ
Perception that labour companion is not helpful*ɣ

Opportunity to have a labour companion

Physical

Facilitators:

When labour occurs in private room with no others present[R]*ɣ†‡
Some facilities have 'premium' suites that allow for labour companionship[R]†‡
Labour space management for labour to ensure privacy, i.e. corner of the room[R]‡

Barriers:

Space limitations in labour ward*[R]†‡
Overcrowding of labour ward*[R]†‡
Privacy in labour ward is by curtains between beds which may be insufficient for maintaining privacy†‡
No accommodation available for labour companion[R]
Current restrictions on timing and number of visitations[R]
Potential labour companions may not have time availability to participate in training as a companion - may not know how to best support women[R]*ɣ

Social

Facilitators:

Thai culture promotes family support during labour [R]
Opportunity for local action and planning to implement labour companionship[R]†‡

Barriers:

May not be appropriate for women to have men who are not health workers see their bodies*†‡
No national policies support labour companionship†‡

Behaviour:

All women have the option to have a companion of their choice throughout labour and childbirth

Appendix 1. In-depth interview guides

This file contains the in-depth interview guides for all participant groups in this study:

1. Pregnant women (page 2)
2. Postpartum women (page 6)
3. Potential companions before birth (page 11)
4. Potential companions after birth (page 15)
5. Health workers (page 19)

For peer review only

For peer review only - http://bmjopen.bmj.com/site/about/guidelines.xhtml

In-depth interview: pregnant women

Step 1: Introduce yourself to the participant. Describe the purpose of the interview and how information will be used. Obtain oral/written consent.
Step 2: Ask the participant to identify herself. Interviewer: fill out the information below prior to beginning the interview.
Step 3: Conduct the interview. Please remember to audio record the interview.

Interview place: ______________________________

Interview date: ______________________________

Start time: ______________________________

End time: ______________________________

Interviewer name: ______________________________

Interview identification: ______________________________

Participant information

Duration of current pregnancy (weeks/months, please label):______________________________

Age (years): ______________________________

Marital status (Single, married/cohabitating, divorced, widowed): ____________________

Occupation (write in): ______________________________

For peer review only

Part 1. Values and needs surrounding the childbirth period

1. What are some things you are doing to prepare for your birth?
    a. Probe: who is helping you to prepare? How are they helping?

2. Thinking about your pregnancy, what are some of the things you value most? Why?
    a. Probe: to keep yourself healthy?
    b. Probe: to keep your baby healthy?

3. Thinking to the future about your birth, what are some of the things that are most important to you?
    a. Probe: What will you need from your doctors and midwives to make sure this happens?
    b. Probe: what will you need from your family to make sure this happens?
    c. Where are you planning to give birth? Why?
        i. Did anyone help you decide where to give birth? Who? How did they help?

Part 2. Prenatal education

4. Thinking about when you go to your antenatal care visits, what are some of the things that are most important to you to learn about?
    a. Probe: What are some of the most important things you have learned during antenatal care? Why are they important?
    b. Probe: How do you think antenatal care could be improved?
        i. What do you think is missing from your antenatal care visits?
        ii. Are there any things that you would remove or change during your antenatal care visits?

Part 3. Preferences and decision-making processes regarding mode of childbirth

5. Could you tell me about the different ways that women can give birth?
    a. Probe: How did you learn about these options?

6. What do you think about vaginal birth and caesarean section?

7. What do you think are some of the positive things about vaginal birth?
    a. Probe: Why are these positive things?
    b. Probe: How did you learn about these positive things?

8. What are some of the negative things about vaginal birth?
    a. Probe: Why are these negative things?
    b. Probe: How did you learn about these negative things?

9. What do you think are some of the positive things about caesarean section?
    a. Probe: Why are these positive things?
    b. Probe: How did you learn about these positive things?

10. What are some of the negative things about caesarean section?
    a. Probe: Why are these negative things?
    b. Probe: How did you learn about these negative things?

11. How did you learn about vaginal birth and caesarean section?

For peer review only - http://bmjopen.bmj.com/site/about/guidelines.xhtml
For peer review only

12. How would you prefer to give birth? For example, caesarean or vaginal birth?
    a. Why do you prefer to give birth this way?
    b. How important is it for you to give birth this way? Why?
    c. Was anyone involved in helping you make a decision about how you prefer to give birth?
        i. Probe: Will your (husband/partner) influence this decision? How?
        ii. Probe: Will your family influence this decision? How?
        iii. Probe: Will your friends influence this decision? How?
        iv. Probe: Will your doctor or midwife influence this decision? How?
        v. Probe: Will the media influence this decision? How?

13. Do you feel like you have enough information to understand the options that you have for how to give birth? Why or why not?
    a. Probe: What other type of information about different modes of childbirth would you be interested to learn about?
    b. At what point during your pregnancy would you like to receive this information?

14. Did you discuss your preference with your doctor or midwife? If so, what was the discussion like?

15. *A decision-analysis tool could help to educate women about their options for mode of birth and how to discuss their preferences with a doctor. Interviewer: show the woman the Vietnam decision analysis tool.*
    a. Would this type of tool be helpful to you? Why or why not?
    b. What type of information would you like to have included?
    c. *This type of decision tool can be paper based like this example, or could be an application for a phone*. Which of these options do you prefer and why?

16. Pregnancy and childbirth are exciting times but can also be scary for some women. Is there anything that you are afraid or nervous of during your pregnancy? Why or why not?
    Note to interviewer: if they bring up fear of pain, then probe about what pain management technique they have learned about.

    a. What about during your birth, is there anything that you are afraid of? Why or why not?
        i. Have you spoken to your doctor or midwife about these fears? Why or why not? What did they tell you?
        ii. Have you spoken to anyone else about these fears?
            1. *If yes*:
                a. Who did you speak to? Why did you choose to speak to this person?
                b. What type of advice did they give you?
            2. *If no*: Do you plan to speak to anyone about these fears? Why or why not?
        iii. What do you think could be done to help reduce this fear for you?

Part 4. Labour companionship

17. What do you need in order to have a positive experience when you go to the hospital for childbirth?
    d. What type of support do you think you need during labour and childbirth?

For peer review only - http://bmjopen.bmj.com/site/about/guidelines.xhtml

Interviewer read: A labour companion is a person of the woman's choice, who can help to provide emotional support to the woman during labour and childbirth. Typically, this person would be with the woman continuously throughout labour and childbirth. This person may be the woman's husband/partner, her mother, or a friend.

18. Do you think you will receive this type of support? Why or why not?

19. Have you ever heard of someone providing this type of support?

20. What do you think of this type of support?

21. Do you know if labour companionship is allowed in the hospital you plan to give birth in?
    - e. *If labour companionship is not allowed*: What do you think are the reasons for not allowing a labour companion?
    - f. Would you be allowed a labour companion if you requested it? Why or why not?
    - g. In your opinion, what changes do you think the hospital could make in order to make it more comfortable for women to have a labour companion?

22. Do you think you would want to have a labour companion for your upcoming birth? Why or why not?
    - h. What type of information or education would YOU need before deciding if you wanted to have a labour companion to support you?

23. If you were to have a labour companion with you:
    - i. What would you expect from this person?
    - j. When would you want this person to be with you in the hospital (probe: the whole time, only during labour but not during the birth, something else?)
    - k. Who would you prefer this person to be? Why?
    - l. When would you like to start talking to your labour companion about their role during your labour and childbirth?
  - iii. Probe: at what month during your pregnancy?

24. What type of information or education do you think a labour companion would need to be able to support you?

Thank you so much for your time. Is there anything else that you would like to share with me today about anything we talked about?

For peer review only

For peer review only - http://bmjopen.bmj.com/site/about/guidelines.xhtml

In-depth interview: postpartum women

Step 1: Introduce yourself to the participant. Describe the purpose of the interview and how information will be used. Obtain oral/written consent.
Step 2: Ask the participant to identify herself. Interviewer: fill out the information below prior to beginning the interview.
Step 3: Conduct the interview. Please remember to audio record the interview.

Interview place: ________________________________

Interview date: _________________________________

Start time: _____________________________________

End time: ______________________________________

Interviewer name: _________________________________

Interview identification: ___________________________________

<u>Participant information</u>

Duration of current pregnancy (weeks/months, please label):_________________________________

Age (years): ____

Marital status (Single, married/cohabitating, divorced, widowed): _____________________

Occupation (write in): ____

For peer review only

For peer review only - http://bmjopen.bmj.com/site/about/guidelines.xhtml

Part 1. Values and needs surrounding the childbirth period

25. Can you tell me about your recent birth?
    a. Where did you give birth?
    b. Were you planning to give birth there? Why or why not?
    c. How did you make the decision about where to give birth?
        i. Who was involved in the decision about where to give birth?
        ii. Probe: Did you influence this decision? How?
        iii. Probe: Did your family or her family influence this decision? How?
        iv. Probe: Did your friends or her friends influence this decision? How?
        v. Probe: Did the doctor or midwife influence this decision? How?
        vi. Probe: Did the media influence this decision? How?

26. How did you feel about your overall experience of giving birth?
    a. How would you describe your birth experience? Why?

27. Satisfaction
    a. How satisfied are you with the type of care you received during labour and childbirth?
    b. Can you give me an example of something you are very satisfied with?
    c. Can you give me an example of something you are NOT satisfied with?

28. How well do you feel that your healthcare provider respected your opinions about care during labour and childbirth?
    a. Can you give me an example of a time when your healthcare provider respected your opinions about your care during labour and childbirth?
        i. How did this make you feel?
    b. Can you give me an example of a time when your healthcare provider did NOT respect your opinions about your care during labour and childbirth?
        i. Probe: Or, an example of a time during labour and childbirth when someone else was making decision without talking with you?
            1. How did this make you feel?

Part 2. Decision-making processes regarding mode of childbirth

29. Could you tell me about the different ways that women can give birth?
    a. Probe: How did you learn about these options?

30. What about for your most recent birth – how did you give birth? For example, caesarean or vaginal birth?
    a. Is this the way that you preferred to give birth? Why or why not?
    b. Probe: How did you come to give birth in this way?
        i. Probe: Did your (husband/partner) influence this decision? How?
        ii. Probe: Did your family influence this decision? How?
        iii. Probe: Did your friends influence this decision? How?
        iv. Probe: Did your doctor or midwife influence this decision? How?
            1. Did you discuss this decision with your doctor or midwife? If so, what was the discussion like?
        v. Probe: Did the media influence this decision? How?

31. What do you think about vaginal birth and caesarean section?
    a. What do you think are some of the positive things about vaginal birth?

For peer review only - http://bmjopen.bmj.com/site/about/guidelines.xhtml

i. Probe: Why are these positive things?
ii. Probe: How did you learn about these positive things?

b. What are some of the negative things about vaginal birth?
   i. Probe: Why are these positive things?
   ii. Probe: How did you learn about these positive things?
c. What do you think are some of the positive things about caesarean section?
   i. Probe: Why are these positive things?
   ii. Probe: How did you learn about these positive things?
d. What are some of the negative things about caesarean section?
   i. Probe: Why are these positive things?
   ii. Probe: How did you learn about these positive things?

32. How did you learn about vaginal birth and caesarean section?

33. Did you feel like you had enough information to understand the options that you had for how to give birth? Why or why not?
    a. Probe: What other type of information about different modes of childbirth would you have been interested to learn about?
    b. At what point during your pregnancy would you like to receive this information?

Part 3. Prenatal education

34. Thinking back to your antenatal care visits, what were some of the things that were most important to you to learn about?
    a. Probe: What are some of the most important things you have learned during antenatal care? Why are they important?
    b. Probe: How do you think antenatal care could be improved?
       i. What do you think was missing from your antenatal care visits?
       ii. Are there any things that you would remove or change during your antenatal care visits?

Part 4. Decision-aids

35. Where did you get most of the information to educate you about what to expect while giving birth?
    a. What type of topics did you learn about?
    b. How well do you feel these educational materials prepared you to give birth? Why?
       i. Probe: can you give me an example of something that you felt very well prepared for?
       ii. Probe: can you give me an example of something that you did NOT feel well prepared for?
    c. Overall, how well prepared did you feel to give birth? Why?

For peer review only - http://bmjopen.bmj.com/site/about/guidelines.xhtml

36. Did you feel like you had sufficient time to talk to your doctor or midwife about any concerns that you had about labour and childbirth? Why or why not?
    a. Can you give me an example of a time when you felt that you were able to discuss your questions or concerns with your doctor or midwife?
    b. Can you give me an example of a time when you felt that you were NOT able to discuss your questions or concerns with your doctor or midwife?

37. *A decision-analysis tool could help to educate women about their options for mode of birth and how to discuss their preferences with a doctor.* Interviewer: show the woman the Vietnam decision analysis tool.
    a. Would this type of tool be helpful to you? Why or why not?
    b. What type of information would you like to have included?
    c. This type of decision tool can be paper based like this example, or could be an application for a phone. Which of these options do you prefer and why?

Part 5. Labour companionship

38. Who was with you while you were in labour?
    a. Probe: was your husband/partner with you?
        i. *If yes*:
            1. What was he doing while you were in labour and giving birth?
            2. Was he in the room with you? Why or why not?

    b. Probe: were any of your family members or friends with you?
        i. *If yes*:
            1. Who was with you?
            2. What were they doing while you were in labour and giving birth?
            3. Were they in the room with you? Why or why not?

39. What type of support do you think that you need during labour and childbirth while in the facility?
    a. Did you feel that you were supported during labour and childbirth? Why or why not?
    b. Can you give me an example of when you did feel supported?
    c. Can you give me an example of when you did not feel supported?
    d. What could have been done to improve your experience of support during labour and childbirth?
        i. Probe: Why do you think this is important?

Interviewer to read: Some women have a person with them during labour and childbirth, and we call this person a "labour companion". A labour companion is typically a woman's husband, boyfriend, sister, mother, or friend, who stays with the woman throughout labour and childbirth. They help the woman by providing emotional support, praising her and reassuring her.

40. What do you think about this type of support?

41. Would you have wanted someone to support you in this way during your labour and childbirth? Why or why not?

For peer review only - http://bmjopen.bmj.com/site/about/guidelines.xhtml

42. Who would you want this person to be?
    a. Probe: your husband/partner? Why?
    b. Probe: a sister or friend? Why?
    c. Probe: a mother or mother-in-law? Why?

43. When would you want to have this person with you?
    a. Probe: all of the time during labour and childbirth?
    b. Probe: only some of the time (e.g. only during labour, but not the birth)

44. How do you think having a labour companion might be helpful?

45. What are some challenges to having a labour companion?

46. Do you know if labour companionship is allowed in the hospital you gave birth in?
    a. *If labour companionship is not allowed:* What do you think are the reasons for not allowing a labour companion in this hospital?

47. What changes do you think the hospital could make to make it more comfortable for women to have a labour companion?

48. Do you have any other comments or feedback about labour companionship?

Thank you so much for your time. Is there anything else that you would like to share with me today about anything we talked about?

For peer review only - http://bmjopen.bmj.com/site/about/guidelines.xhtml

In-depth interview: partner / potential companion (before birth)

Step 1: Introduce yourself to the participant. Describe the purpose of the interview and how information will be used. Obtain oral/written consent.
Step 2: Ask the participant to identify herself. Interviewer: fill out the information below prior to beginning the interview.
Step 3: Conduct the interview. Please remember to audio record the interview.

Interview place: ________________________________

Interview date: _________________________________

Start time: _____________________________________

End time: ______________________________________

Interviewer name: _________________________________

Interview identification: ____________________________

<u>Participant information</u>

Relationship with pregnant woman: _________________________________

Age (years): ____

Marital status (Single, married/cohabitating, divorced, widowed): _____________________

Occupation (write in): ____

<u>Information on pregnant women</u>

N° of women's interview: ___________________________________

Duration of current pregnancy:______________________________

For peer review only - http://bmjopen.bmj.com/site/about/guidelines.xhtml

Part 1. Values and needs surrounding the childbirth period

49. What are some things you are doing to prepare for your (wife/partner/daughter/sister…) birth?
    a. Probe: who is helping you to prepare? How are they helping?

50. Thinking about your wife/partner/sister pregnancy, what are some of the things you value most? Why?
    a. Probe: to keep her healthy?
    b. Probe: to keep the baby healthy?
    c. Probe: for yourself personally?

51. Thinking to the future about your (wife/partner/daughter/sister…) birth, what are some of the things that are most important to you?
    a. Probe: What will you need from your doctors and midwives to make sure this happens?
    b. Probe: what will you need from your family to make sure this happens?

52. Place of birth
    a. Where is your (wife/partner/daughter/sister…) planning to give birth? Why?
    b. How did she make the decision about where to give birth?
        i. Did anyone help her make the decision about where to give birth? Who? How?

Part 2. Decision-making processes regarding mode of childbirth

53. Could you tell me about the different ways that women can give birth?
    a. Probe: How did you learn about these options?

54. What do you think about vaginal birth and caesarean section?
    a. What do you think are some of the positive things about vaginal birth?
    b. What are some of the negative things about vaginal birth?
    c. What do you think are some of the positive things about caesarean section?
    d. What are some of the negative things about caesarean section?

55. How would you prefer that your (wife/partner/daughter/sister…) gives birth? For example, caesarean or vaginal birth?
    a. Probe: Why do you prefer this way?
    b. Does your (wife/partner/daughter/sister…) also prefer to give birth this way? Why or why not?

56. Did your (wife/partner/daughter/sister…) decide about how she will give birth? For example, caesarean or vaginal birth?
    a. Probe: Who makes this decision?
    (note: the decision about mode of birth has not yet been made, or if he/she responds that they don't know to question 7, please instead ask: "How do you think she would plan to make this decision?")
        i. Probe: Did you influence this decision? How?
        ii. Probe: Did your family or her family influence this decision? How?
        iii. Probe: Did your friends or her friends influence this decision? How?
        iv. Probe: Did the doctor or midwife influence this decision? How?
        v. Probe: Did the media influence this decision? How?

For peer review only - http://bmjopen.bmj.com/site/about/guidelines.xhtml

Part 3. Prenatal education

57. Have you been to any antenatal care visits with your (wife/partner/daughter/sister…) ? Why or why not?
    a. *If yes:* Thinking about when you went to the antenatal care visits, what are some of the things that are most important to you?
        vi. Probe: What are some of the most important things you have learned during antenatal care? Why are they important?
        vii. Probe: How do you think antenatal care could be improved?
        viii. What do you think is missing from the antenatal care visits?
        ix. Are there any things that you would remove or change during the antenatal care visits? What are they and why would you change?

58. Thinking about antenatal care visits, what are some of the things that are most important to you to learn about?
    a. Probe: What are some of the most important things you have learned during antenatal care? Why are they important?
    b. Probe: How do you think antenatal care could be improved?

Part 4. Decision-aids

59. How did you learn about vaginal birth and caesarean section?

60. Do you feel like you have enough information to understand the options that women have for how to give birth? Why or why not?
    i. Probe: What other type of information about different modes of childbirth would you be interested to learn about?
    ii. At what point during your (wife/partner/sister…'s) pregnancy would you like to receive this information?

61. Pregnancy and childbirth are exciting times but can also be scary. Is there anything that you are afraid of or nervous about pregnancy or childbirth? Why or why not?
    a. What about during the birth, is there anything that you are afraid of? Why or why not?
    b. *If yes*:
        i. Have you spoken to anyone about these fears? Why or why not?
            1. *If yes*: What did they tell you?
            2. *If no*: Do you plan to speak to anyone about these fears? Why or why not?
        ii. What do you think could be done to help reduce this fear for you?

Part 5. Labour companionship

62. Do you plan to go to the hospital with your (wife/partner/daughter/sister…) when she gives birth? Why or why not?

63. If you do go to the hospital when your (wife/partner/ daughter/sister…) gives birth, what do you need in order to have a positive experience?

64. What type of support do you think your (wife/partner/ daughter/sister…) needs during labour and childbirth?
    a. Do you think she will receive this type of support? Why or why not?

For peer review only - http://bmjopen.bmj.com/site/about/guidelines.xhtml

Interviewer read: A labour companion is a person of the woman's choice, who can help to provide emotional support to the woman during labour and childbirth. Typically, this person would be with the woman continuously throughout labour and childbirth. This person may be the woman's husband/partner, her mother, or a friend.

65. Have you ever heard of someone providing this type of support?

66. What do you think of this type of support?

67. Do you know if labour companionship is allowed in the hospital your (wife/partner/ daughter/sister…) plan to give birth in?
    a. *If labour companionship is not allowed*: What do you think are the reasons for not allowing a labour companion?
    b. Would your (wife/partner/sister…) be allowed a labour companion if she requested it? Why or why not?

68. Have you ever provided this type of support before? (*If yes*: Could you tell me more about this?)

69. Do you think your (wife/partner/ daughter/sister…) would want to have a labour companion for her upcoming birth? Why or why not?

70. If your (wife/partner/ daughter/sister…) were to have a labour companion with her, who do you think she would prefer this person to be? Why?

71. *If your (wife/partner/ daughter/sister…) were to have a labour companion with her, what do you think she would expect from this person?*

72. *Would you be interested in being a labour companion to your (wife/partner/ daughter/sister…)? Why or why not?*

73. What would you need in order to be a good labor companion?
    a. What do you need from the woman?
    b. What do you need from the nurses and doctors?
    c. What do you need from the hospital?

74. What type of information or education do you think a labour companion would need to be able to support her?
    a. When during pregnancy do you think a woman or a nurse should start talking to a potential labour companion about their role during labour and childbirth?

75. In your opinion, what changes do you think the hospital could make in order to make it more comfortable for women to have a labour companion?

Thank you so much for your time. Is there anything else that you would like to share with me today about anything we talked about?

For peer review only - http://bmjopen.bmj.com/site/about/guidelines.xhtml

In-depth interview: partner / potential companion (postpartum)

Step 1: Introduce yourself to the participant. Describe the purpose of the interview and how information will be used. Obtain oral/written consent.
Step 2: Ask the participant to identify herself. Interviewer: fill out the information below prior to beginning the interview.
Step 2: Conduct the interview. Please remember to audio record the interview.

Interview place: ________________________________

Interview date: _________________________________

Start time: _____________________________________

End time: ______________________________________

Interviewer name: __________________________________

Interview identification: ____________________________________

Participant information

Relationship with pregnant woman: _________________________________

Age (years): ____

Marital status (Single, married/cohabitating, divorced, widowed): _____________________

Occupation (write in): ____

Information on pregnant women

N° of women's interview: ___________________________________

Woman's date of most recent birth:_________________________________

For peer review only - http://bmjopen.bmj.com/site/about/guidelines.xhtml
For peer review only

Part 1. Values and needs surrounding the childbirth period

Note to interviewers: only ask questions 1-3 at Khon Kaen University Hospital or any hospital that allows companions.

76. How did you feel about your overall experience of your (wife/partner/daughter/sister…) giving birth?
    a. How would you describe your experience? Why?

77. Satisfaction
    a. How satisfied are you with the type of care your (wife/partner/daughter/sister…) received by your (wife/partner/daughter/sister…)during labour and childbirth?
    b. Can you give me an example of something you are very satisfied with?
    c. Can you give me an example of something you are NOT satisfied with?

78. How well do you feel that your (wife/partner/daughter/sister…) healthcare provider respected your opinions about care during labour and childbirth?
    a. Can you give me an example of a time when your (wife/partner/daughter/sister…) healthcare provider respected your opinions about care during labour and childbirth?
    b. Can you give me an example of a time when your (wife/partner/daughter/sister…) healthcare provider did NOT respect your opinions about your care during labour and childbirth?
        i. Probe: Or, an example of a time during labour and childbirth when someone else was making decision without talking with you or your (wife/partner/daughter/sister…)?

79. Place of birth
    a. Was she planning that she would give birth in this facility? Why or why not?
    b. How was the decision made about where she gave birth?
        i. Who was involved in the decision-making?

80. Thinking back to your wife/partner/sister birth, what are some of the things you value most? Why?
    a. Probe: to keep her healthy?
    b. Probe: to keep the baby healthy?
    c. Probe: for yourself personally?

Part 2. Decision-making processes regarding mode of childbirth

81. Could you tell me about the different ways that women can give birth?
    a. Probe: How did you learn about these options?

82. What about for your (wife/partner/daughter/sister…) most recent birth – how did she give birth? For example, caesarean or vaginal birth?
    a. Is this the way that you preferred your (wife/partner/daughter/sister…) would give birth? Why or why not?

    b. Probe: How did you decide that you preferred her to give birth in this way?
        i. Probe: Did your (wife/partner/daughter/sister…) influence your opinion? How?
        ii. Probe: Did your family influence your opinion? How?
        iii. Probe: Did your friends influence your opinion? How?

For peer review only - http://bmjopen.bmj.com/site/about/guidelines.xhtml

iv. Probe: Did your (wife/partner/daughter/sister…) doctor or midwife influence your opinion? How?
v. Probe: Did the media influence your opinion? How?

c. Did you discuss your opinion with your (wife/partner/daughter/sister…) doctor or midwife? If so, what was the discussion like?

83. Who decided about how she would give birth?
    a. Probe: who was involved in the decision-making?
    b. Probe: Did you influence this decision? How?
    c. Probe: Did your family or her family influence this decision? How?
    d. Probe: Did your friends or her friends influence this decision? How?
    e. Probe: Did the doctor or midwife influence this decision? How?
    f. Probe: Did the media influence this decision? How?

84. How did you learn about vaginal birth and caesarean section?

85. Did you feel like you had enough information to understand the options that your (wife/partner/daughter/sister) had for how to give birth? Why or why not?

86. Did you feel like you had sufficient time to talk to your (wife/partner/daughter/sister…) doctor or midwife about any concerns that you had about your (wife/partner/daughter/sister…) labour and childbirth? Why or why not?
    a. Can you give me an example of a time when you felt that you were able to discuss your questions or concerns with your (wife/partner/daughter/sister…) doctor or midwife?
    b. Can you give me an example of a time when you felt that you were NOT able to discuss your questions or concerns with your (wife/partner/daughter/sister…) doctor or midwife?

87. Pregnancy and childbirth are exciting times but can also be scary. Is there anything that you were afraid of or nervous about the childbirth?

88. *A decision-analysis tool could help to educate women about their options for mode of birth and how to discuss their preferences with a doctor.* Interviewer: show the woman the Vietnam decision analysis tool.
    a. Would this type of tool be helpful to you? Why or why not?
    b. What type of information would you like to have included?
    c. This type of decision tool can be paper based like this example, or could be an application for a phone. Which of these options do you prefer and why?

Part 3. Labour companionship

89. Were you at the hospital with your (wife/partner/daughter/sister…) when she gave birth? Why or why not?

90. If you have gone to the hospital when your (wife/partner/ daughter/sister…) gave birth, what did you need in order to have a positive experience?

91. What type of support do you think your (wife/partner/ daughter/sister…) needed during labour and childbirth?
    a. Do you think she will received this type of support? Why or why not?

For peer review only - http://bmjopen.bmj.com/site/about/guidelines.xhtml

Interviewer read: A labour companion is a person of the woman's choice, who can help to provide emotional support to the woman during labour and childbirth. Typically, this person would be with the woman continuously throughout labour and childbirth. This person may be the woman's husband/partner, her mother, or a friend.

92. Have you ever heard of someone providing this type of support?

93. What do you think of this type of support?

94. Do you know if labour companionship was allowed in the hospital your (wife/partner/ daughter/sister…) gave birth in?
    a. *If labour companionship was not allowed*: What do you think are the reasons for not allowing a labour companion?
    b. Had your (wife/partner/sister…) be allowed a labour companion if she had requested it? Why or why not?

95. Have you ever provided this type of support before?
    a. *If yes*: Could you tell me more about this?

96. Do you think your (wife/partner/ daughter/sister…) would have wanted to have a labour companion for her birth? Why or why not?

97. If your (wife/partner/ daughter/sister…) were to have a labour companion with her, who do you think she would prefer this person to be? Why?

98. If your (wife/partner/ daughter/sister…) were to have a labour companion with her, what do you think she would expect from this person?

99. Would you be interested in being a labour companion to your (wife/partner/ daughter/sister…)? Why or why not?

100. What would you need in order to be a good labor companion?
    a. What do you need from the woman?
    b. What do you need from the nurses and doctors?
    c. What do you need from the hospital?

101. What type of information or education do you think a labour companion would need to be able to support her?
    a. When do you think a woman should start talking to a potential labour companion about their role during labour and childbirth? (probe: at what month during the pregnancy?)

102. In your opinion, what changes do you think the hospital could make in order to make it more comfortable for women to have a labour companion?

Thank you so much for your time. Is there anything else that you would like to share with me today about anything we talked about?

For peer review only - http://bmjopen.bmj.com/site/about/guidelines.xhtml

In-depth interview: providers

Step 1: Introduce yourself to the participant. Describe the purpose of the interview and how information will be used. Obtain oral/written consent.
Step 2: Complete sociodemographic information about the participant.
Step 3: Conduct the interview. Please remember to audio record the interview.

Interview place: ________________________________

Interview date: ________________________________

Start time: ____________________________________

End time: _____________________________________

Interviewer name: _______________________________

Interview identification: ___________________________

Participant information

Name of health facility currently employed at (write in): ______________________

Cadre and position (write in): ____

Number of years working at current health facility:__________________________________

Number of years as a (doctor/midwife/nurse) in total:________________________________

Age (years): ____

Marital status (Single, married/cohabitating, divorced, widowed): ___________________

For peer review only - http://bmjopen.bmj.com/site/about/guidelines.xhtml

Decision-making processes regarding mode of childbirth

1. Could you describe what you think quality care provided during childbirth is?
    a. Could you give me an example of a situation when this kind of care was provided by you?
        i. Why do you think you were able to provide quality care in this situation?
        ii. How did your colleagues support you to provide quality care?

    b. Could you give me an example of a situation when this kind of care was NOT provided by you or by a coworker?
        i. Why do you think you weren't able to provide quality care in this situation?
        ii. Did you feel like your colleagues supported you in this situation? Why or why not?

2. In your health facility, how are decisions made about whether a woman will give birth vaginally or by caesarean section?
    i. Who is involved in making the decision, and what roles do they play?
    ii. In your health facility, what are some of the clinical indications for caesarean section?
    iii. Other than clinical indications for caesarean section, what factors might influence if a woman has a caesarean section?

3. Is assisted vaginal delivery (e.g. by vacuum or forceps) used in your facility?
    i. Probe: why or why not?
    ii. Probe: were you trained on how to provide assisted vaginal delivery? Please explain.

4. In your facility, do you think that women prefer to give birth by caesarean section or vaginally? Please explain.

5. In your health facility, how do you manage women who request to have an elective caesarean section?
    i. Why do you think women may request to have a caesarean section without a medical indication (e.g. elective caesarean)?
    ii. Who do you think influences women's decisions to have a caesarean section without a medical indication (e.g. elective caesarean)?

6. As a clinician, do you prefer for women to give birth vaginally or by caesarean section? Why?
    i. What are some of the benefits/challenges of caesarean section/vaginal birth?
    ii. Which do you think is safer: vaginal birth or caesarean section? Why?

7. In your opinion, are high rates of caesarean section a problem in your health facility? Why or why not?
    i. *Probe if yes:*
        i. Why do you think there are high rates of caesarean section in your facility?
        ii. Do you think that the caesarean section rate in your facility can be reduced? Why or why not?
        iii. Do you think that the caesarean section rate in your facility should be reduced? Why or why not?
        iv. What are the barriers to reducing high rates of caesarean section in your facility?

For peer review only - http://bmjopen.bmj.com/site/about/guidelines.xhtml

v. What could be done to reduce high rates of caesarean section in your facility?

ii. *Probe if no:*

i. Do you think that the caesarean section rate in your facility could be reduced? Why or why not?

ii. Do you think that the caesarean section rate in your facility should be reduced? Why or why not?

iii. What are the barriers to reducing high rates of caesarean section in your facility?

8. From your perspective, is a caesarean section more or less work for a healthcare provider, compared to a vaginal birth? Please explain.

9. In your opinion, do you think it is financially more profitable for providers or health facilities to conduct a caesarean section, compared to a vaginal birth?
   a. *Probe:* Why or why not?

Prenatal education and decision-analysis tool

Interviewer: The next section of this interview is about the type of health education about mode of birth that you think women would like to receive during antenatal care. I would like to ask you some questions about what you think about different topics of health education to be discussed during antenatal care.

10. In your opinion, do you think that women have sufficient knowledge about their options and the risks and benefits for different mode of birth? Why or why not?

11. In your practice, how do pregnant women they access information about their options for mode of birth?
    a. What do you think about these information resources?

12. What type of information do you think that women need to inform their preferences and decisions about their mode of birth?
    a. *Probe:* Risks of different methods, benefits of different methods, personal preferences

13. Do you think that groups of women may have different needs for information about mode of birth?
    a. Probe if yes: what groups of women do you think may need different information?
    b. *Probe if yes*: What type of information do you think that these women might need?

14. During antenatal care, do you (or providers conducting antenatal care in your facility) discuss with women whether they have a preference for vaginal birth or caesarean section?
    a. *IF YES, probe:* What do you discuss with the women?
    b. *IF NO, probe:* Do you think that discussing their preferences for vaginal birth or caesarean section could be helpful? Why or why not?

15. What information do you think could be included in prenatal education about vaginal birth and caesarean section?

16. At what point during a woman's pregnancy do you think they should receive this information about mode of birth? Why?

For peer review only - http://bmjopen.bmj.com/site/about/guidelines.xhtml

17. How often do you think women should receive this information about vaginal birth and caesarean section?
    a. *Probe:* Once? More than once?

18. How would do you think women should receive this information?
    a. *Probe:* Should she receive this information verbally, from her healthcare provider? Why or why not?
    b. *Probe:* Should she receive this information in a pamphlet or brochure? Why or why not?
    c. *Probe:* Should she receive this information using a computer or a mobile phone application? Why or why not?

(Interviewer: use the Vietnam decision-analysis tool as an example)
The next section of this interview is about using decision-tools (such as a computer, tablet or a smart phone) to help pregnant women with previous caesarean sections to understand their choices about mode of birth. By this, we mean whether the woman will have planned vaginal birth, trial of labour, or a caesarean section. These decision-tools would provide them with information about potential benefits and harms of the different options. They would be in addition to any regular counselling or discussions with healthcare providers. I would like to ask you some questions about what you think about these decision-tools.

19. *A decision-tool could provide descriptions of the health outcomes associated with planned vaginal birth, planned caesarean section, and emergency caesarean section. They can also ask questions about a woman's values and preferences for possible outcomes. Once this information is provided, the decision-tool can produce a recommended "preferred option", based on a woman's preferences. The woman could then bring this to her healthcare provider to discuss in more detail.* What do you think about this type of decision-tool?
Does this description of a decision-tool sound like something that might be useful to you? Why or why not?
    a. What do you think are some of the benefits of using a decision-tool to help decide about how a woman will give birth?
        i. *Probe:* to you as a provider?
        ii. *Probe:* to the woman
    b. What do you think are some of the challenges of using a decision-tool to help decide about how a woman will give birth?
        iii. *Probe:* to you as a provider?
        iv. *Probe:* to the woman
    c. At what point during a woman's pregnancy would it be most helpful for her to have access to this type of decision-tool? Why?
    d. How might you use the results of the decision tool, or the woman's "preferred option", to discuss her options for mode of birth?
    e. Do you think that you would recommend that women use this type of decision-tool? Why or why not?

20. *These types of decision-tools can come in different formats. For example, on paper, a computer, a tablet, or a smart phone application.* What format do you think would be most helpful? Why?

Audit and feedback

Note to interviewer: use graphic of audit and feedback/Robson classification to explain to providers who don't understand. Consider using an example of maternal morbidity and mortality conference as example of audit and feedback.

For peer review only - http://bmjopen.bmj.com/site/about/guidelines.xhtml

Interviewer: The next part of the study is about ***using audit and feedback as a tool for quality improvement****. The purpose of audit and feedback is to encourage individuals and teams to follow professional standards or targets and to monitor changes and outcomes when these are used. During an audit and feedback process, an individual's or department's professional practice and/or performance is measured and compared to targets or professional standards. The results of this comparison are fed back to the individual by either a colleague, supervisor or third party, in the form of verbal or written communication. I would like to ask you some questions about what you think about audit and feedback.*

21. <u>Could you tell me about a time where you have been involved in an audit and feedback project?</u>
    a. *If they have been involved in an audit and feedback project:*
        i. What did you find helpful about the audit and feedback process?
        ii. What did you find challenging about the audit and feedback process?
        iii. What were the main things you learned from the audit and feedback process?
        iv. Overall, what was your opinion regarding the audit and feedback process?

22. <u>What areas of health do you think would be most interesting and relevant for audit and feedback? For example, this might include reasons for caesarean section, severe morbidity. Why are these interesting?</u>

Audit and feedback to improve obstetric care may include activities like critical case incident reviews, indications for caesarean section, time from decision to operation for caesarean section, decision-making processes for caesarean section, and appropriate management of complications. This may be done by reviewing individual patient records, labour and delivery logs, and observations of clinical practice.

23. <u>How would you feel about the idea of a regular audit and feedback process in your health facility to address rising caesarean section rates?</u>

24. What might be some of the benefits of audit and feedback may be related to caesarean section?

25. What might be some of the challenges of audit and feedback may be related to caesarean section?

26. Do you think starting an audit and feedback process may change people's behaviour in your department? Why or why not?

27. Do you think starting an audit and feedback process may change health outcomes? Why or why not?

28. What could be done in your health facility to ensure that audit and feedback is conducted in a supportive way that emphasises learning rather than punishing providers for certain behaviours?

29. How can audit and feedback be presented to you to ensure that any information gathered is "actionable" so that an individual can work to improve their practice?

30. <u>What type of person would be the most appropriate person to:</u>
    a. <u>Review medical records?</u>
    b. <u>Analyse the data and prepare a summary report?</u>

For peer review only - http://bmjopen.bmj.com/site/about/guidelines.xhtml

c. To present or discuss the report with you?
d. Probe: Would you prefer that this person were a colleague, supervisor, or someone external? Why?

31. Approximately how often do you think that audit and feedback processes should occur in your health facility? Please explain.

(Interviewer: do not ask these questions to antenatal nurses)
*Interviewer: The next part of the study is about the **audit and feedback tools for classifying caesarean sections**. These tools may be useful for healthcare providers and administrators to monitor which women are receiving caesarean sections, and also to help to compare caesarean section rates over time or across different health facilities and countries. This may help to design and implement interventions to make sure that an optimal caesarean section rate can occur in a specific health facility. I would like to ask you some questions about what you think of such audit and feedback tools. In order to understand drivers of rising Caesarean section rates, we need to have tools to monitor and compare caesarean section rates in a setting over time. One way to do this is the Robson classification system, which prospectively classifies women admitted for childbirth into one of ten groups.*

32. Have you heard of the Robson classification system before?
    a. *If yes:* Can you tell me what you know about the Robson classification system?
    b. *If no,* Do you know of any other classification systems to classify women giving birth?

33. *Conducting audit and feedback for caesarean section requires reviewing patient medical records and/or facility logs.* Could you tell me about how records are prepared and kept in your health facility?
    a. What is your perception regarding the completeness of labour and delivery records in your health facility?
        1. *Probe:* Do you think that labour and delivery records are complete and accurate for all or most women in your health facility? Why or why not?
    b. *Probe:* Who is responsible for recording in the medical records?
    c. *Probe:* In addition to the individual patient's record, how else is data collected and recorded on the labour and delivery ward?
    b. *Probe:* is there a facility-level logbook? If so, who is responsible for this? What type of data is recorded?

In-service training and implementation of clinical practice guidelines

34. How well do you feel your training prepared you for your current position? Please explain.

For peer review only - http://bmjopen.bmj.com/site/about/guidelines.xhtml

Interviewer: The next part of the study is about continuous training and implementation of clinical practice guidelines. This refers to the processes by which guideline recommendations are used to help healthcare providers make informed decisions about how and when to provide care in order to achieve the best health outcomes. I would like to ask you some questions about what you think about guideline implementation.

35. Are you aware of any clinical practice guidelines (algorithms/flowcharts/clinical protocols) related to obstetrics?
    a. *If yes,* which clinical practice guidelines are you familiar with?
        i. In your opinion, how valuable are the obstetrics clinical practice guidelines to your practice?
        ii. In your opinion, how accessible are these clinical practice guidelines to healthcare providers?
        iii. *Probe:* what could be done to improve the accessibility of clinical practice guidelines to other healthcare providers?
        iv. Could you describe the process of how obstetrics clinical practice guidelines are prioritised in your health facility?
        v. How are the clinical practice guidelines communicated to other healthcare providers in your facility?
        vi. How do you use clinical practice guidelines in your practice?
    b. *If no, probe:*
        i. How do healthcare providers in your facility make decisions about how to manage patients?
        ii. In your health facility, are clinical practice guidelines currently used in obstetrics?
        iii. In your opinion, what could be done to improve the accessibility of clinical practice guidelines to other healthcare providers?

36. *Imagine that your health facility will start a process of updating and implementing obstetrics clinical* practice guidelines (algorithms/flowcharts/clinical protocols). Who would need to support this initiative in order for it to be successful?
    a. *Probe:* Why would this person/these people need to support the initiative?

    b. *Probe:* How would this person/these people best support the initiative?

    c. What type of training would be helpful to ensure that all staff understand the clinical practice guidelines?
        i. What type of topics would you like to have covered during the training?
            1. Would you be interested to learn about how clinical practice guidelines were developed? Why or why not?
            2. Would you be interested to learn about the evidence behind the recommendations in clinical practice guidelines, such as the systematic reviews or clinical trials?
        ii. How long should the training last for?
        iii. How often should the training be repeated?
        iv. Where should the training be held (e.g. within the facility, outside the facility)?

    d. What resources would be needed in order to successful implement obstetrics clinical protocols?

For peer review only - http://bmjopen.bmj.com/site/about/guidelines.xhtml

e. In your opinion, what are some barriers to successful implementation of obstetrics clinical practice guidelines?

f. In your opinion, what are some facilitators to successful implementation of obstetrics clinical practice guidelines?

g. *Usually when clinical practice guidelines are implemented in health facilities, there are activities to evaluate if the guidelines are being implemented correctly and consistently*. What type of evaluation activities would be helpful to assess if obstetrics clinical practice guidelines were being implemented correctly and consistently?
   i. What format would be appropriate to feedback the evaluations to healthcare providers?
   ii. If meetings were held to feedback on the progress of obstetric clinical practice guidelines implementation, what would you like to hear discussed?
      1. Who would attend these meetings and why?
      2. How often would these meetings be held?

37. In your opinion, how important is providing pain relief for women during labour (vaginal birth only)? Why?

   a. In your facility, what pain relief options are there for women during labour (vaginal birth only)? Probe: pharmacological and non-pharmacological methods

   b. In your opinion, how important is it for women to walk around during labour (vaginal birth only)? Why?

   c. In your opinion, how important is it for women to be able to sit upright during labour (vaginal birth only)? Why?

Opinion leader education

Interviewer: The next part of the study is about using opinion leaders in a specific health facility to act as champions for change. Opinion leaders are influential individuals who are nominated by their peers to change the culture and norms of healthcare provider peer groups. For example, these individuals may be responsible for adapting clinical guidelines to a specific health facility context, and identifying measures to ensure quality improvement. I would like to ask you some questions about what you think about the use of opinion leaders in your health facility.

38. What do you think are the characteristics of a good opinion leader?

39. What do you think about the idea of using opinion leaders to adapt clinical guidelines to your health facility?

40. What type of healthcare provider would be most appropriate to act as an opinion leader for caesarean section? *(probe: nurse/midwife/doctor, what level of training)*

41. How do you think an opinion leader would be received by other healthcare providers in your health facility?

For peer review only

For peer review only - http://bmjopen.bmj.com/site/about/guidelines.xhtml

42. What challenges do you think an opinion leader would face if they tried to adapt and implement clinical guidelines in your health facility?

43. What type of *training* would an opinion leader need to succeed?

44. What resources would an opinion leader need to succeed?

Organization and relationships in the facility

45. Could you please describe for me what the relationship that you have with your peers is like?
    a. Could you tell me about a time when your peers supported you?
    b. Could you tell me about a time when your peers did not support you?
    c. If you are struggling to meet the demands of your work, can you look to your peers for help? How so?
    d. In your opinion, are men and women treated equally in your work place? Why or why not?

46. In your opinion, how well do doctors and midwives work together in general?
    a. How well do you think doctors and midwives communicate?
    b. What are some of the challenges in having midwives and doctors work together?
    c. Can you tell me about a time when midwives and doctors did NOT work together?
        i. Why do you think this happened?
    d. Can you tell me about a time when midwives and doctors worked very well together?
        i. Why do you think this happened?

47. Overall, how supportive do you feel that your work environment is? Please explain.

48. How do you feel about the current environment around malpractice lawsuits and legal liability for doctors?
    a. Do you feel that the health system or your health facility would support you in a legal case?
    b. How do you think that the legal environment may influence your own, or your colleagues', medical practice?

49. Do you feel afraid of malpractice lawsuits in your current work? Please explain.

50. What strategies do you employ to minimise the risk of a malpractice lawsuit?
    a. Do you think these strategies are reasonable?

For peer review only

For peer review only - http://bmjopen.bmj.com/site/about/guidelines.xhtml

Labour companionship

Interviewer: The next part of the study is about the type of support that women could receive during childbirth in a health facility. In some settings, a "labour companion" can provide this type of support. A labour companion is a person of the woman's choice, for example her husband, her sister, her mother, her friend, or a doula, who stays with the woman throughout the duration of labour and childbirth. I would like to ask you some questions about what you think about support from a companion during childbirth.

51. <u>What type of support do you think women need during labour and childbirth?</u>
    a. Do you think that women in your hospital receive this kind of support you have described? Why or why not?

52. <u>What do you know about labour companionship?</u>
    a. What are the benefits of labour companionship?
        i. *Probe*: What are benefits for the woman?
        ii. *Probe*: What are benefits for the providers?
        iii. *Prove*: What are benefits for the companion?
    b. Are there any harms of labour companionship?
        i. *Probe*: What are harms for the woman?
        ii. *Probe*: What are harms for the providers?
        iii. *Probe*: What are harms for the companion?

53. <u>Do you have any previous experience with working in a hospital that offered labour support?</u>
    i. If yes, what was this experience like for you as a provider?

54. <u>Do you know if labour companionship is allowed in this hospital?</u>
    a. *If labour companionship is not allowed:* What do you think are the reasons for not allowing a labour companion in this hospital?

55. <u>How could labour companionship be implemented in your hospital or other hospitals like this?</u>
    a. What would be the main challenges to implementing labour companionship?
    b. Who do you think women would prefer as a labour companion? Why?
    c. As a provider, what are your expectations from a woman's labour companion?
    d. When would a labour companion be able to be with the woman in the hospital?
    e. What would the role of the labour companion be?
        i. How could the labour companion's roles be communicated to them?
    f. At what point during the care process should women and providers start talking about labour companionship and the role of the companion?
    g. What type of information or education do you think a labour companion would need to be able to support you?
    h. How could we ensure that the companion is a person of the woman's choice, and not someone selected for her by someone else?
    i. What changes do you think the hospital could make to make it more comfortable for women to have a labour companion?
    j. If labour companionship is to be implemented in this hospital, what would ensure successful implementation?
        a. What could be done to ensure that labour companionship was sustainable in the long-term?

For peer review only - http://bmjopen.bmj.com/site/about/guidelines.xhtml

Appendix 2. Readiness assessment

1.	Name of hospital	
2.	Hospital code	

This activity is part of the readiness assessment, to explore factors to be assessed, considered and integrated into implementation plans. There are five components of this readiness assessment:

1. *Inventory of physical space and readiness;*
2. *Health workforce and model of care;*
3. *Protocols and guidelines for managing clinical care during labour and childbirth;*
4. *Continuous education and quality improvement*
5. *Assessment of data availability and access for audit and feedback; and*
6. *Understanding of labour companionship in practice.*

For each of the study health facilities, please have a member of the research team visit to conduct an observation of the labour ward and medical records. It may be most appropriate for this person to have some clinical knowledge. We expect that this activity will take approximately four to six hours to complete (depending on how busy the facility is). Prior to conducting the readiness assessment, please ensure that all members of the maternity care unit at each health facility are briefed on the purpose of the activity, what the readiness assessment will entail, and how they may be of assistance. This will help to ensure that the readiness assessment (and other research activities) will be welcomed by the unit.

Initial inventory

3.	# of Deliveries (1 October 2019 – 31 January 2020)	
4.	No of delivery handled exclusively by a certain obstetrician (private)	
5.	Women not handled exclusively by a certain obstetrician (non-private)	
6.	No. of Caesarean section (1 October 2019 – 31 January 2020)	
7.	Elective (pre-labour c-section)<br>*Note: if this information is not readily available, then please ignore for now.*	
8.	Emergency (intrapartum c-section)	
9.	No. of vaginal instrumental delivery (1 October 2019 – 31 January 2020)	

For peer review only - http://bmjopen.bmj.com/site/about/guidelines.xhtml

Part 1. Inventory of physical space and resources

Please observe the physical space of the labour, delivery and postnatal wards. If these are in separate areas (e.g.: women in latent labour in a labour ward, women in active labour in a separate room/delivery ward, separate postnatal ward), please assess both areas according to all points below. Please provide a narrative description of the wards, as well as a visual depiction.

Description of the physical space

Please detail:

# of beds in labour /admission room (1st stage of labour room):	
# of beds in labour / childbirth room (2nd stage of labour room):	
# Ultrasound machine in the labour room (indicate number working)	
# Electronic fetal heat rate monitor (CTG) (indicate number of devices working)	

Description of any curtains, dividers or other means of protecting a woman's privacy

Description of the potential for crowding. For example, how many beds are present? Are they currently or usually full? What happens if there is overcrowding?

For peer review only - http://bmjopen.bmj.com/site/about/guidelines.xhtml

Description of the visiting hours and allowable visitors (check if visually displayed and ask an administrator) (please take a photo if there is a sign)

Description of the accessibility to toilets or washrooms

Description of overnight accommodation for family members/friends of women (check if visually displayed and ask an administrator)

Description of the **operating theatres** (how is access to the theatre, is there one theatre reserved for obstetrics, is the theatre on the same floor? How are handwashing facilities)

Please detail how many theatres are exclusively to perform a CS (#)	

For peer review only - http://bmjopen.bmj.com/site/about/guidelines.xhtml
For peer review only

Please sketch the physical space

For peer review only - http://bmjopen.bmj.com/site/about/guidelines.xhtml

Part 2. Health workforce and model of care.

Completing this section may require both observation of the labour ward and a discussion with staff, e.g.: a matron-in-charge or head of obstetrics.

Please indicate below the **staffing available for the delivery ward alone. Please include all staff on the payroll**. Please do not count the staff only working on ANC or PNC. If staff works part-time in the maternity, try to summarise to have full-time-equivalents.

STAFFING – LABOUR AND DELIVERY WARD ONLY		
	**Staff category**	Number employed (Full-time equivalent)
1.	Obstetrician	
2.	Anaesthesiologists	
3.	Paediatrician	
4.	Medical doctors (graduated, no specialization)	
5.	Residents (medics in training)	
6.	Nurses / nurse-midwives	
7.	Distinct midwives (exclusively trained on midwifery)	
8.	Delivery assistants / auxiliary nursing staff	
9.	Cleaners / other auxiliary non-nursing staff	

STAFFING ON SHIFTS IN LABOUR AND DELIVERY			
	**Staff category**	**DAY SHIFT** Number employed (Full-time equivalent)	**NIGHT SHIFT** Number employed (Full-time equivalent)
1.	Obstetrician		
2.	Anesthesiologists		
3.	Pediatrician		
4.	Medical doctors (graduated, no specialization)		
5.	Residents (medics in training)		
6.	Nurses / nurse-midwives		
7.	Distinct midwives (exclusively trained in midwifery)		
8.	Delivery assistants / auxiliary nursing staff		
9.	Cleaners / other auxiliary non-nursing staff		

For peer review only - http://bmjopen.bmj.com/site/about/guidelines.xhtml

Please indicate below the **staffing available for the <u>ANTENATAL CARE ward</u> alone. Please include all staff on the payroll**. Please do not count the staff only working on ANC or PNC. If staff works part-time in the maternity, try to summarise to have Full-time-equivalents.

STAFFING – ANTENATAL CARE WARD ONLY		
	**Staff category**	Number employed (Full-time equivalent)
1.	Obstetrician	
2.	Anesthesiologists	
3.	Pediatrician	
4.	Medical doctors (graduated, no specialization)	
5.	Residents (medics in training)	
6.	Nurses / nurse-midwives	
7.	Distinct midwives (exclusively trained on midwifery)	
8.	Delivery assistants / auxiliary nursing staff	
9.	Cleaners / other auxiliary non-nursing staff	

STAFFING ON SHIFTS IN ANTENATAL CARE WARD			
	**Staff category**	**DAY SHIFT** Number employed (Full-time equivalent)	**NIGHT SHIFT** Number employed (Full-time equivalent)
1.	Obstetrician		
2.	Anesthesiologists		
3.	Pediatrician		
4.	Medical doctors (graduated, no specialization)		
5.	Residents (medics in training)		
6.	Nurses / nurse-midwives		
7.	Distinct midwives (exclusively trained in midwifery)		
8.	Delivery assistants / auxiliary nursing staff		
9.	Cleaners / other auxiliary non-nursing staff		

For peer review only - http://bmjopen.bmj.com/site/about/guidelines.xhtml

Please explain the on-call system: Is the doctor to perform a CS in the hospital even at night, or is s/he on-call at home? Are there other resource-persons one can call in if needed?

Part 3. Protocols and guidelines for managing clinical care during labour and childbirth.

Please list the protocols available and in use in this hospital. Please indicate if the head of maternity indicates the presence. If so ask about the type of guidelines (national standard guidelines e.g by MoH or professional organisation) and if the guideline is physically available, e.g in a folder or displayed at the wall.

**S. No**	**List of Protocols**	Present<br>1. Yes<br>2. No	Type<br>1. National Standards<br>2. Hospital Specified	Displayed<br>Yes<br>No
1.	Partograph use / fetal monitoring			
2.	Active Management of Third Stage of labor			
3.	Postpartum haemorrhage management			
4.	Blood transfusion			
5.	Pre-term labor			
6.	Induction / augmentation of labour			
7.	Antenatal steroids			
8.	Obstructed labour			
9.	Previous CS (trial of labour)			

For peer review only - http://bmjopen.bmj.com/site/about/guidelines.xhtml

Completing this section may require both observation of the labour ward (e.g.: posters or signs) and a discussion with staff, e.g.: a matron-in-charge or head of obstetrics. If possible, make a photo if there are guidelines on the wall.

Could you describe how any clinical protocols or guidelines for managing *routine or complicated labour and childbirth* care were developed or adapted, and updated? Is there a team in the hospital taking care of this? Can you explain how are these clinical protocols/guidelines used?

Continuous education and quality improvement

Specify for trainings provided in the last 1 year

**List of training provided**	Type<br>1. National Standard training<br>2. Hospital Specified	Was the training provided to<br>1. All providers<br>2. Only doctors<br>3. Only nurse-midwives
Partograph use / fetal monitoring		
Active Management of Third Stage of labour		
Postpartum haemorrhage management		
Blood transfusion		
Pre-term labor /		
Introduction / augmentation of labour		
Antenatal steroids		
Obstructed labour		
Previous CS (trial of labour)		

For peer review only

For peer review only - http://bmjopen.bmj.com/site/about/guidelines.xhtml

Could you please describe other quality improvement activities ongoing in this facility. How are nurses and doctors informed about new knowledge and guidelines? Do you need to go regularly to refresh knowledge to workshops or trainings?

Do you do on-the-job training / mentoring of younger colleagues? Is there a system of supervision? Is there a system to discuss difficult cases, e.g. during a morning report? Do you do audits of cases?

Please explain how informed consent is obtained for caesarean section (oral, written, included in medical record, etc).

For peer review only

For peer review only - http://bmjopen.bmj.com/site/about/guidelines.xhtml

Part 4. Assessment of facility medical records and data management systems

Please review individual medical records to assess the information currently collected related to key obstetric variables at an individual level. Please also review any facility-level register, log book or other records to assess information currently collected related to key obstetric variables at a facility level. It may be helpful to discuss the medical and facility records with the staff, e.g.: a matron-in-charge or head of obstetrics. Collecting this information will help to inform the implementation of the Robson classification system, e.g.: to identify what data is already routinely collected, and what data may need to be added to routine data collection.

Please explain which information is used. Description of other information routinely collected about caesarean section (e.g. provider, morbidity)

There are typically two different places for documentation a) facility-level register, log book, or other records collating key obstetric variables and b) case notes/patient records. Now first we like to have information on the first type:

Please describe the description of any facility-level register, log book, or other records collating key obstetric variables at the facility-level. Please include whether this register is paper-based or electronic, when it is updated, when and how information is summarised and how often it is reported. Please take a photo (covering patient names).

Who is responsible for completing the facility-level register?

For peer review only - http://bmjopen.bmj.com/site/about/guidelines.xhtml

For peer review only

How often is the facility-level register updated and summaries are prepared?

How is the information about facility-level key obstetric variables and outcomes currently integrated into audit and feedback?

Description of the consistency of reporting for these indicators (e.g.: consistently reported across all records reviewed, some data missing – be specific).

Who is present during audit and feedback sessions, and who leads the sessions?

For peer review only - http://bmjopen.bmj.com/site/about/guidelines.xhtml

Description of the health facility's "decision-to-incision" time to perform a caesarean section. If not available, please specify.

Now concerning the second type of records: case notes/medical records: Please describe the medical record structure (e.g. electronic or paper), and who keeps the records (e.g. woman or provider)? Is there a standard form? Is the WHO partograph used? Are the data used for audits and feedback?

Is there any regular feedback of these reports to the providers? If so, how often and in what format?

Review of medical records to assess if key obstetric variables needed for Robson classification are correctly and consistently reported at an individual level. For each variable, please (1) ask the administrator how it is reported, and (2) observe a subset of records to assess how variable is actually reported (e.g. 5-10 medical records).

Parity	
**Administrator response**	**Observation of records**

For peer review only - http://bmjopen.bmj.com/site/about/guidelines.xhtml

Previous caesarean section	
Administrator response	Observation of records

Onset of labour (spontaneous, induced, no labour/pre-labour caesarean section)	
Administrator response	Observation of records

Gestational age (preterm <37 weeks, term ≥ 37 weeks)	
Administrator response	Observation of records

Fetal presentation or lie (cephalic, breech, transverse)	
Administrator response	Observation of records

Number of fetuses (singleton, multiple)	
Administrator response	Observation of records

Who is responsible for completing the individual-level medical records? Does anyone else check for consistent and correct reporting?

For peer review only - http://bmjopen.bmj.com/site/about/guidelines.xhtml

Based on the observations and record assessment, what would you consider to be the most appropriate method of implementation of the Robson classification system (manually, using a spreadsheet or automatic calculator, or via electronic records)? Please explain.

[*Ask the administrator*] What type of person would be the most appropriate facility-level champion to implement the Robson classification system, and why? [e.g.: type of provider, what skills this person would have]

[*Ask the administrator*] Who is the best person to ***record*** data for Robson classification, and why?

[*Ask the administrator*] Who is the best person to ***analyse*** data for Robson classification, and why?

For peer review only - http://bmjopen.bmj.com/site/about/guidelines.xhtml

For peer review only

[*Ask the administrator*] Who is the best person to *report and present* data for Robson classification, and why?

Part 5. Understanding of labour companionship in practice

Completing this section may require both observation of the labour ward and a discussion with staff, e.g.: a matron-in-charge or head of obstetrics. If companionship is not currently allowed at the facility, please specify below.

Description of who is currently allowed to act as a companion for the woman

Description of for what periods of time companionship is offered (e.g.: from admission to discharge, during labour but not childbirth, only at childbirth)

Please detail at what stages / and time of the day companions are allowed:

Labour / first stage	Yes / no
Delivery / second stage	Yes / no
Immediate postpartum period (first hour or two)	Yes / no
Postnatal ward	Yes / no
Day-time	Yes / no
Night-time	Yes / no

For peer review only - http://bmjopen.bmj.com/site/about/guidelines.xhtml
For peer review only

Description of the roles that companions usually undertake (e.g.: emotional support, providing food/water/tea to the woman, supporting staff)

Description of how staff currently interact with companions

Existence and content of any orientation materials, protocols, or guidelines related to how staff should work with companions, or on the role of companions. If no materials exist, please state this.

Any other feedback, observations or reflections

For peer review only - http://bmjopen.bmj.com/site/about/guidelines.xhtml
